# Loss of lag-response curvilinearity of indices of heart rate variability in congestive heart failure

**DOI:** 10.1186/1471-2261-6-27

**Published:** 2006-06-12

**Authors:** Tushar P Thakre, Michael L Smith

**Affiliations:** 1 Department of Integrative Physiology, University of North Texas Health Science Center, Fort Worth, Texas, USA; 2 Lata Medical Research Foundation, Nagpur, India

## Abstract

**Background:**

Heart rate variability (HRV) is known to be impaired in patients with congestive heart failure (CHF). Time-domain analysis of ECG signals traditionally relies heavily on linear indices of an essentially non-linear phenomenon. Poincaré plots are commonly used to study non-linear behavior of physiologic signals. Lagged Poincaré plots incorporate autocovariance information and analysis of Poincaré plots for various lags can provide interesting insights into the autonomic control of the heart.

**Methods:**

Using Poincaré plot analysis, we assessed whether the relation of the lag between heart beats and HRV is altered in CHF. We studied the influence of lag on estimates of Poincaré plot indices for various lengths of beat sequence in a public domain data set (PhysioNet) of 29 subjects with CHF and 54 subjects with normal sinus rhythm.

**Results:**

A curvilinear association was observed between lag and Poincaré plot indices (SD1, SD2, SDLD and SD1/SD2 ratio) in normal subjects even for a small sequence of 50 beats (p value for quadratic term 3 × 10^-5^, 0.002, 3.5 × 10^-5 ^and 0.0003, respectively). This curvilinearity was lost in patients with CHF even after exploring sequences up to 50,000 beats (p values for quadratic term > 0.5).

**Conclusion:**

Since lagged Poincaré plots incorporate autocovariance information, these analyses provide insights into the autonomic control of heart rate that is influenced by the non-linearity of the signal. The differences in lag-response in CHF patients and normal subjects exist even in the face of the treatment received by the CHF patients.

## Background

Poincaré plot is an intuitive and commonly used method to assess complex non-linear behavior in the study of physiological signals [1-6]. In the assessment of heart rate variability (HRV), Poincaré plots are constructed by plotting duplets of successive R-R intervals [1,2,4-6], with an implicit assumption that the next R-R interval is significantly determined by the current one. This assumption lends itself to further generalization of Poincaré plots by plotting *m*-lagged plots where *m *represents the distance (in number of beats) between the duplet beats, that is, the 'lag' of the second beat from the first [2]. It has been observed in the context of the short term variability that the current R-R interval can influence up to approximately eight subsequent R-R intervals [2]. Therefore, a series of lagged Poincaré plots can potentially provide more information about the behavior of Poincaré plot indices in health and disease than the conventional 1-lagged plot does [2].

Heart rate variability analysis provides a noninvasive means to assess the autonomic status of the heart [6-8]. Under normal conditions, the feedback elements characterized by vagal and sympathetic activation of the heart combined with the cardiac automaticity determine the HRV [7]. In various clinical conditions in which the sympathovagal balance is disturbed, such as after an episode of myocardial infarction, in diabetic autonomic neuropathy and in congestive heart failure, HRV is usually reduced [7-10]. In the context of congestive heart failure (CHF), the decrease in HRV has also been observed to correlate with disease severity [11-15]. However, two issues relating to the strategies employed for HRV analysis deserve closer scrutiny. First, the majority of methods of quantifying HRV (including conventional Poincaré plots) use successive R-R interval duplets only, with the implicit assumption that the current beat is influenced by the immediately preceding beat. However, it has been reported that a heart beat influences not only the beat immediately following it, but also up to 6–10 beats downstream [2], possibly as a consequence of respiratory sinus arrhythmia. Thus, an analysis hinging on the use of only successive R-R interval duplets will likely underestimate the role of the autocovariance function of R-R intervals i.e., the ability of heart beats to influence a train of succeeding beats. Second, the Poincaré plot indices relating to short-term and long-term variability in R-R intervals do not capture the non-linear disposition of HRV [16]. The autocovariance function of R-R intervals captures the additional aspects of HRV (e.g. non-linearity) that can be masked by the strong correlation between successive beats if 1-lagged plots are used. Indeed, Brennan *et al *[16] argue that lagged Poincaré plots can fully describe the autocovariance as well as the power spectrum of HRV. Our proposed analysis uses lagged Poincaré plots to overcome the limitations of the present practice of time-domain analysis of HRV.

Therefore, we hypothesized that the lag-response patterns of linear and non-linear Poincaré plot indices would be different in a diseased heart as compared to a normal heart. To test our hypothesis, we compared long-term ECG recordings of a group of CHF subjects with those of normal subjects. We also explored whether linear versus non-linear indices of HRV behaved differentially with respect to the lag. Finally, we compared the lag-responses of CHF patients and normal subjects in light of the fact that heart rate variability may have been potentially restored by the pharmacologic therapy in the CHF patients. Thus, we suggest an alternative analytic strategy that has the potential to provide unique insights into the pathophysiological basis of CHF.

## Methods

### Study subjects

We used the PhysioNet internet resource which is a large repository of various physiologic signals including electrocardiograms recorded by a 24-hour Holter monitor [17,18]. From this repository, we chose the data sets which included information on inter-beat R-R intervals. The chf2db database included records on 29 individuals with congestive heart failure. These data come from two previous trials of long-term digoxin [19] and carvedilol [20] therapy. These subjects included eight men and 2 women (gender was unknown in the remaining 21 subjects) aged 34 – 79 years. There were 4 subjects belonging to the NYHA class I, 8 belonged to NYHA class II and 17 to NYHA class III. For comparison, we used a set of ECG recordings from 54 healthy subjects with normal sinus rhythm. These subjects included 30 men (aged 28.5 – 76 years) and 24 women (aged 58 – 73 years). Complete details of the study subjects are provided in the Supplementary Table 1 (see additional file 1).

### Extraction of N-N intervals

The databases in the PhysioNet repository contain 24-hour Holter ECG recordings in two data formats; unaudited beat-wise annotations sampled at a frequency of 128 samples per second and the corresponding header identification information. The WFDB software package provided by the PhysioNet resource was used to extract beat-wise R-R intervals. We used the "ann2rr" command to extract a sequence of up to 50,000 normal beats (approximately 12 hours of ECG recording) to estimate the normal-to-normal (NN, by specifying the N option in the command) intervals in seconds.

### Study variables

**Figure 1:**
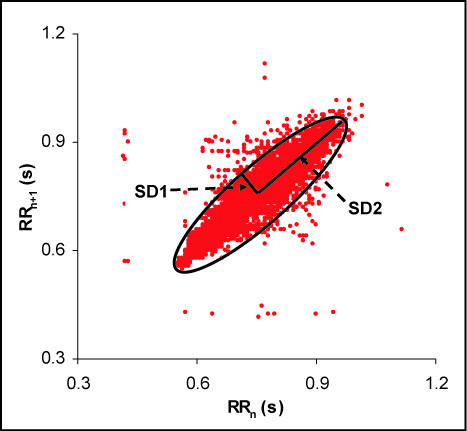
**A typical Poincaré plot**. The abscissa represents the RR interval of the current normal beat and ordinate represents the RR interval of the succeeding normal beat. An ellipse is fitted to the data points and the Poincaré plot indices are calculated by estimating the short diameter (SD1), the long diameter (SD2) and the ratio of the short and long diameters (SD1/SD2 ratio) of the fitted ellipse.

The standard measures of heart rate and its variability in the time domain are considered linear measures [16]. These include the average R-R interval (Mean RR) and its standard deviation (SDRR) [21]. Another approach is to use the differences in successive R-R intervals (successive deltas) and estimate the standard deviation (SDSD) of these deltas [21]. In a Poincaré plot, one of several quantitative approaches includes the fitting of an ellipse to the data points and estimating the short and long axes of the ellipse (Figure 1) [2,16]. The minor axis is proportional to the standard deviation of the successive differences (SD1) whereas the major axis is proportional to the standard deviation of the current beat (SD2) [2,16]. It has been recognized that the minor axis is a measure of the short-term variability while the major axis is a measure of the long-term variability of the signal [2,16]. Thus, SD1 and SD2 together capture the total variability in a data set. Given the non-linear disposition of the Poincaré plot, it has been a convention to treat SD1 and SD2 as the non-linear indices of HRV. However, it can be shown mathematically that SD1 is a function of SDSD while SD2 is a function of SDRR and SDSD [16]. As stated earlier, both SDRR and SDSD are linear indices and, by extension, SD1 and SD2 also behave mathematically as measures of the linear component of variability even though they are based on Poincaré plots [16]. Non-linear transformations of SD1 and SD2 have also been used to better capture the complete nature of HRV. For example, the ratio of SD1 and SD2 indicates the ratio of short-term versus long-term variability and has been observed to be more informative than either of these indices alone [2].

For our analyses, we included only those indices that mathematically depend on the lag. As Mean RR and SDRR are theoretically independent of lag, we did not include these indices for studying the lag-response. However, mean deltas and SDSD can be appropriately generalized to measure the lag response. For instance, we estimated deltas as the difference between the *m*-lagged R-R intervals. Thus, we were able to estimate *m*-lagged deltas and their standard deviations (SDLD). In the special case of 1-lagged beats, the mean deltas and SDLD are respectively equal to mean successive delta and SDSD. We, then, estimated the SD1 and SD2 for each of the *m*-lagged beat sequence. Thus, we included the following four indices in our analysis: SDLD, SD1, SD2 and SD1/SD2 ratio.

### Length of beat sequence used for analysis

Controversy exists with regard to the period of time for which ECG recordings should be monitored to best capture the HRV dynamics. While some researchers maintain that recordings less than 18 hours are insufficient [15], others have observed that short-term R-R interval recordings are as reliable and accurate as long-term recordings in analyzing HRV [22]. Therefore, we studied the lag-response of HRV for different lengths of beat sequences. We used the following seven lengths of consecutive beats: 50, 100, 500, 1000, 5000, 10000, and 50000. These sequence lengths represent recordings ranging from ~1 minute to ~12 hours, depending on the overall heart rate. We, thus, used the following analytical strategy: for each subject included in the study, we used seven lengths of beat sequences. For each beat sequence, we used lag values from one to ten. For each value of lag we constructed a Poincaré plot and estimated SDLD, SD1, SD2, and SD1/SD2 ratio. For construction of Poincaré plots as well as for estimation of the Poincaré plot indices, we used normal beats only as annotated in the PhysioNet database resource.

### Analysis of lag-response

Considering the facts that *m*-lagged Poincaré plots can describe the autocovariance function, the autocovariance function monotonically decreases with increasing lag for values of lags less than 10, and that the current beat influences only about six to eight successive beats in the context of a short range influence, we expected a pattern of lag-response which is stronger at the lower values of lag and which attenuates with increasing lag. In normal subjects, therefore, we expected a curvilinear relationship that explains variations in Poincaré plot indices on the basis of lag. To test for curvilinearity we used a quadratic relationship model and plotted the estimates of SDLD, SD1, SD2, and SD1/SD2 ratio against lag. We then fitted a second-order polynomial curve using the least-squares method. We assessed the model-fit using R^2 ^values. We then compared the average coefficients of the quadratic term in the second-order polynomial equations in subjects with and without CHF.

### Statistical analyses

We compared the linear and non-linear *m*-lagged Poincaré plot indices in CHF and normal groups by using Mann-Whitney rank sum test. To study the potential contribution of varying beat sequence length to estimates of the Poincaré plot indices, we used the non-parametric method of Spearman's correlation coefficient (ρ). The curvilinearity of the lag-response was assessed by using a second order polynomial regression and the model-fit was assessed using the R^2 ^value in order to quantify the amount of variation explained by the quadratic lag-response of the Poincaré plot indices. We wrote dedicated routines in Visual Basic^® ^for estimation of *m*-lagged Poincaré plot indices. Then, we used Stata 7.0^® ^for statistical analysis. For all statistical analysis, we assumed statistical significance as p < 0.05.

## Results

### Length of ECG recording and Poincaré plot indices

We first assessed the role of length of ECG recording in measurement of the Poincaré plot indices. Table 1 provides a summary of the estimates of Poincaré plot indices of the study population. We observed that the short-term variability captured by SD1 consistently tended to be higher in subjects with CHF as compared to subjects with normal sinus rhythm across varying lengths of beat sequence. This difference reached statistical significance only when the length of the sequence was at least 5000 beats (p ≤ 0.0043). The long-term variability revealed by SD2, however, showed interesting relationship with the length of the beat sequence. For sequence lengths of 50 and 100, the long-term variability tended to be higher in subjects with CHF than that in normal subjects. For sequence length of 500 beats and higher, the long-term variability tended to be less in CHF patients as compared to that in normal subjects. This difference just reached statistical significance (p = 0.0449) at beat sequence length of 1000. However, for beat sequence lengths 5000 and higher, the difference was highly significant (p ≤ 0.0047). Thus, our results indicated a critical change in statistical significance at the sequence length of 5000 beats. Estimates of short-term variability increased with sequence length both in CHF patients and in normal subjects and the increase was statistically comparable in these two groups (p = 0.6577). The long-term variability showed a stronger direct association with beat sequence length. Moreover, the Spearman correlation coefficient was significantly higher in normal subjects as compared to the CHF patients (p = 7 × 10^-5^). In spite of a non-significant difference between CHF patients and normal subjects for both SD1 and SD2 in sequences less than 5000 beats in length, the ratio of these two indices (SD1/SD2) in the same sequences showed a highly statistically significant difference with the ratio being consistently higher in CHF patients (p ≤ 2.5 × 10^-6^) (Table 1).

**Table 1:** Summary of Poincaré plot indices in study subjects.*

Beat sequence length	SD1			SD2			SD1/SD2 ratio		
	
	CHF	Normal	p	CHF	Normal	p	CHF	Normal	p
50	0.0474 (0.636)	0.0222 (0.0319)	0.2270	0.0600 (0.0677)	0.0531 (0.0342)	0.2144	0.7503 (0.3518)	0.4013 (0.2305)	2 × 10^-6^
100	0.0496 (0.0605)	0.0228 (0.0315)	0.1150	0.0640 (0.0619)	0.0595 (0.0302)	0.2215	0.6949 (0.3212)	0.3660 (0.2447)	3 × 10^-6^
500	0.0541 (0.0626)	0.0249 (0.0247)	0.0696	0.0779 (0.0627)	0.0790 (0.0351)	0.2517	0.6640 (0.3278)	0.3181 (0.2086)	9 × 10^-6^
1000	0.0505 (0.0559)	0.0248 (0.0224)	0.1004	0.0784 (0.0576)	0.0907 (0.0367)	0.0449	0.6243 (0.3285)	0.2727 (0.1726)	2.5 × 10^-6^
5000	0.0467 (0.0366)	0.0231 (0.0169)	0.0043	0.0817 (0.0427)	0.1082 (0.0354)	0.0047	0.5534 (0.2532)	0.2146 (0.1240)	4 × 10^-9^
10000	0.0483 (0.0341)	0.0221 (0.0146)	0.0008	0.0835 (0.0401)	0.1125 (0.0333)	0.0042	0.5457 (0.2328)	0.1967 (0.0972)	2 × 10^-10^
50000	0.0477 (0.0310)	0.0253 (0.0196)	0.0003	0.0982 (0.0558)	0.1522 (0.0519)	6 × 10^-6^	0.5094 (0.2394)	0.1692 (0.0923)	4 × 10^-10^
Spearman's rho	0.1766	0.2154	0.6577	0.3157	0.6643	7 × 10^-5^	-0.2395	-0.4900	0.0042

* Values indicate averages (standard deviations) in seconds.

### Lagged Poincaré plots in CHF and normal sinus rhythm

**Figure 2:**
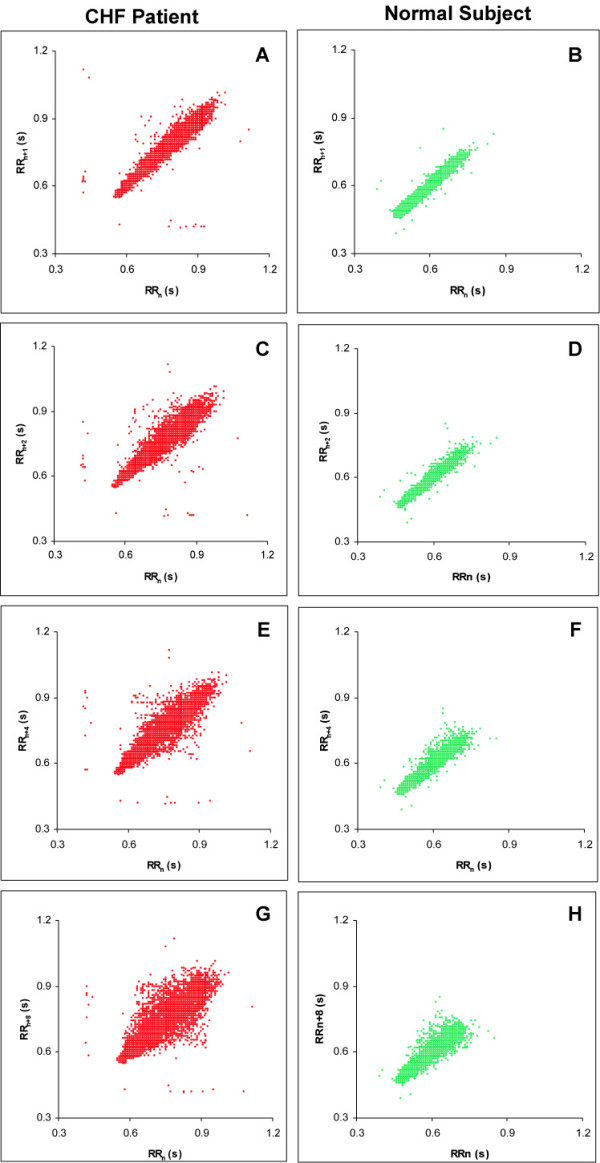
**Representative Poincaré plots from two subjects**. Panels A, C, E, and G are from a patient with CHF and panels B, D, F, and H are from a normal subject. For each subject the Poincaré plots use varying lags of 1, 2, 4 and 8 beats. The spread of the data points can be observed to increase for increasing lag for each subject. All plots are based on normal-to-normal RR intervals. It can also be noted that in this particular pair of subjects, the variability was more in the subject with CHF than that in the normal subject.

In general, we observed that the estimates of the traditional HRV measures in CHF patients were higher than those in the subjects with normal sinus rhythm. Figures 2A and 2B show representative Poincaré plots for two subjects – one with CHF (Figure 2A) and one normal subject (Figure 2B). It is apparent from the plots that the variability was more in the CHF patient as compared to the normal subject. To understand the reasons for the increased HRV in the CHF group, we first examined hourly Poincaré plots for all subjects using all the recorded beats. The results are highly interesting (supplementary material files CHF_Binder and NSR_Binder, see additional files 2 and 3). As per the classification proposed by Woo *et al *[4], most of the control subjects (51 out of 54) had the normal comet shaped Poincare plots during each hour in a 24-hour period. However most (25 out of 29) of the CHF patients had complex Poincare plots for most of the 24-hour recordings. As pointed out by Woo *et al *[5], CHF patients have either torpedo shaped or complex plots. The Poincaré plots of the CHF patients in this study best fall within the complex category.

To illustrate the putative influence of lag on estimates of Poincaré plot indices, we modified the conventional Poincaré plot by plotting (n+m)^th ^beat on the ordinate against the n^th ^beat on the abscissa. Figures 2C–2H depict this technique. It is evident from these Poincaré plots that the scatter of points increases with increasing lag. This indicates that increasing lag corresponds to increasingly unrelated beats. Our hypothesis attempted to characterize this behavior in normal subjects and CHF patients.

### Curvilinearity of lag-response

**Figure 3:**
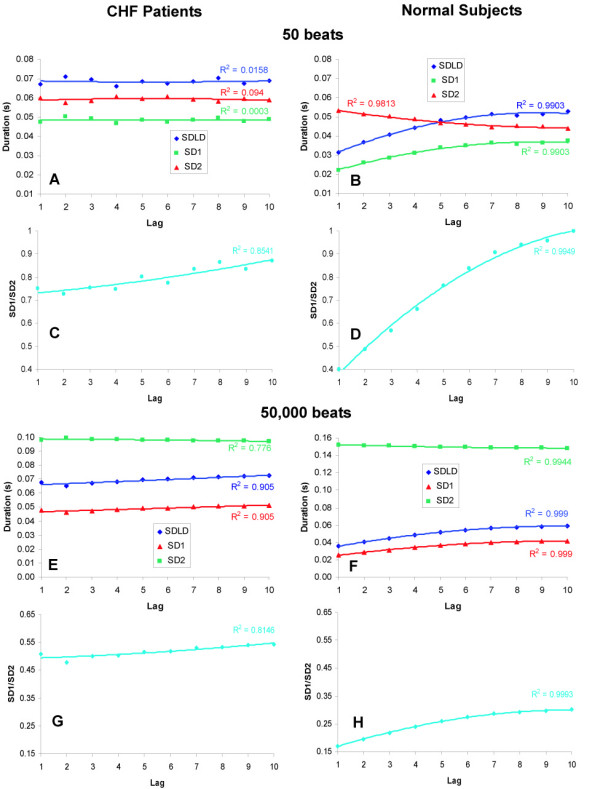
**Lag response of Poincaré plot indices in patients of CHF and normal subjects**. Panels A-D show the lag responses for sequences 50 beats long whereas panels E-H show the lag responses for sequences 50000 beats long. All the panels on the left hand side relate to subjects with CHF whereas the panels on the right hand side relate to normal subjects. We first estimated the Poincaré plot indices for each subject in both groups. We, then, estimated the averages for each group and plotted the estimates against the lag. In the normal subjects, all the Poincaré plot indices showed a curvilinear association with lag. In patients of CHF the curvilinearity was lost.

We analyzed the association between Poincaré plot-related estimates of heart rate variability and lag for varying beat sequence lengths. Figure 3 shows these associations for two extremes of beat sequence lengths (50, Figures 3A–3D; 50000, Figures 3E–3H). We observed that SDLD, SD1, SD2, and SD1/SD2 ratio were lag-invariant (R^2 ^range 0.0003–0.094) in CHF patients using the smallest sequence length of 50 beats. In striking contrast, smooth second-order polynomial curves explained more than 99 percent of the variance in the estimates of these indices on the basis of lag in the normal subjects. We therefore compared the mean values of coefficients of the quadratic term in CHF patients versus that in normal subjects. It can be observed from Table 2 that the coefficient of the quadratic term was consistently insignificant in CHF patients for all the indices studied. In normal subjects, however, all the indices had significant curvilinearity as indicated by significant coefficients of the quadratic term (Table 2). These results indicated that the curvilinearity of the lag-response is diminished in CHF. This seminal finding was replicated at the other extreme of sequence length of 50000 beats (Figures 3E–3H, Table 2). In fact, we observed the same finding regardless of sequence lengths.

**Table 2:** Coefficients and statistical significance of the quadratic term in equations regressing heart rate variability indices on lag.

HRV index	CHF patients		Normal subjects	
	
	Coefficient	P	Coefficient	P
Beat sequence length = 50				

SDLD	0.00002	0.769	-0.00033	3.5 × 10^-5^
SD1	0.00002	0.741	-0.00020	3 × 10^-5^
SD2	-0.00004	0.417	0.00010	0.002
SD1/SD2	0.00062	0.553	-0.00510	0.0003

Beat sequence length = 50000				

SDLD	1.5 × 10^-6^	0.968	-0.00028	1 × 10^-7^
SD1	2.27 × 10^-6^	0.932	-0.00020	1.4 × 10^-7^
SD2	-0.00001	0.502	0.00003	0.078
SD1/SD2	0.00028	0.531	-0.00158	4 × 10^-8^

We again examined if the increased HRV seen in the CHF patients (described in the previous section) confounded our interpretations of the lagged Poincaré plot analyses. We observed that (Supplementary Table 1 in additional file 1) 10 of the 29 CHF patients and 8 of the 54 control subjects had pNN50 values exceeding 10%. In addition 7 of the control subjects had mean heart rates over 100. To exclude the possibility that these abnormal values for pNN50 and mean heart rates could be responsible for the higher HRV in CHF as against normal subjects, we excluded these 25 subjects and performed a subset analysis on 19 CHF patients and 39 control subjects. The results from these subset analyses are detailed in supplementary tables 2 and 3 (see additional file 1). These results obtained are completely concordant with those obtained without excluding any subject from analysis (Tables 1 and 2). Thus, high pNN50 and mean heart rates in some subjects did not seem to influence our main inference about the loss of a curvilinear lag response in CHF.

## Discussion

Our results proffer compelling evidence that normally there is a curvilinear relation between lag and Poincaré plot indices of heart rate variability and that this curvilinearity is impaired in CHF. The main implication of our work is that differences in heart rate variability of CHF patients and normal subjects can be detected when these are not apparent from traditional measures of HRV. In addition, a potential interpretation of these results may be that the short term influence of the lagged beats is importantly determined by the respiratory sinus arrhythmia. Although traditionally viewed as an autonomic-mediated phenomenon, respiratory sinus arrhythmia may also involve non-autonomic mechanisms. The presence of complex Poincaré plots in the CHF group suggests that a combination of autonomic and non-autonomic control mechanisms may be responsible for the sinus arrhythmia in some of these patients. Further investigation is needed to better define the interpretation of these analyses in CHF patients.

Traditionally, HRV analysis has been used to assess the autonomic status of the heart and Poincaré plots have been widely used for HRV analysis. But the potential utility of lagged Poincaré plots has not been fully appreciated. Considering the fact that a heart beat is affected by its preceding beats, it follows logically that lag would affect the indices of HRV. We expect this effect to be stronger at the lower values of lag and to become weaker with increasing lag. Therefore, we expect a curvilinear relation between lag and indices of HRV normally. This curvilinearity is lost in CHF. This analysis, then, uncovers a potential means to further risk stratify patients with CHF.

### Useful length of beat sequence

Our study also demonstrated that a sequence of 5000 beats and more is useful for analyzing both the short-term and long-term HRV. Although our study was not designed to identify the useful minimum length of a beat sequence, we did observe the association of the estimates of Poincaré plot indices with varying lengths of beat sequences. We also found that the SD1/SD2 ratio was significantly different in CHF patients as compared to normal subjects for any length of the beat sequences. This observation is consistent with the recent notion that SDSD, SD1, and SD2 only capture the linear aspect of HRV whereas the SD1/SD2 ratio may better relate to the non-linear component of HRV [2,16]. Therefore, in the time-domain analysis of ECG, it may be the most informative to use the SD1/SD2 ratio. Our method further capitalizes on this property of the SD1/SD2 ratio by using m-lagged SD1/SD2 ratios. Such a modification, by virtue of an implicit property of the autocovariance function, can improve the use of SD1/SD2 ratio as a result of a closer approximation of frequency-domain analysis.

### Differences in HRV in CHF and in normal sinus rhythm

The analysis conducted in this study makes use of the autocovariance function which allows for a stronger and more robust internal comparison for individual subjects as opposed to the more inaccurate method of group comparisons. We observed a loss of curvilinearity of lag-response that prevailed in CHF patients with therapy in spite of an improvement in HRV attributable to the drug therapy. It is known that digoxin only improves the clinical profile of CHF patients but does little to improve survival [21,23]. Thus, even though the patients on therapy seem to have greater HRV than normal, they might still be at risk for early death. This can have great prognostic implications. We propose that patients in whom lag-response curvilinearity is restored may have a better prognosis than those in whom it is not restored. Further prospective studies are needed to test this hypothesis. Nevertheless, these initial findings suggest that this method offers a unique advantage over the conventional method of 1-lagged Poincaré plots.

### Study limitations

Our study suffers from several limitations. First, we conducted a secondary data analysis. In addition to the inherent limitations of such analyses, we were restricted to the analysis of the ECG signals only. It would have been more informative to study other patho-physiological and socio-demographic correlates of CHF. Second, the small sample sizes of both the CHF and normal sinus rhythm datasets do not allow wider generalization of our results. Therefore, studies of larger magnitude that can provide more conclusive insights into the utility of these measures of HRV are needed. Third, rather surprisingly, we observed more heart rate variability in CHF patients as compared to the normal subjects, which contrasts the generally accepted notion that HRV is decreased in CHF as compared to normal sinus rhythm. This unexpected result can be partially explained by the facts that i) A large proportion of the CHF patients exhibited patterns of complex Poincaré plots; ii) It is possible that the differential distribution of age, gender, socioeconomic status and other unknown parameters in the comparison group may have confounded the observations on increased heart rate variability in CHF subjects; and iii) all the CHF patients in this study were receiving long-term digoxin therapy [19,20] which can potentially improve HRV in the CHF patients [23,24].

Furthermore, though SD1 and SD1/SD2 ratios were higher in CHF patients as compared to normal subjects, SD2 (an index of long-term variability) was higher in the normal subjects for beat sequences 500 and above. This is consistent with two sets of observations: first, CHF patients are more likely to have a high degree of sinus arrhythmia that is not of respiratory origin, that is, they are more likely to have sudden jumps in the N-N interval; and second, a study by Huikuri *et al *reported that depression of long term variability is a predictor of mortality [25]. Thus, CHF patients in this study had lower SD2 and hence a lower long-term heart rate variability than normal subjects, which might be responsible for the poorer prognosis in the former. We also studied three other measures of HRV across the study groups: percentage of beats with change in successive NN intervals exceeding 50 ms (pNN50) and 20 ms (pNN20) as well as the standard deviation of the NN intervals (SDNN). The individual data is shown in Supplementary Table 1 (see additional file 1). Using a Mann-Whitney test we observed that while in general the values for all the three parameters tended to be higher in the CHF group, these were not statistically significantly different (Supplementary Table 4, see additional file 1). These observations further highlight the value of the proposed method to gain insights into HRV.

## Conclusion

In summary, despite the presence of these limitations, our study provides strong evidence for the loss of curvilinearity in the lag-response of Poincaré plot indices in CHF patients even after receiving long-term therapy and even in cases in which some traditional measures of HRV are similar to those seen in normal individuals. We propose that these approaches of analysis can be used to improve the time-domain analysis of ECG signals and can enhance the diagnostic and prognostic indicators of CHF.

## Abbreviations

**1. **CHF – Congestive Heart Failure

**2. **ECG – Electrocardiogram

**3. **HRV – Heart Rate Variability

**4. **RR – RR interval (these are considered to be measured from normal beats, N-N)

**5. **SD1 – Standard Deviation 1 (long axis of ellipse fitted to Poincaré plot)

**6. **SD2 – Standard Deviation 2 (short axis of ellipse fitted to Poincaré plot)

**7. **SDLD – Standard Deviation of Lagged Deltas

**8. **SDRR – Standard Deviation of RR intervals

**9. **SDSD – Standard Deviation of Successive Differences

**10. **pNN50 – percentage of beats with change in successive NN intervals exceeding 50 ms

**11. **pNN20 – percentage of beats with change in successive NN intervals exceeding 20 ms

**12. **SDNN – standard deviation of the NN intervals

## Competing interests

The author(s) declare that they have no competing interests.

## Authors' contributions

TPT conceptualized the study, performed the analysis of the data and wrote the initial and revised drafts of the manuscript. MLS contributed to the data analysis and the review and revision of the manuscript. All authors read and approved the final manuscript.

## Pre-publication history

The pre-publication history for this paper can be accessed here:



## Supplementary Material

Additional file 1 This document contains supplementary tables 1–4. Supplementary Table 1 details characteristics of the subjects included in the present analysis. Supplementary Table 2 shows replication of analyses shown in Table 1 (Main Text) in the subset of subjects who had pNN50 ≤ 10 (for both CHF and NSR datasets) and mean heart rate <100 beats/min (for the NSR dataset). Supplementary Table 3 shows replication of analyses shown in Table 2 (Main Text) in the subset of subjects who had pNN50 ≥ 10 (for both CHF and NSR datasets) and mean heart rate <100 beats/min (for the NSR dataset). Supplementary Table 4 shows the distribution of traditional measures of HRV across the study groups. Click here for file

Additional file 2 This file has hourly Poincaré plots for the 29 CHF patients. Click here for file

Additional file 3 This file has hourly Poincaré plots for the 54 control subjects. Click here for file
