# Supplementary material for: Loss of lag-response curvilinearity of indices of heart rate variability in congestive heart failure
Source: BMC Cardiovasc Disord. 2006 Jun 12;6:27. doi: 10.1186/1471-2261-6-27 (PMC1523370; doi:10.1186/1471-2261-6-27)
Supplement: Additional file 1 — This document contains supplementary tables 1–4. Supplementary Table 1 details characteristics of the subjects included in the present analysis. Supplementary Table 2 shows replication of analyses shown in Table 1 (Main Text) in the subset of subjects who had pNN50 ≤ 10 (for both CHF and NSR datasets) and mean heart rate <100 beats/min (for the NSR dataset). Supplementary Table 3 shows replication of analyses shown in Table 2 (Main Text) in the subset of subjects who had pNN50 ≥ 10 (for both CHF and NSR datasets) and mean heart rate <100 beats/min (for the NSR dataset). Supplementary Table 4 shows the distribution of traditional measures of HRV across the study groups. [file 1471-2261-6-27-S1.doc]

**Supplementary Table 1.** Characteristics of the subjects included in the present analysis. pNN50 and pNN20 are expressed as percentage. All measures of HRV are for beat sequence length of 5000 beats.

| **No** | **PhysioNet Identifier** | **Age** | **Gender** | **NYHA class** | **pNN50** | **pNN20** | **SDNN (ms)** | **Mean Heart Rate** |
| --- | --- | --- | --- | --- | --- | --- | --- | --- |
| **Congestive Heart Failure Database** | | | | | | | | |
| 1 | chf201 | 55 | M | III | 5.25 | 39.31 | 98.5 | 79 |
| 2 | chf202 | 59 | F | III | 1.98 | 33.73 | 68.1 | 96 |
| 3 | chf203 | 68 | M | III | 2.24 | 5.42 | 739.8 | 74 |
| 4 | chf204 | 62 | M | III | 4.99 | 28.50 | 186 | 83 |
| 5 | chf205 | 39 | M | III | 7.98 | 26.87 | 920.3 | 93 |
| 6 | chf206 | 38 | F | III | 2.29 | 4.58 | 23.2 | 109 |
| 7 | chf207 | 62 | M | III | 33.04 | 35.98 | 1462.6 | 88 |
| 8 | chf208 | 62 | M | III | 11.47 | 29.62 | 158 | 79 |
| 9 | chf209 | 65 | M | III | 0.73 | 4.12 | 159.2 | 93 |
| 10 | chf210 | 43 | M | III | 2.06 | 9.13 | 102.1 | 103 |
| 11 | chf211 | 34 | ? | II | 2.72 | 26.45 | 210.5 | 94 |
| 12 | chf212 | 54 | ? | II | 13.00 | 30.26 | 231.2 | 107 |
| 13 | chf213 | 53 | ? | I | 27.56 | 51.86 | 166.1 | 77 |
| 14 | chf214 | 79 | ? | II | 28.62 | 46.84 | 1084.2 | 98 |
| 15 | chf215 | 43 | ? | II | 0.58 | 8.00 | 352.9 | 112 |
| 16 | chf216 | 58 | ? | II | 0.28 | 7.82 | 38.8 | 82 |
| 17 | chf217 | 50 | ? | I | 1.73 | 13.73 | 254.5 | 98 |
| 18 | chf218 | 72 | ? | I | 18.07 | 32.90 | 451.2 | 93 |
| 19 | chf219 | 62 | ? | III | 0.70 | 17.88 | 57.4 | 83 |
| 20 | chf220 | 64 | ? | II | 2.02 | 9.54 | 450.9 | 91 |
| 21 | chf221 | 37 | ? | I | 39.78 | 54.75 | 439.9 | 99 |
| 22 | chf222 | 63 | ? | III | 16.46 | 25.51 | 2081.4 | 64 |
| 23 | chf223 | 56 | ? | III | 18.96 | 38.16 | 3774.5 | 64 |
| 24 | chf224 | 35 | ? | II | 0.63 | 5.61 | 41.1 | 93 |
| 25 | chf225 | 66 | ? | III | 6.30 | 15.67 | 1308.3 | 79 |
| 26 | chf226 | 51 | ? | II | 1.18 | 1.62 | 24.1 | 91 |
| 27 | chf227 | 64 | ? | III | 15.75 | 21.84 | 452.1 | 79 |
| 28 | chf228 | 51 | ? | III | 8.83 | 22.80 | 270.6 | 89 |
| 29 | chf229 | 58 | ? | III | 3.54 | 38.19 | 448.2 | 89 |
| **Normal Sinus Rhythm Database** | | | | | | | | |
| 30 | nsr001 | 64 | F |  | 0.40 | 14.04 | 72.6 | 101 |
| 31 | nsr002 | 67 | M |  | 1.20 | 22.61 | 71 | 78 |
| 32 | nsr003 | 67 | F |  | 12.56 | 42.84 | 202 | 76 |
| 33 | nsr004 | 62 | F |  | 5.92 | 40.10 | 110.9 | 74 |
| 34 | nsr005 | 62 | F |  | 6.66 | 29.69 | 102.5 | 98 |
| 35 | nsr006 | 64 | M |  | 2.92 | 31.52 | 119.9 | 83 |
| 36 | nsr007 | 76 | M |  | 1.13 | 25.00 | 171.5 | 84 |
| 37 | nsr008 | 64 | F |  | 1.47 | 24.20 | 108.3 | 81 |
| 38 | nsr009 | 66 | M |  | 6.30 | 31.66 | 106.7 | 78 |
| 39 | nsr010 | 61 | F |  | 8.46 | 45.50 | 114.4 | 70 |
| 40 | nsr011 | 65 | F |  | 4.79 | 39.37 | 56.4 | 78 |
| 41 | nsr012 | 66 | M |  | 0.54 | 11.06 | 132.8 | 90 |
| 42 | nsr013 | 63 | F |  | 1.70 | 17.43 | 105.6 | 112 |
| 43 | nsr014 | 65 | F |  | 17.25 | 41.24 | 85 | 78 |
| 44 | nsr015 | 74 | M |  | 3.20 | 28.67 | 333 | 79 |
| 45 | nsr016 | 73 | F |  | 3.42 | 25.60 | 1157.6 | 71 |
| 46 | nsr017 | 71 | F |  | 24.74 | 59.94 | 123.4 | 69 |
| 47 | nsr018 | 68 | M |  | 5.35 | 36.49 | 146.3 | 75 |
| 48 | nsr019 | 65 | F |  | 0.26 | 3.71 | 50.8 | 113 |
| 49 | nsr020 | 58 | F |  | 3.05 | 29.20 | 111.3 | 102 |
| 50 | nsr021 | 59 | M |  | 2.85 | 37.17 | 59.6 | 82 |
| 51 | nsr022 | 68 | M |  | 17.15 | 56.13 | 125.3 | 60 |
| 52 | nsr023 | 66 | F |  | 0.21 | 6.83 | 58.7 | 89 |
| 53 | nsr024 | 63 | F |  | 35.88 | 54.65 | 119.9 | 81 |
| 54 | nsr025 | 75 | M |  | 3.05 | 35.21 | 232.9 | 91 |
| 55 | nsr026 | 72 | M |  | 0.51 | 13.58 | 205.9 | 92 |
| 56 | nsr027 | 64 | M |  | 1.64 | 7.75 | 2244.9 | 92 |
| 57 | nsr028 | 65 | M |  | 1.97 | 12.56 | 235 | 91 |
| 58 | nsr029 | 63 | M |  | 1.06 | 13.12 | 1292.9 | 96 |
| 59 | nsr030 | 70 | F |  | 0.47 | 16.42 | 257.1 | 89 |
| 60 | nsr031 | 67 | M |  | 4.02 | 20.85 | 347.2 | 80 |
| 61 | nsr032 | 68 | M |  | 5.67 | 9.34 | 4572.5 | 71 |
| 62 | nsr033 | 65 | M |  | 5.42 | 37.68 | 601.5 | 60 |
| 63 | nsr034 | 67 | M |  | 5.33 | 32.70 | 922.6 | 59 |
| 64 | nsr035 | 66 | M |  | 0.71 | 20.00 | 173.6 | 89 |
| 65 | nsr036 | 60 | F |  | 2.69 | 25.29 | 492.2 | 96 |
| 66 | nsr037 | 63 | M |  | 0.82 | 18.29 | 150.8 | 75 |
| 67 | nsr038 | 62 | M |  | 0.92 | 10.02 | 441.7 | 99 |
| 68 | nsr039 | 70 | F |  | 2.77 | 19.49 | 997.2 | 79 |
| 69 | nsr040 | 63 | F |  | 1.95 | 9.87 | 199 | 87 |
| 70 | nsr041 | 64 | F |  | 7.29 | 46.56 | 1610.8 | 64 |
| 71 | nsr042 | 68 | F |  | 1.77 | 21.26 | 299.9 | 87 |
| 72 | nsr043 | 66 | M |  | 2.09 | 14.76 | 913.6 | 84 |
| 73 | nsr044 | 65 | F |  | 8.90 | 25.30 | 499.9 | 96 |
| 74 | nsr045 | 67 | F |  | 12.28 | 47.72 | 1236.4 | 82 |
| 75 | nsr046 | 63 | F |  | 8.47 | 27.91 | 164.2 | 85 |
| 76 | nsr047 | 28.5 | M |  | 2.02 | 27.53 | 248.5 | 88 |
| 77 | nsr048 | 38 | M |  | 3.96 | 35.63 | 414.4 | 85 |
| 78 | nsr049 | 39 | M |  | 11.92 | 47.90 | 224.1 | 72 |
| 79 | nsr050 | 29 | M |  | 1.73 | 21.07 | 86.6 | 107 |
| 80 | nsr051 | 40 | M |  | 22.57 | 60.40 | 451.9 | 76 |
| 81 | nsr052 | 39 | M |  | 0.53 | 14.99 | 81.4 | 103 |
| 82 | nsr053 | 35 | M |  | 9.41 | 48.65 | 98.1 | 76 |
| 83 | nsr054 | 35 | M |  | 2.30 | 20.96 | 227.5 | 101 |

**Supplementary Table 2.** Replication of analyses shown in Table 1 (Main Text) in the subset of subjects who had pNN50 ≤10 (for both CHF and NSR datasets) and mean heart rate <100 beats/min (for the NSR dataset).

| Beat sequence length | SD1 | | | SD2 | | | SD1/SD2 ratio | | |
| --- | --- | --- | --- | --- | --- | --- | --- | --- | --- |
|  | CHF | Normal | p | CHF | Normal | p | CHF | Normal | p |
| 50 | 0.0413  (0.0688) | 0.0206  (0.0303) | 0.9077 | 0.0512  (0.0610) | 0.0521  (0.0336) | 0.1381 | 0.7134  (0.3797) | 0.3775  (0.2117) | 0.0003 |
| 100 | 0.0381  (0.0655) | 0.0197  (0.0228) | 0.8880 | 0.0554  (0.0653) | 0.0578  (0.0273) | 0.0424 | 0.5835  (0.2779) | 0.3454  (0.2311) | 0.0005 |
| 500 | 0.0248  (0.0269) | 0.0204  (0.0121) | 0.6250 | 0.0548  (0.0381) | 0.0798  (0.0366) | 0.0227 | 0.5175  (0.2920) | 0.2798  (0.1603) | 0.0029 |
| 1000 | 0.0243  (0.0264) | 0.0196  (0.0096) | 0.5676 | 0.0589  (0.0410) | 0.0898  (0.0355) | 0.0040 | 0.4759  (0.2791) | 0.2340  (0.1193) | 0.0007 |
| 5000 | 0.0262  (0.0230) | 0.0186  (0.0084) | 0.3083 | 0.0620  (0.0345) | 0.1068  (0.0332) | 1 x 10^-5^ | 0.4518  (0.2184) | 0.1866  (0.0959) | 2 x 10^-6^ |
| 10000 | 0.0302  (0.0254) | 0.0184  (0.0069) | 0.1174 | 0.0668  (0.0337) | 0.1122  (0.0324) | 8 x 10^-5^ | 0.4400  (0.1938) | 0.1733  (0.0753) | 1 x 10^-7^ |
| 50000 | 0.0310  (0.0214) | 0.0208  (0.0090) | 0.0516 | 0.0815  (0.0418) | 0.1551  (0.0477) | 2 x 10^-6^ | 0.4175  (0.2159) | 0.1437  (0.0674) | 2 x 10^-7^ |
| Spearman’s rho | 0.1637 | 0.1986 | 0.8181 | 0.3299 | 0.6887 | 2 x 10^-6^ | -0.2701 | -0.5597 | 0.0009 |

**Supplementary Table 3.** Replication of analyses shown in Table 2 (Main Text) in the subset of subjects who had pNN50 ≤10 (for both CHF and NSR datasets) and mean heart rate <100 beats/min (for the NSR dataset).

| HRV index | CHF patients | | Normal subjects | |
| --- | --- | --- | --- | --- |
|  | Coefficient | p | Coefficient | p |
| Beat sequence length = 50 | | | | |
| SDLD | 0.00001 | 0.862 | -0.00042 | 0.0004 |
| SD1 | -0.00005 | 0.938 | -0.00024 | 0.0008 |
| SD2 | 2.3x10^-6^ | 0.997 | 0.00010 | 0.006 |
| SD1/SD2 | -0.00139 | 0.706 | -0.00560 | 0.0011 |
| Beat sequence length = 50000 | | | | |
| SDLD | 2.8 x 10^-7^ | 0.991 | -0.00034 | 6 x 10^-6^ |
| SD1 | -0.00002 | 0.938 | -0.00020 | 0.008 |
| SD2 | 1.2x10^-6^ | 0.998 | 0.00003 | 0.092 |
| SD1/SD2 | 0.00004 | 0.985 | -0.00092 | 3 x 10^-6^ |

**Supplementary Table 4.** Distribution of traditional measures of HRV across the study groups for beat sequence length of 5000 beats.

| Measure of HRV | CHF  Mean (95% CI) | Normal  Mean (95% CI) | Mann-Whitney P |
| --- | --- | --- | --- |
| pNN50 (%) | 9.61 (5.44 – 13.8) | 5.59 (3.70 – 7.47) | 0.1859 |
| pNN20 (%) | 23.68 (17.91 – 29.44) | 28.10 (24.13 – 32.07) | 0.2363 |
| SDNN (s) | 0.55 (0.25 – 0.86) | 0.44 (0.24 – 0.64) | 0.5284 |
