# Supplementary material for: Loss of lag-response curvilinearity of indices of heart rate variability in congestive heart failure
Source: BMC Cardiovasc Disord. 2006 Jun 12;6:27. doi: 10.1186/1471-2261-6-27 (PMC1523370; doi:10.1186/1471-2261-6-27)
Supplement: Additional file 2 — This file has hourly Poincaré plots for the 29 CHF patients. [file 1471-2261-6-27-S2.pdf]

Hourly Poincaré Plots for CHF patients

This file contains the hourly Poincaré plots of all the CHF patients the data on whom was used in the study by Thakre and Smith. All the plots are plotted on a square representing 0.5 to 1.5 seconds on both axes. The x-axis represents the current beat while the y-axis represents the next beat. The identifier for the patient is in the box representing the hour 24 at the bottom of each page.

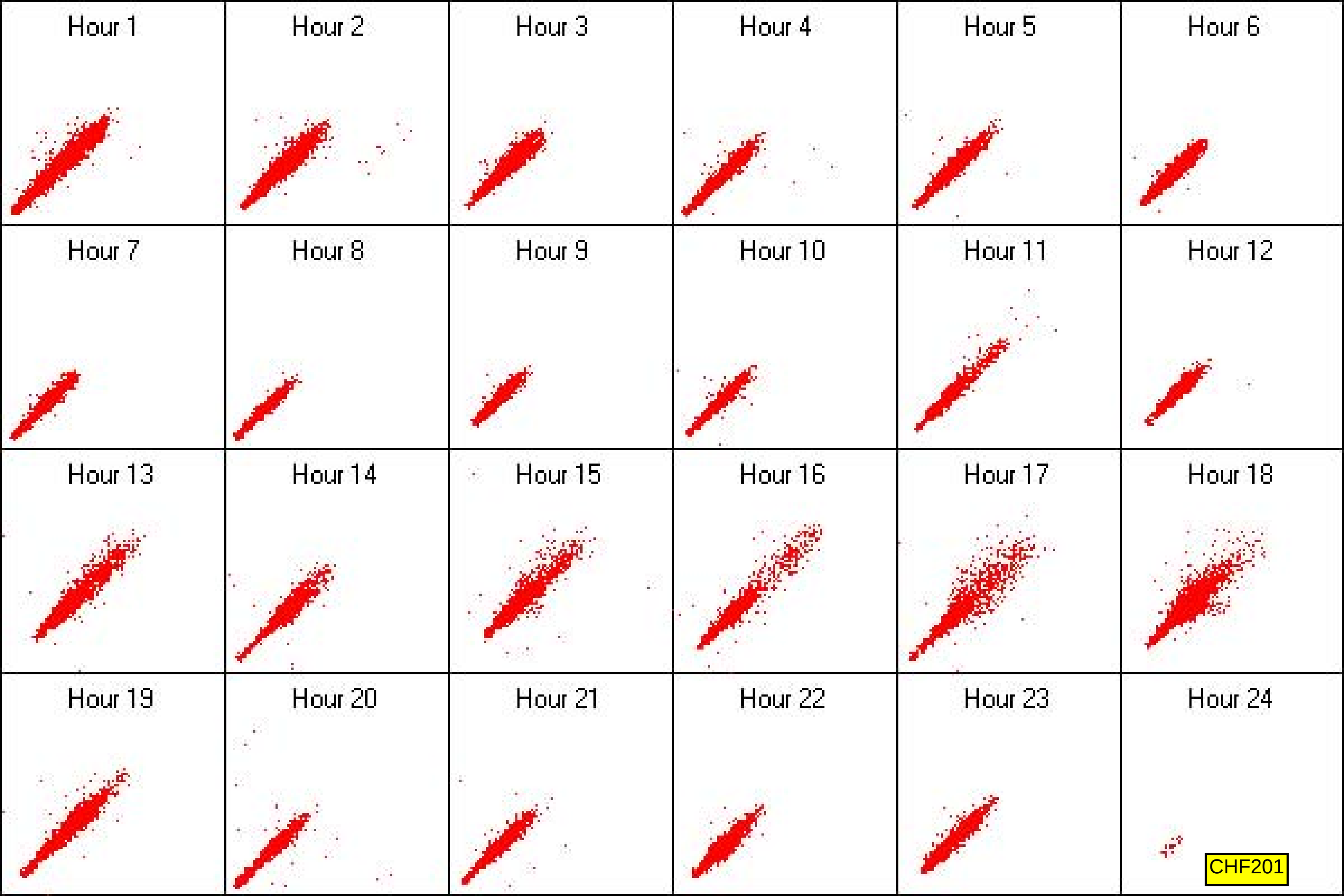
Hour 1
Hour 2
Hour 3
Hour 4
Hour 5
Hour 6
Hour 7
Hour 8
Hour 9
Hour 10
Hour 11
Hour 12
Hour 13
Hour 14
Hour 15
Hour 16
Hour 17
Hour 18
Hour 19
Hour 20
Hour 21
Hour 22
Hour 23
Hour 24
CHF201

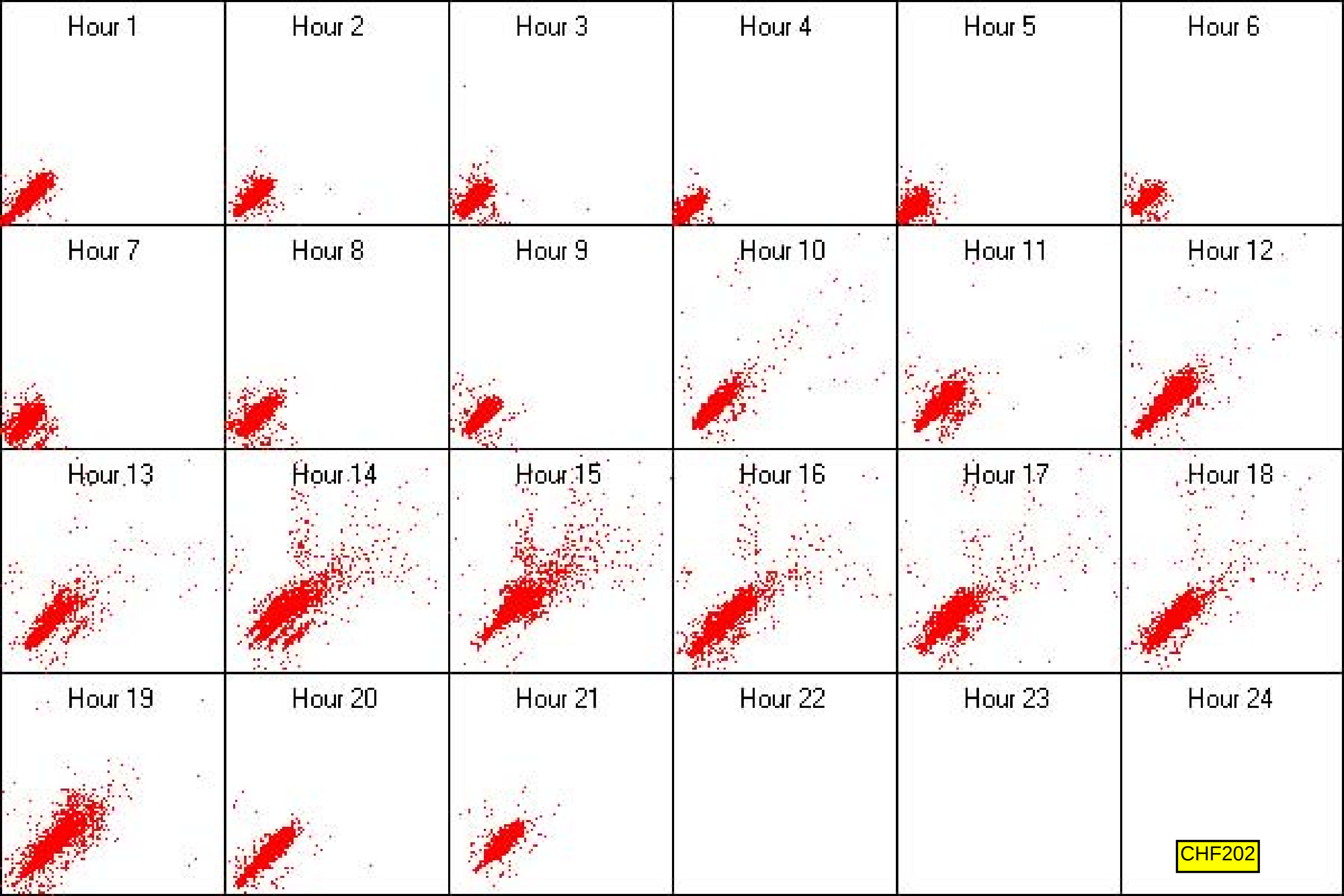

Hour 1
Hour 2
Hour 3
Hour 4
Hour 5
Hour 6
Hour 7
Hour 8
Hour 9
Hour 10
Hour 11
Hour 12
Hour 13
Hour 14
Hour 15
Hour 16
Hour 17
Hour 18
Hour 19
Hour 20
Hour 21
Hour 22
Hour 23
Hour 24
CHF202

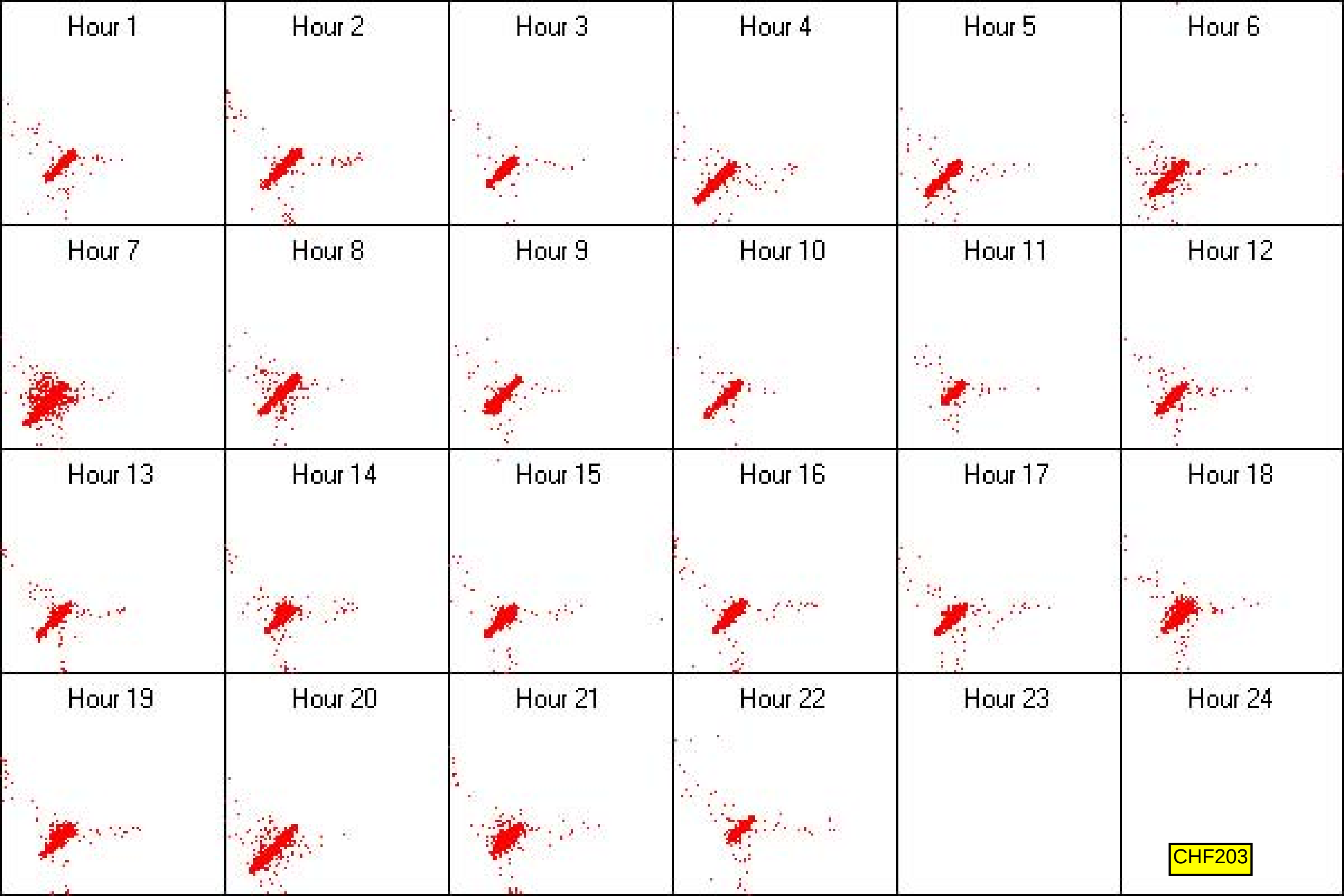
Hour 1
Hour 2
Hour 3
Hour 4
Hour 5
Hour 6
Hour 7
Hour 8
Hour 9
Hour 10
Hour 11
Hour 12
Hour 13
Hour 14
Hour 15
Hour 16
Hour 17
Hour 18
Hour 19
Hour 20
Hour 21
Hour 22
Hour 23
Hour 24
CHF203

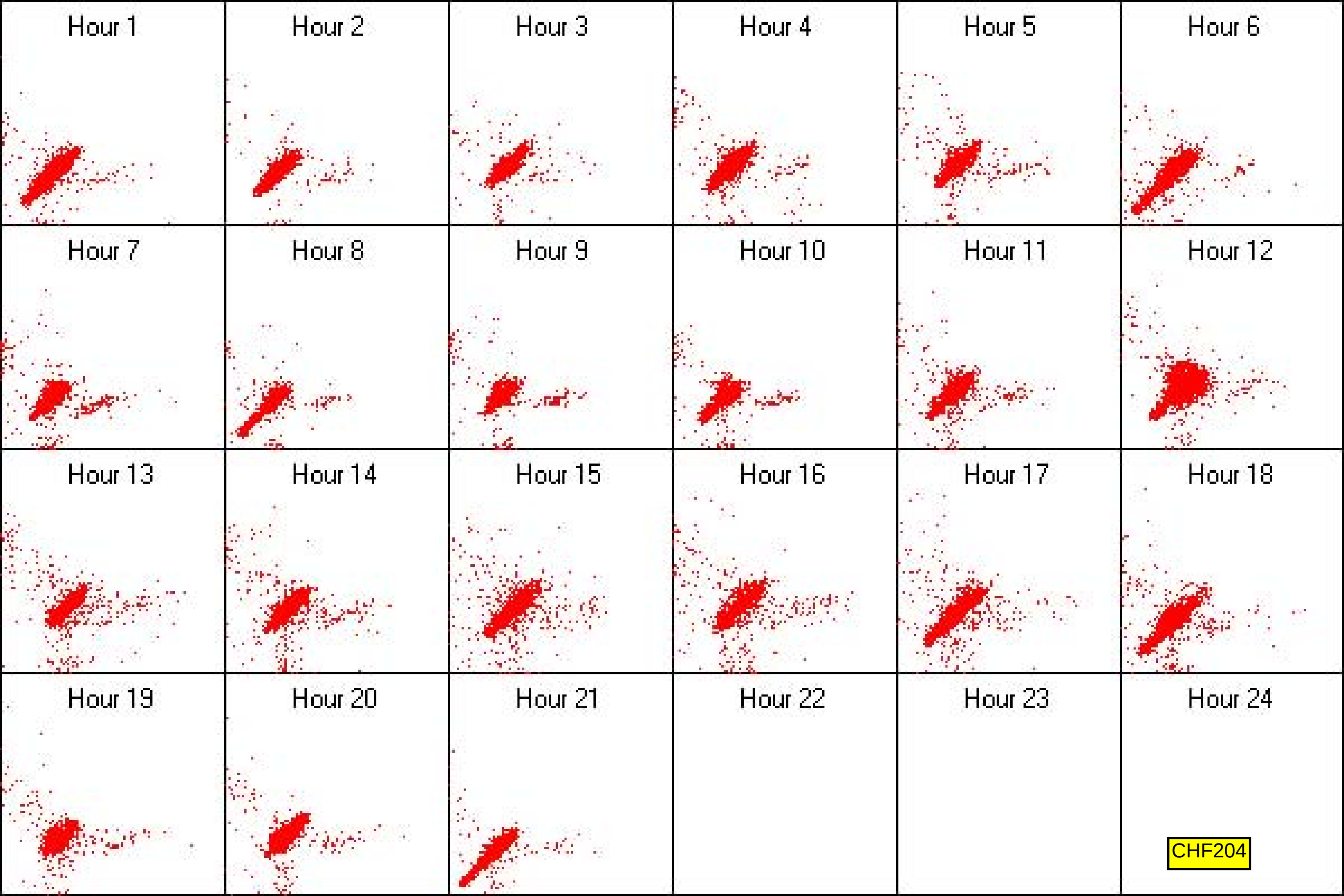
Hour 1
Hour 2
Hour 3
Hour 4
Hour 5
Hour 6
Hour 7
Hour 8
Hour 9
Hour 10
Hour 11
Hour 12
Hour 13
Hour 14
Hour 15
Hour 16
Hour 17
Hour 18
Hour 19
Hour 20
Hour 21
Hour 22
Hour 23
Hour 24
CHF204

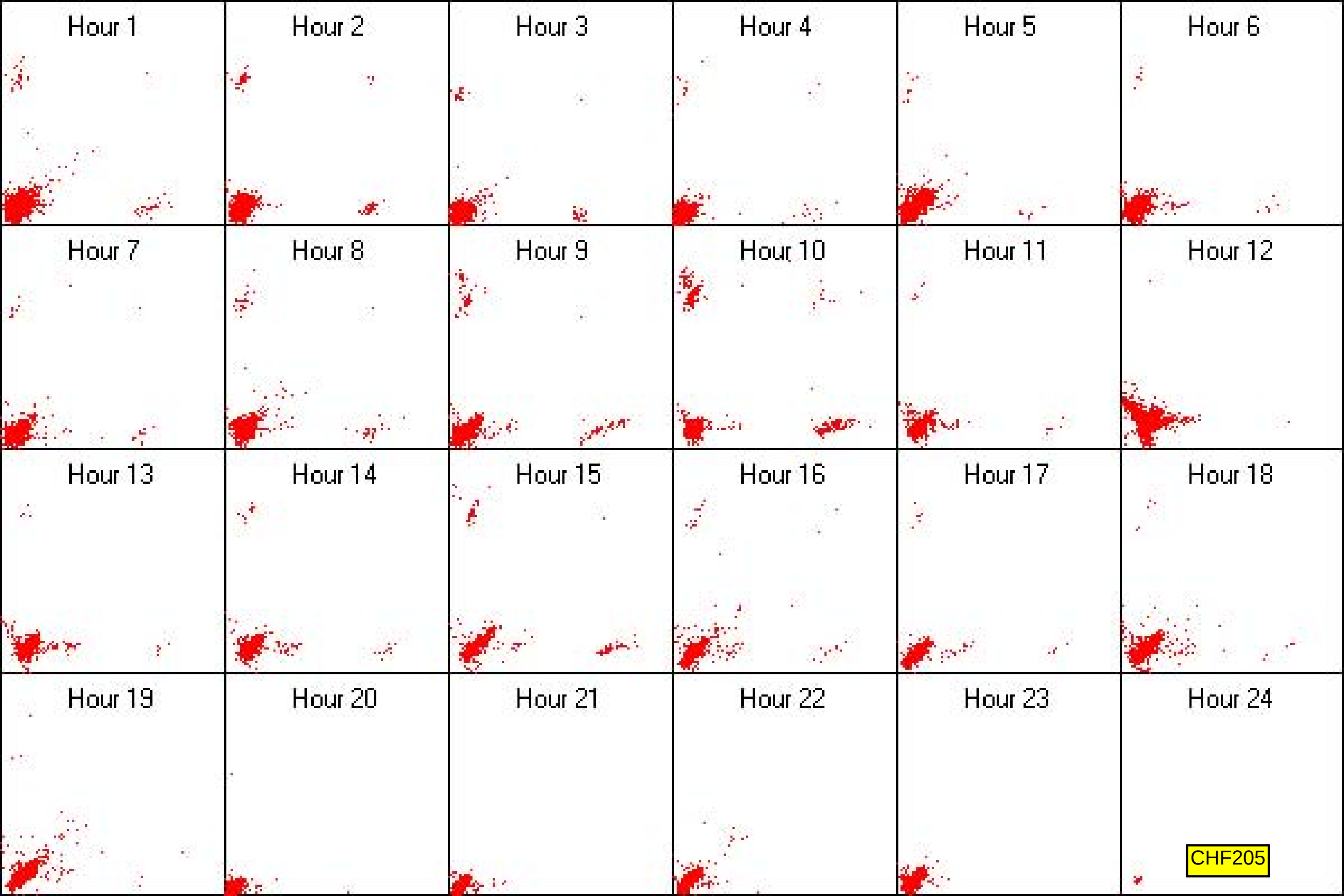
Hour 1
Hour 2
Hour 3
Hour 4
Hour 5
Hour 6
Hour 7
Hour 8
Hour 9
Hour 10
Hour 11
Hour 12
Hour 13
Hour 14
Hour 15
Hour 16
Hour 17
Hour 18
Hour 19
Hour 20
Hour 21
Hour 22
Hour 23
Hour 24
CHF205

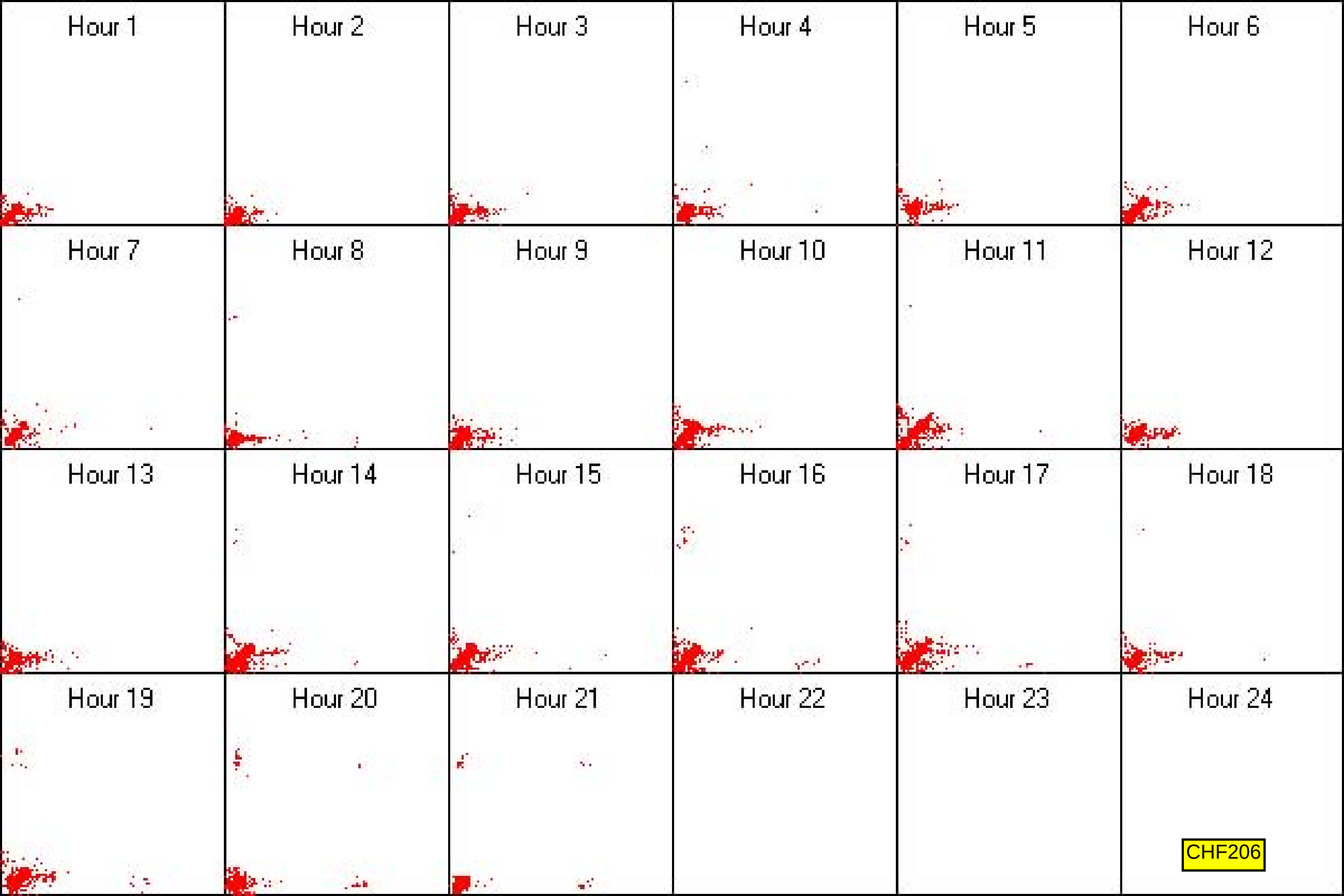
Hour 1
Hour 2
Hour 3
Hour 4
Hour 5
Hour 6
Hour 7
Hour 8
Hour 9
Hour 10
Hour 11
Hour 12
Hour 13
Hour 14
Hour 15
Hour 16
Hour 17
Hour 18
Hour 19
Hour 20
Hour 21
Hour 22
Hour 23
Hour 24
CHF206

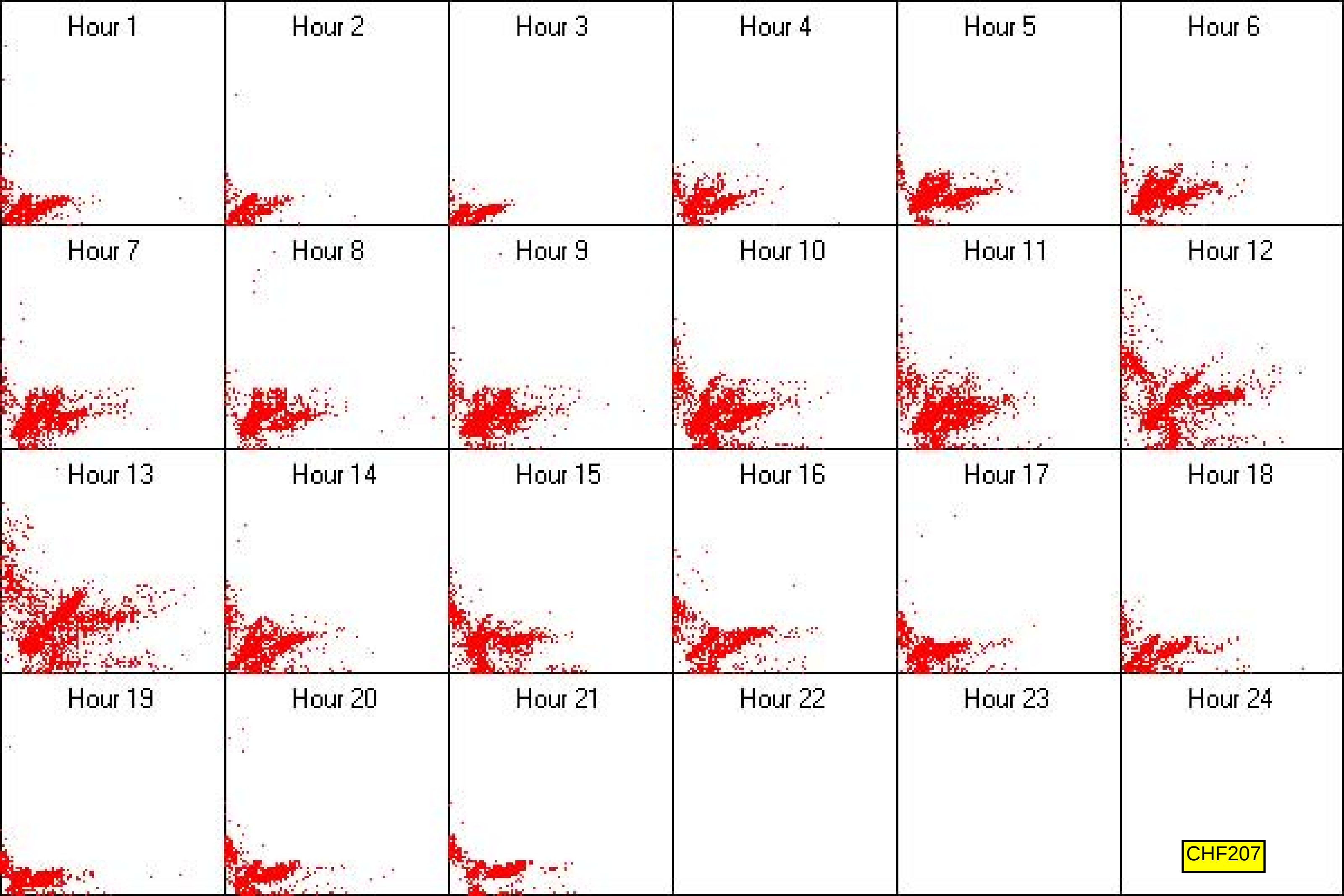
Hour 1
Hour 2
Hour 3
Hour 4
Hour 5
Hour 6
Hour 7
Hour 8
Hour 9
Hour 10
Hour 11
Hour 12
Hour 13
Hour 14
Hour 15
Hour 16
Hour 17
Hour 18
Hour 19
Hour 20
Hour 21
Hour 22
Hour 23
Hour 24
CHF207

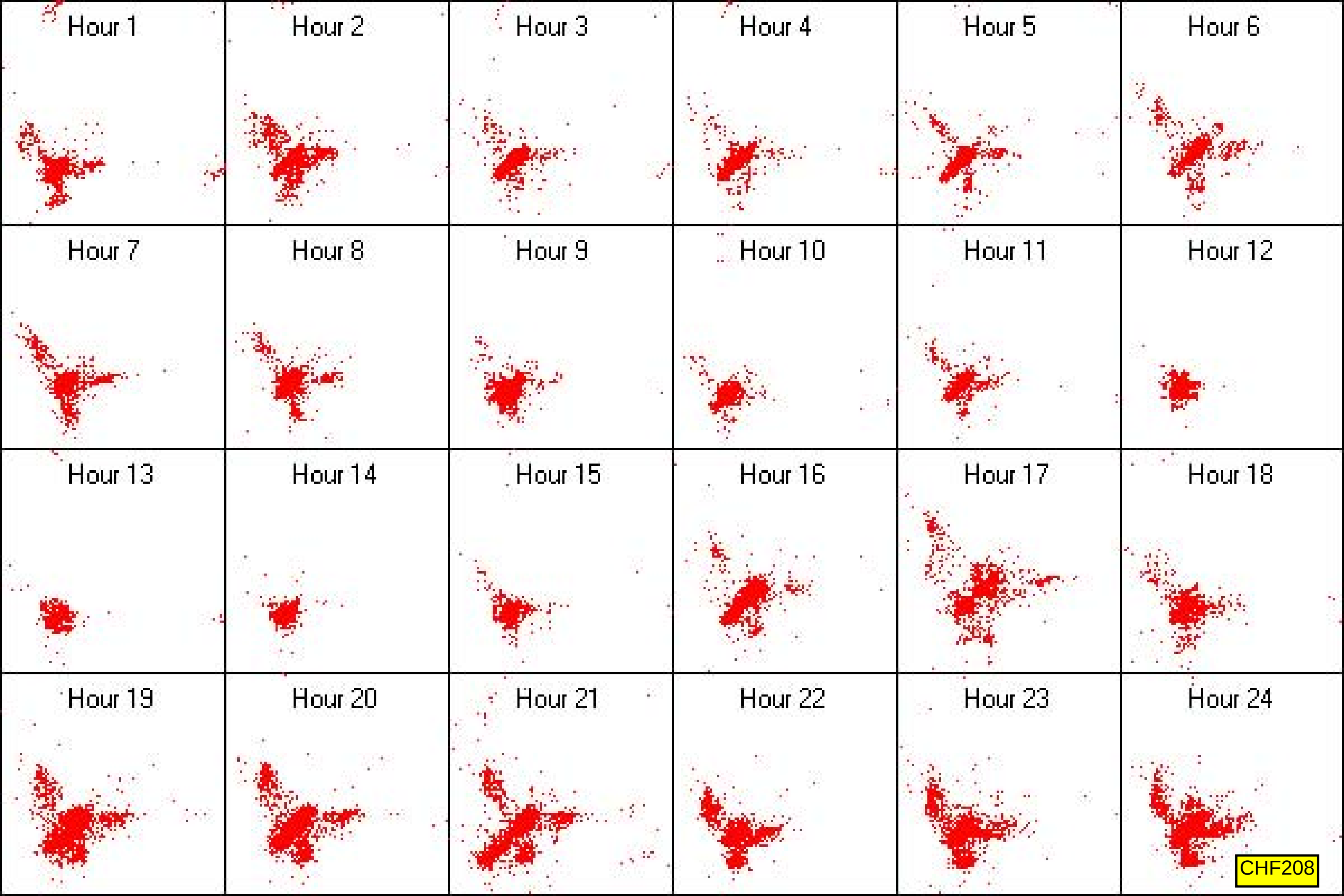

Hour 1
Hour 2
Hour 3
Hour 4
Hour 5
Hour 6
Hour 7
Hour 8
Hour 9
Hour 10
Hour 11
Hour 12
Hour 13
Hour 14
Hour 15
Hour 16
Hour 17
Hour 18
Hour 19
Hour 20
Hour 21
Hour 22
Hour 23
Hour 24
CHF208

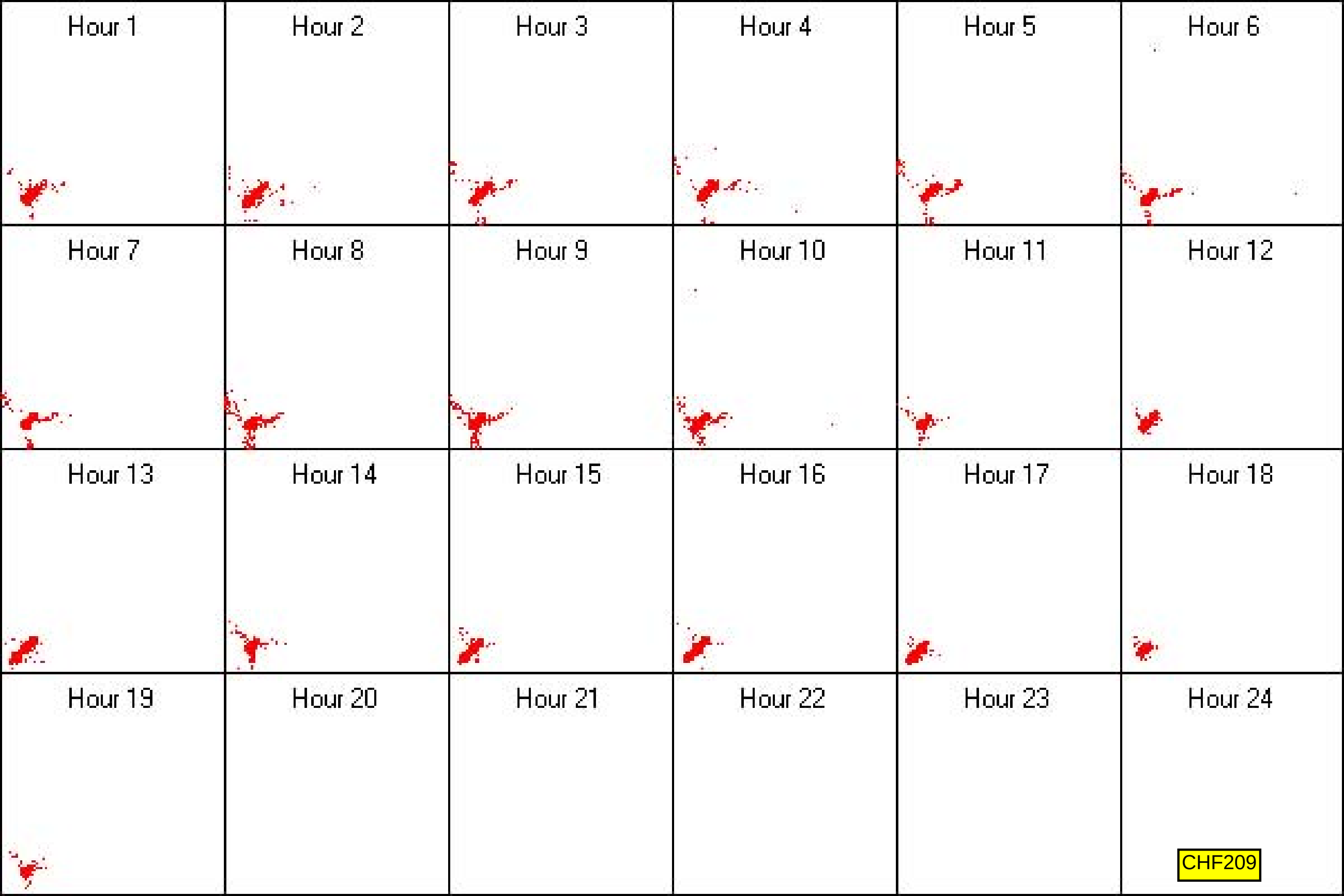
Hour 1
Hour 2
Hour 3
Hour 4
Hour 5
Hour 6
Hour 7
Hour 8
Hour 9
Hour 10
Hour 11
Hour 12
Hour 13
Hour 14
Hour 15
Hour 16
Hour 17
Hour 18
Hour 19
Hour 20
Hour 21
Hour 22
Hour 23
Hour 24
CHF209

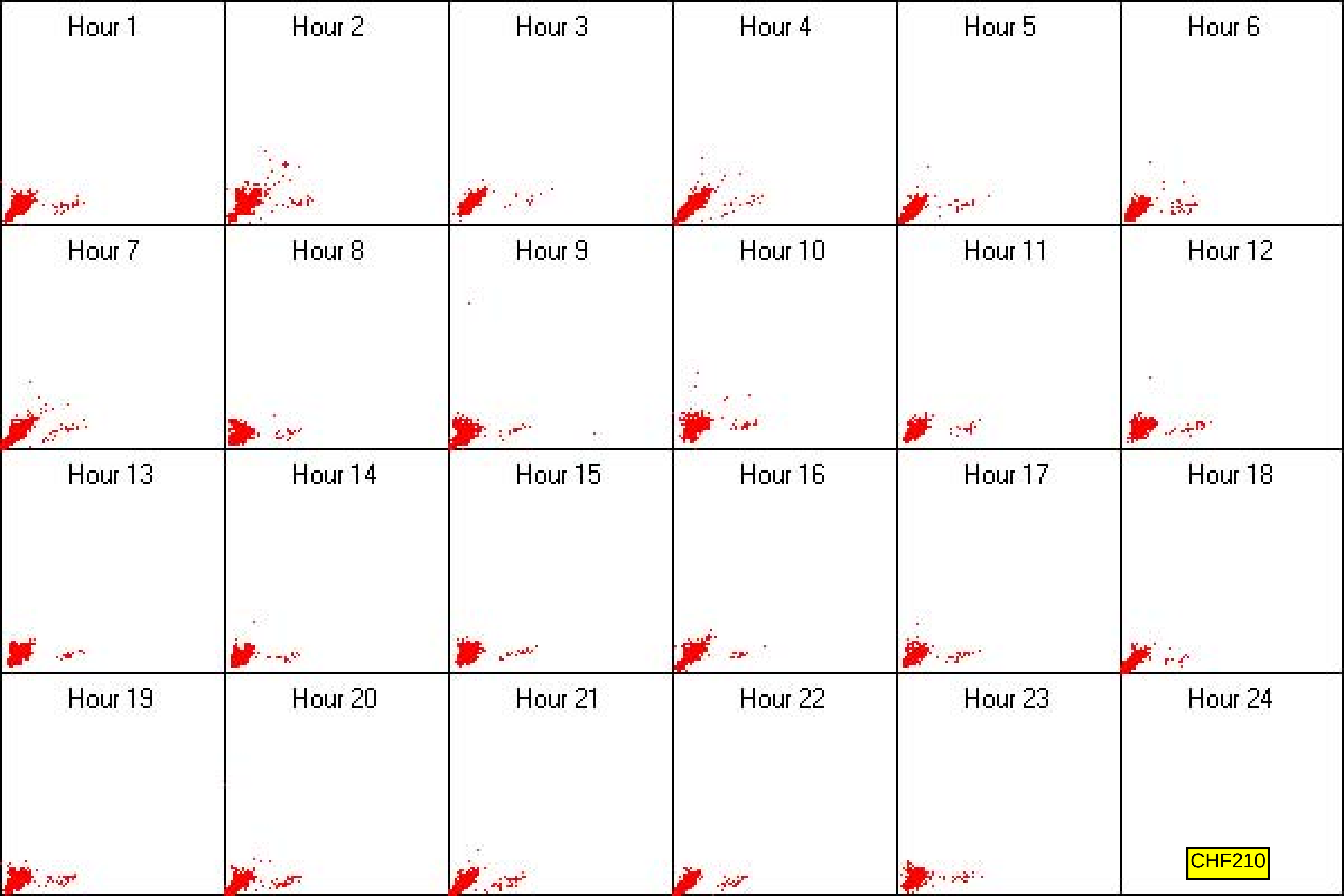
Hour 1
Hour 2
Hour 3
Hour 4
Hour 5
Hour 6
Hour 7
Hour 8
Hour 9
Hour 10
Hour 11
Hour 12
Hour 13
Hour 14
Hour 15
Hour 16
Hour 17
Hour 18
Hour 19
Hour 20
Hour 21
Hour 22
Hour 23
Hour 24
CHF210

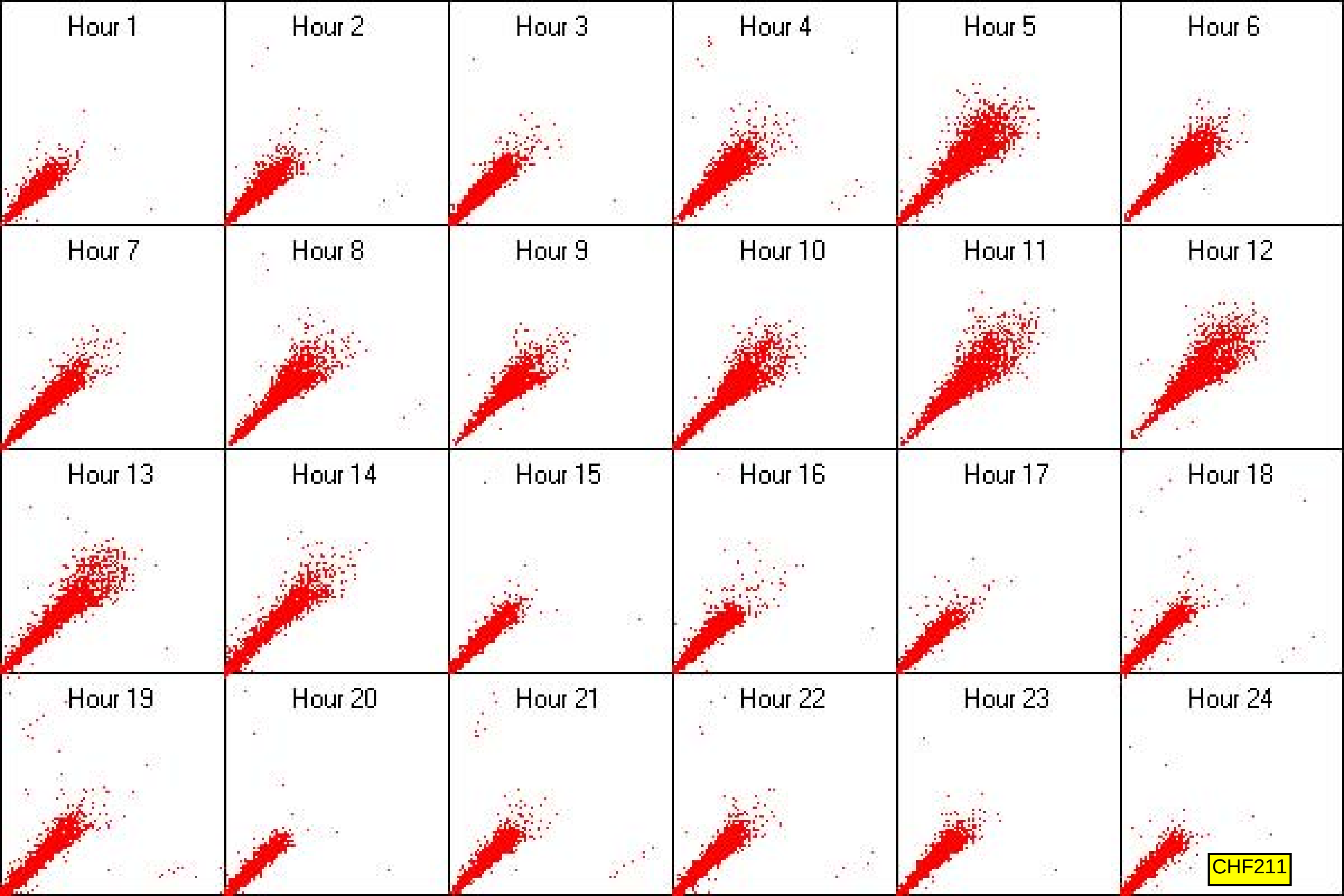
Hour 1
Hour 2
Hour 3
Hour 4
Hour 5
Hour 6
Hour 7
Hour 8
Hour 9
Hour 10
Hour 11
Hour 12
Hour 13
Hour 14
Hour 15
Hour 16
Hour 17
Hour 18
Hour 19
Hour 20
Hour 21
Hour 22
Hour 23
Hour 24
CHF211

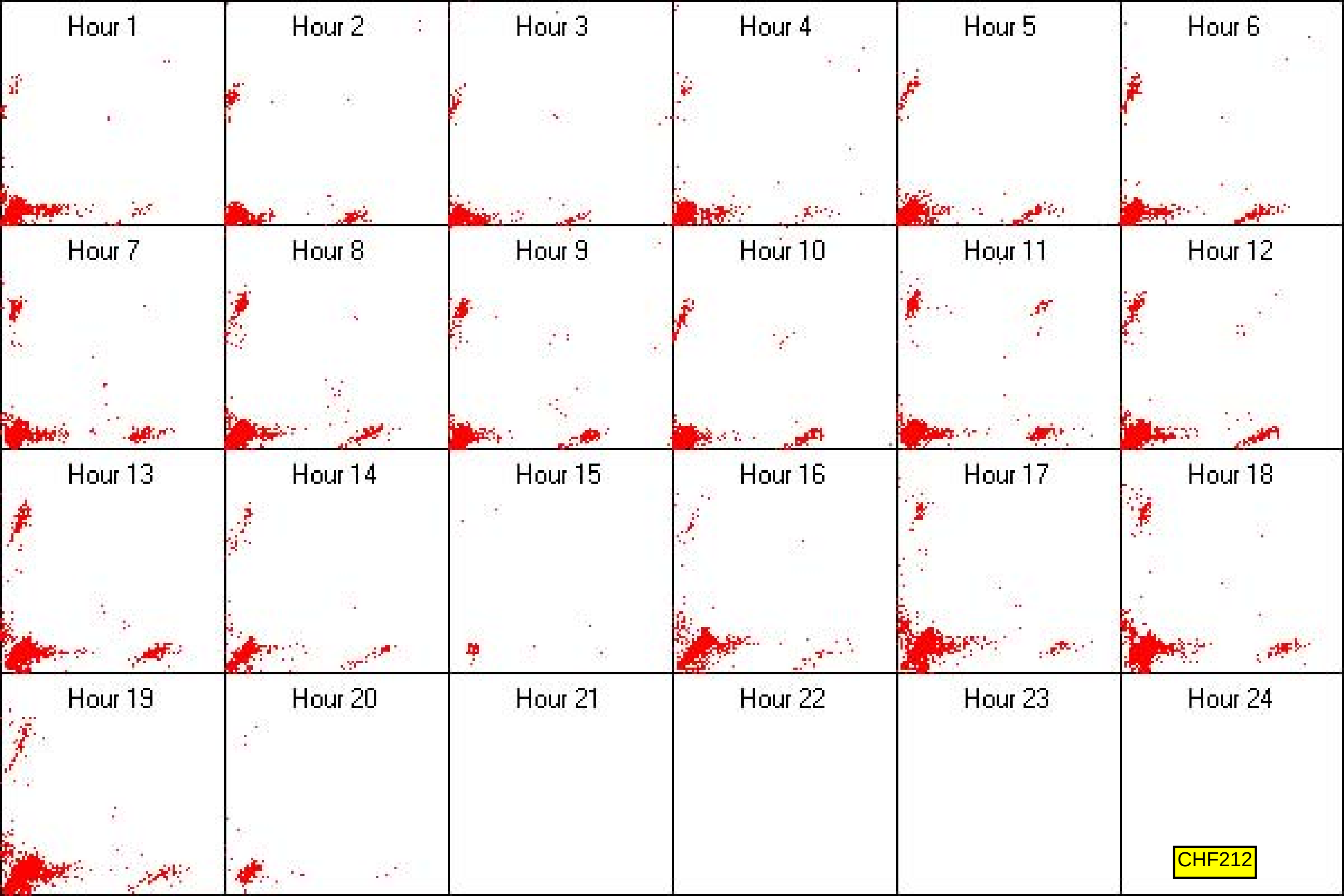
Hour 1
Hour 2
Hour 3
Hour 4
Hour 5
Hour 6
Hour 7
Hour 8
Hour 9
Hour 10
Hour 11
Hour 12
Hour 13
Hour 14
Hour 15
Hour 16
Hour 17
Hour 18
Hour 19
Hour 20
Hour 21
Hour 22
Hour 23
Hour 24
CHF212

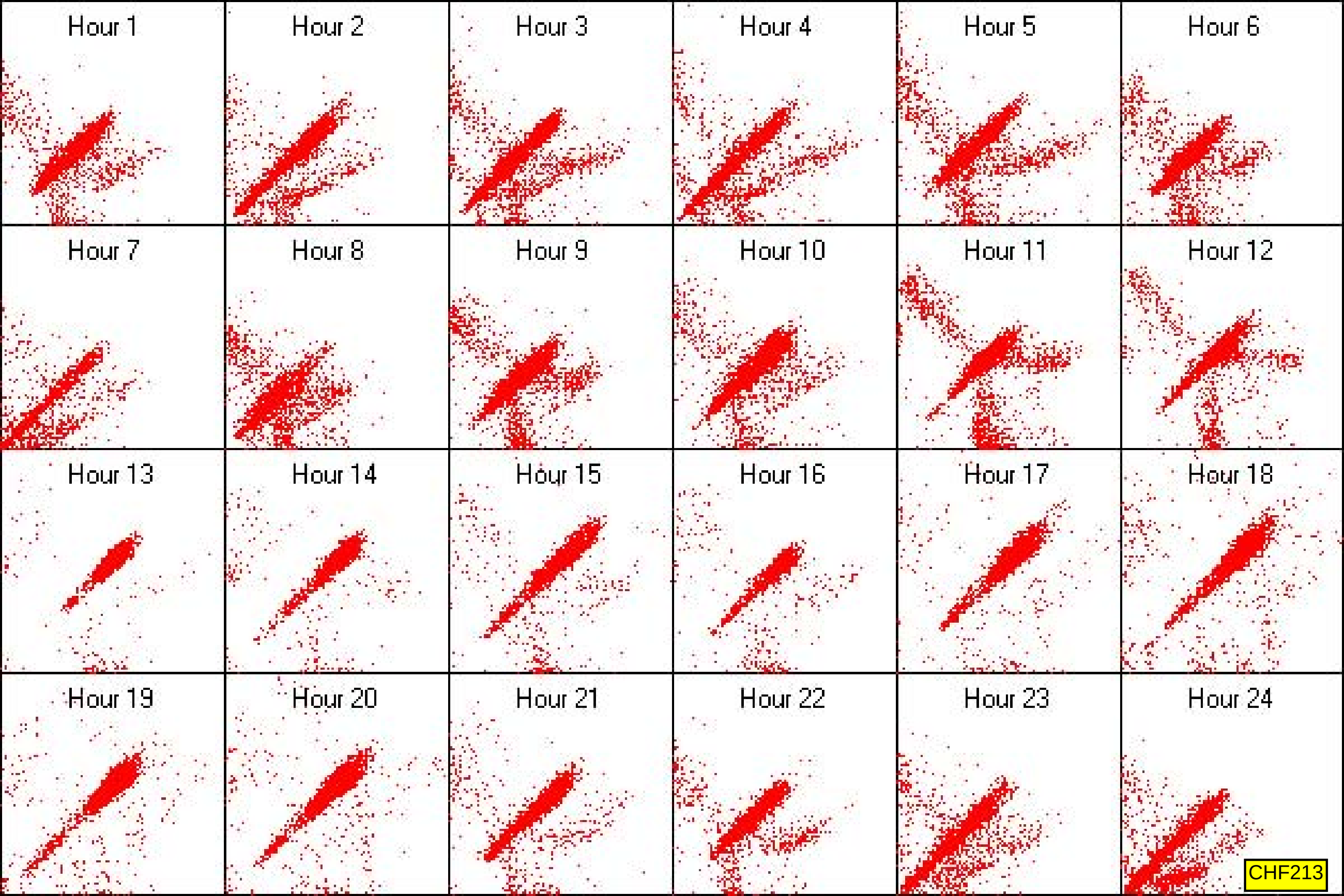
Hour 1
Hour 2
Hour 3
Hour 4
Hour 5
Hour 6
Hour 7
Hour 8
Hour 9
Hour 10
Hour 11
Hour 12
Hour 13
Hour 14
Hour 15
Hour 16
Hour 17
Hour 18
Hour 19
Hour 20
Hour 21
Hour 22
Hour 23
Hour 24
CHF213

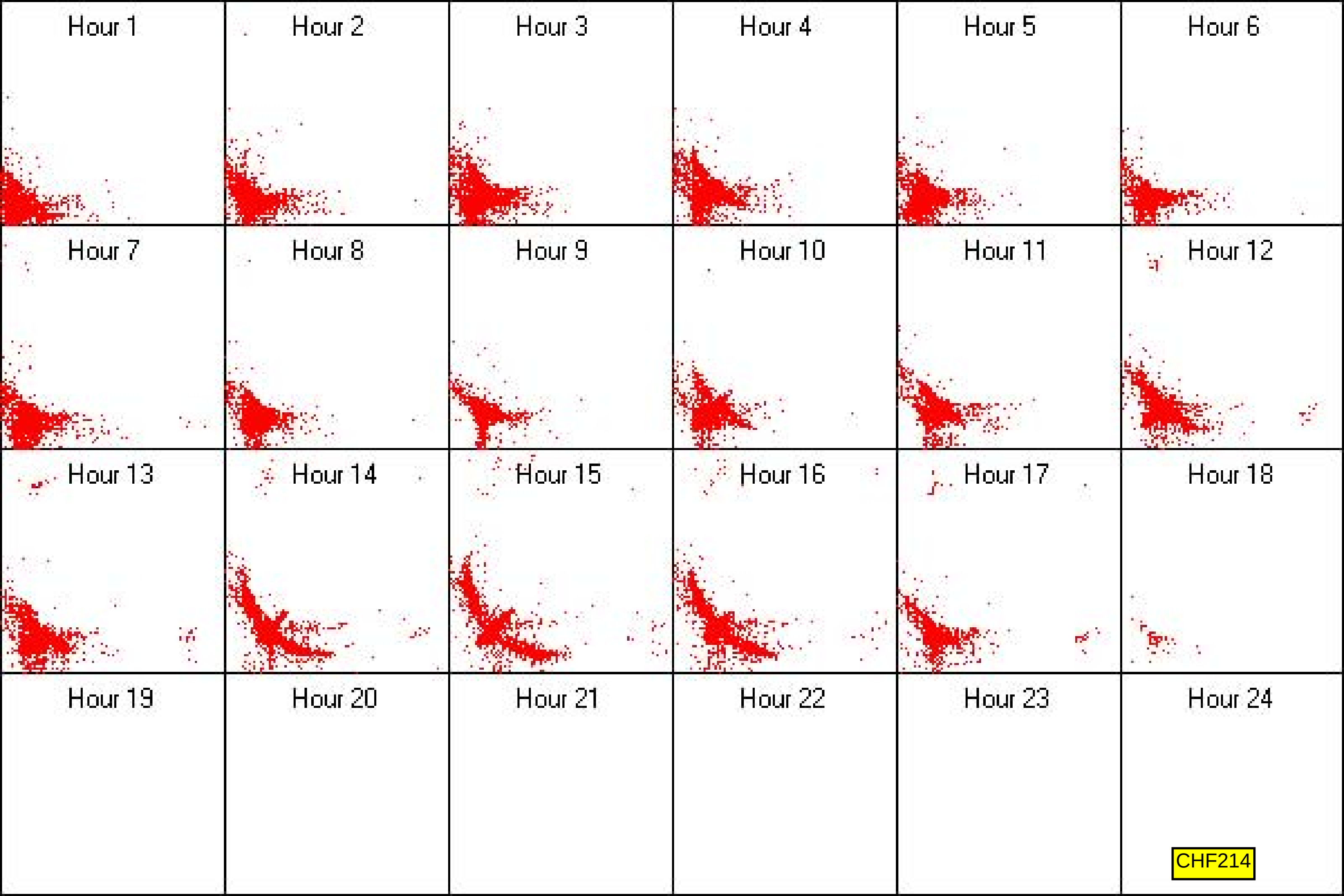

Hour 1
Hour 2
Hour 3
Hour 4
Hour 5
Hour 6
Hour 7
Hour 8
Hour 9
Hour 10
Hour 11
Hour 12
Hour 13
Hour 14
Hour 15
Hour 16
Hour 17
Hour 18
Hour 19
Hour 20
Hour 21
Hour 22
Hour 23
Hour 24
CHF214

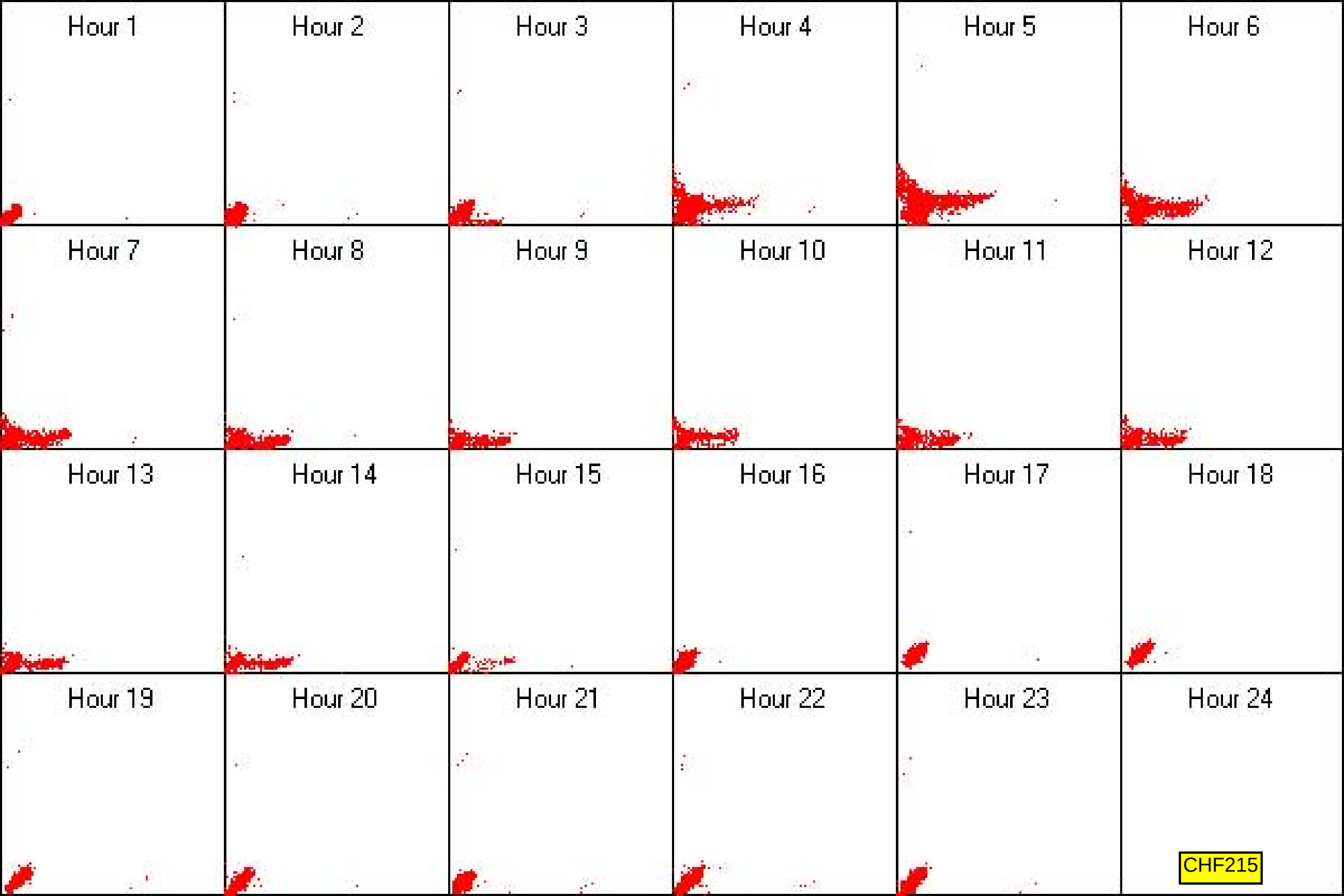
Hour 1
Hour 2
Hour 3
Hour 4
Hour 5
Hour 6
Hour 7
Hour 8
Hour 9
Hour 10
Hour 11
Hour 12
Hour 13
Hour 14
Hour 15
Hour 16
Hour 17
Hour 18
Hour 19
Hour 20
Hour 21
Hour 22
Hour 23
Hour 24
CHF215

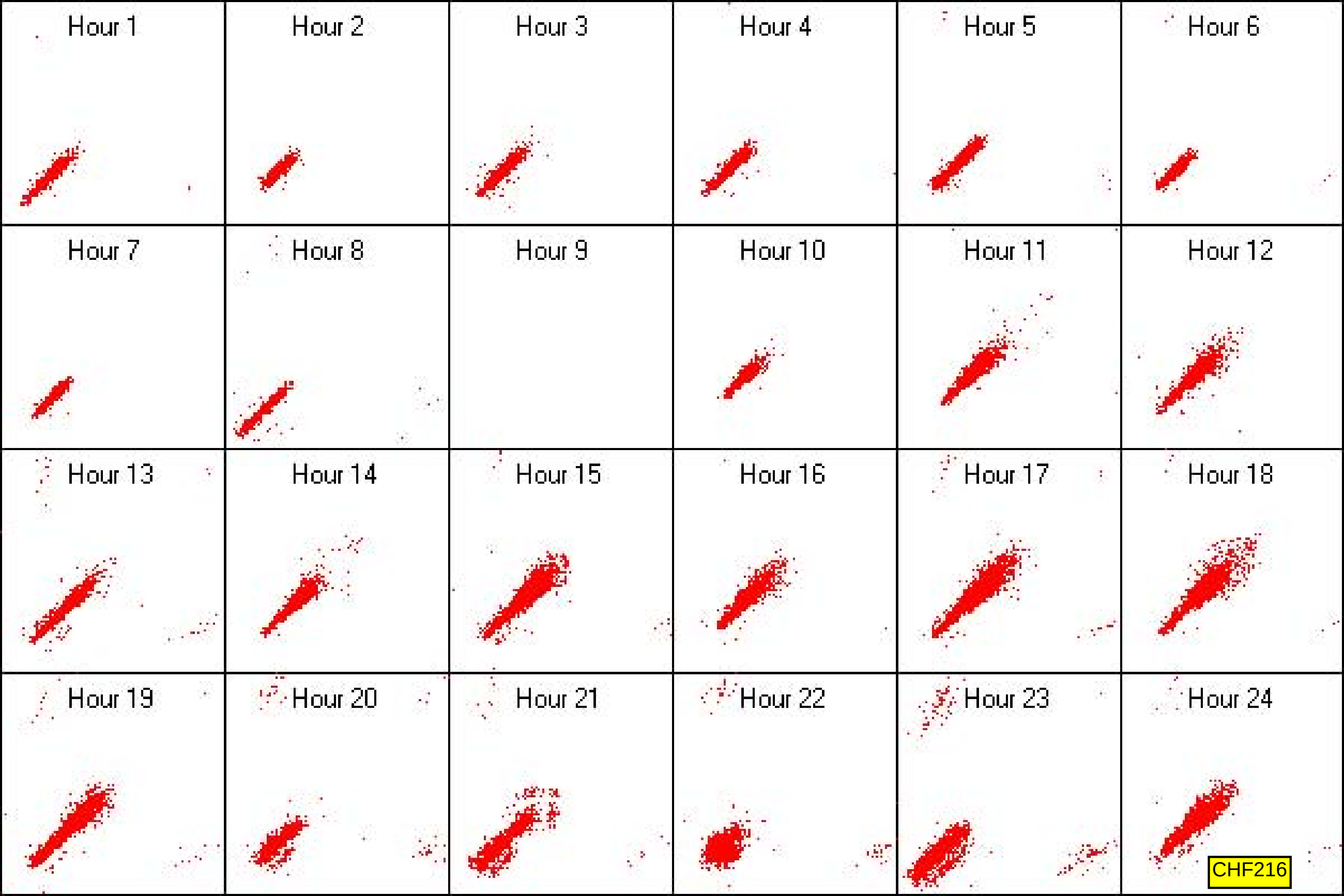
Hour 1
Hour 2
Hour 3
Hour 4
Hour 5
Hour 6
Hour 7
Hour 8
Hour 9
Hour 10
Hour 11
Hour 12
Hour 13
Hour 14
Hour 15
Hour 16
Hour 17
Hour 18
Hour 19
Hour 20
Hour 21
Hour 22
Hour 23
Hour 24
CHF216

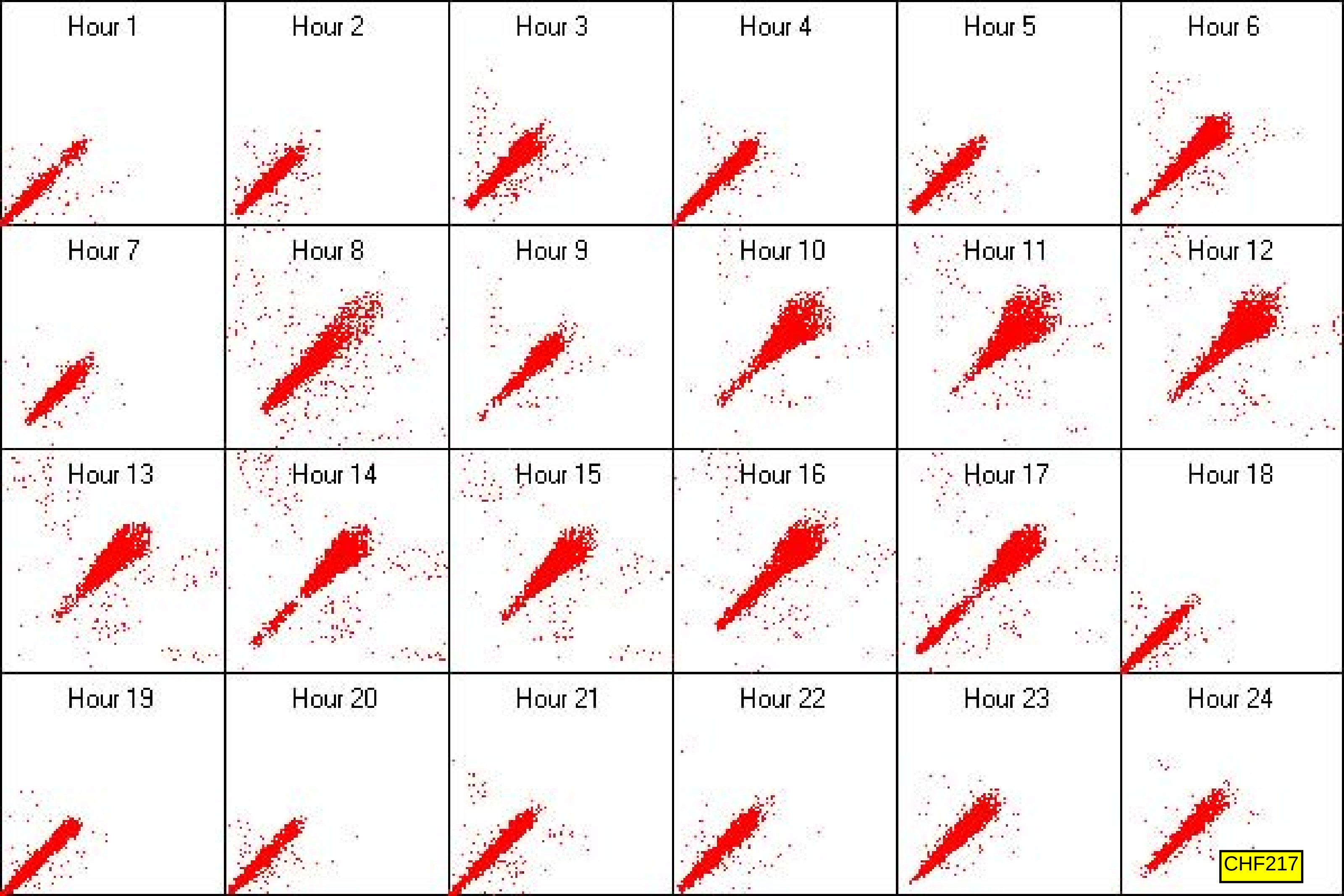
Hour 1
Hour 2
Hour 3
Hour 4
Hour 5
Hour 6
Hour 7
Hour 8
Hour 9
Hour 10
Hour 11
Hour 12
Hour 13
Hour 14
Hour 15
Hour 16
Hour 17
Hour 18
Hour 19
Hour 20
Hour 21
Hour 22
Hour 23
Hour 24
CHF217

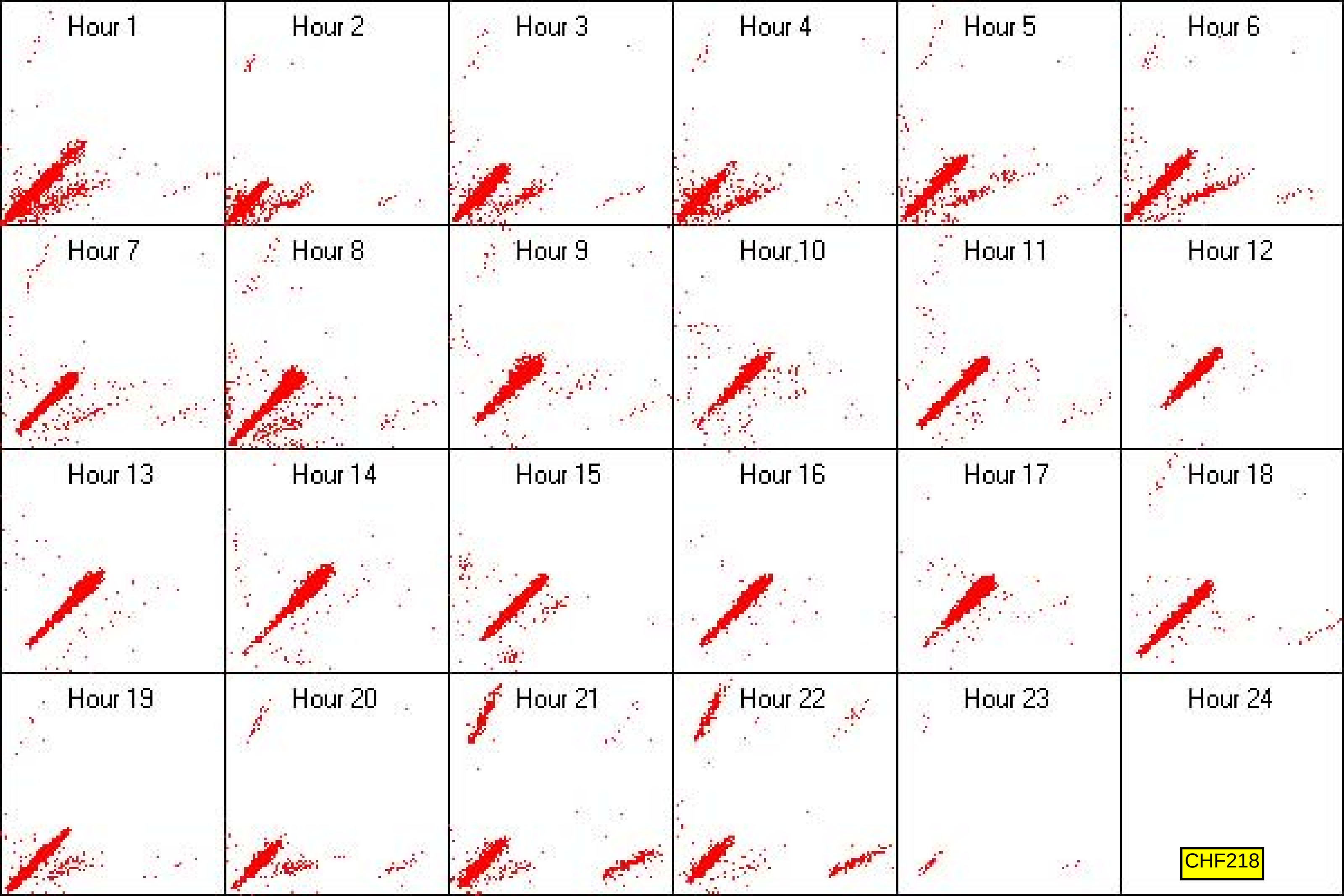
Hour 1
Hour 2
Hour 3
Hour 4
Hour 5
Hour 6
Hour 7
Hour 8
Hour 9
Hour 10
Hour 11
Hour 12
Hour 13
Hour 14
Hour 15
Hour 16
Hour 17
Hour 18
Hour 19
Hour 20
Hour 21
Hour 22
Hour 23
Hour 24
CHF218

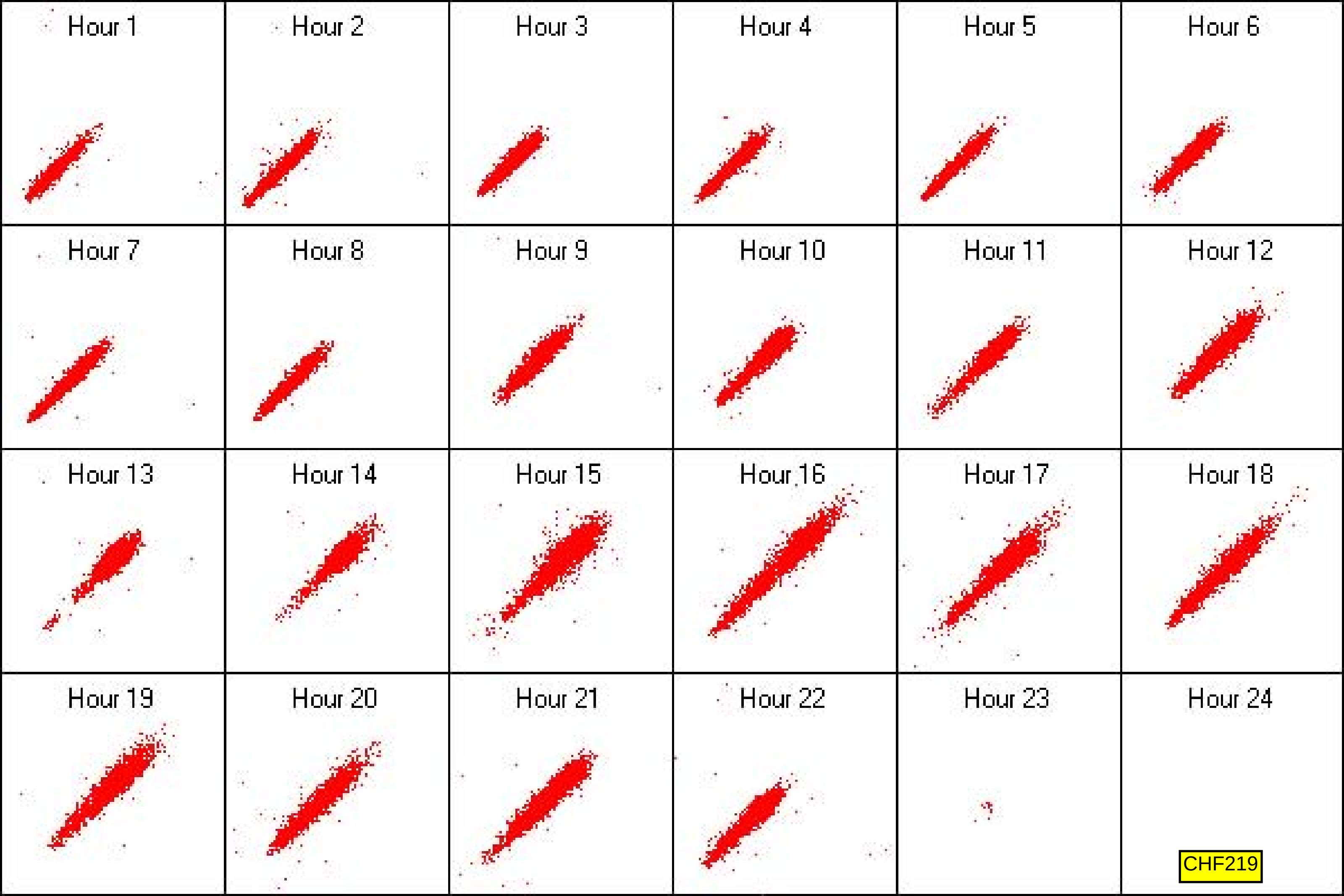

Hour 1
Hour 2
Hour 3
Hour 4
Hour 5
Hour 6
Hour 7
Hour 8
Hour 9
Hour 10
Hour 11
Hour 12
Hour 13
Hour 14
Hour 15
Hour 16
Hour 17
Hour 18
Hour 19
Hour 20
Hour 21
Hour 22
Hour 23
Hour 24
CHF219

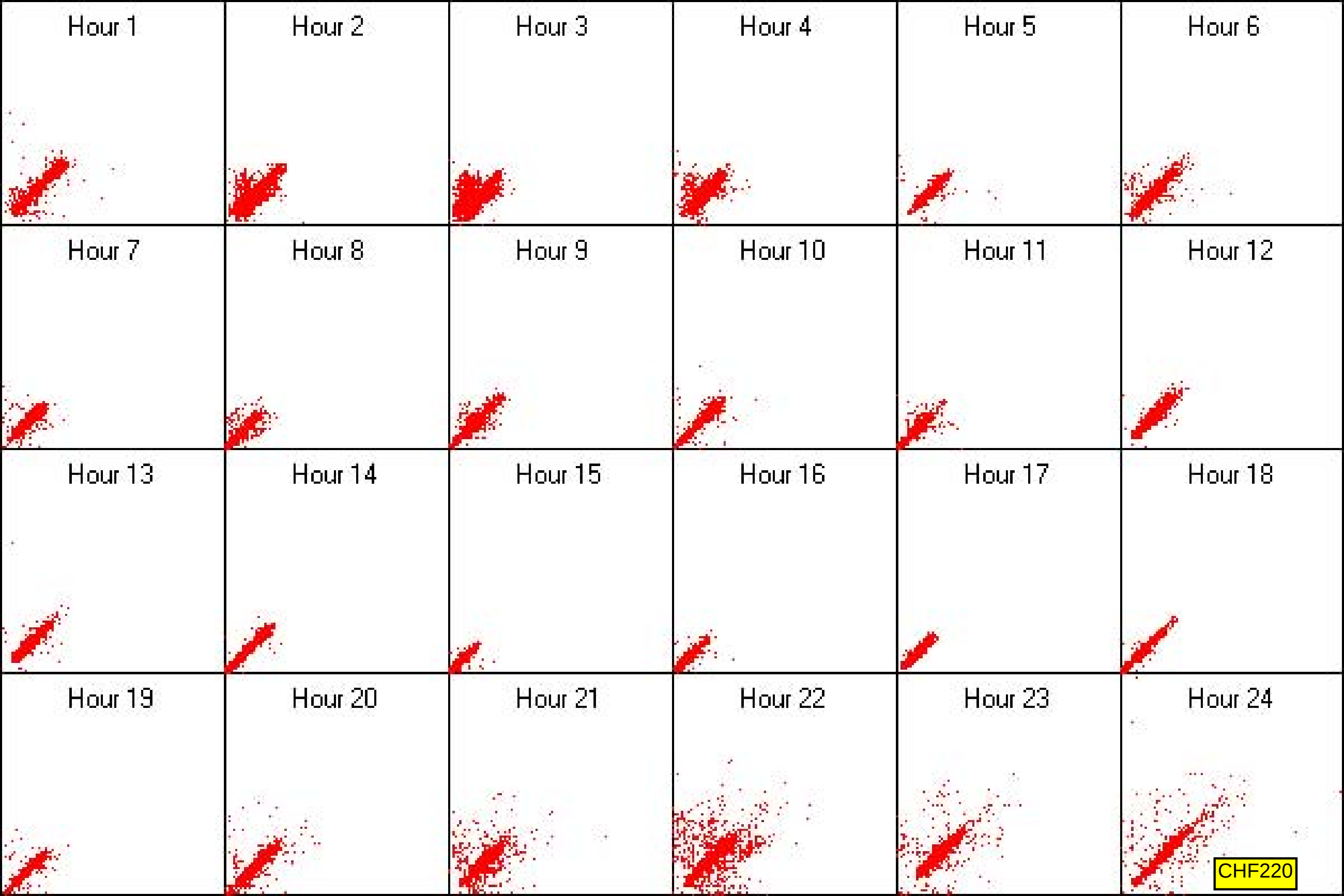
Hour 1
Hour 2
Hour 3
Hour 4
Hour 5
Hour 6
Hour 7
Hour 8
Hour 9
Hour 10
Hour 11
Hour 12
Hour 13
Hour 14
Hour 15
Hour 16
Hour 17
Hour 18
Hour 19
Hour 20
Hour 21
Hour 22
Hour 23
Hour 24
CHF220

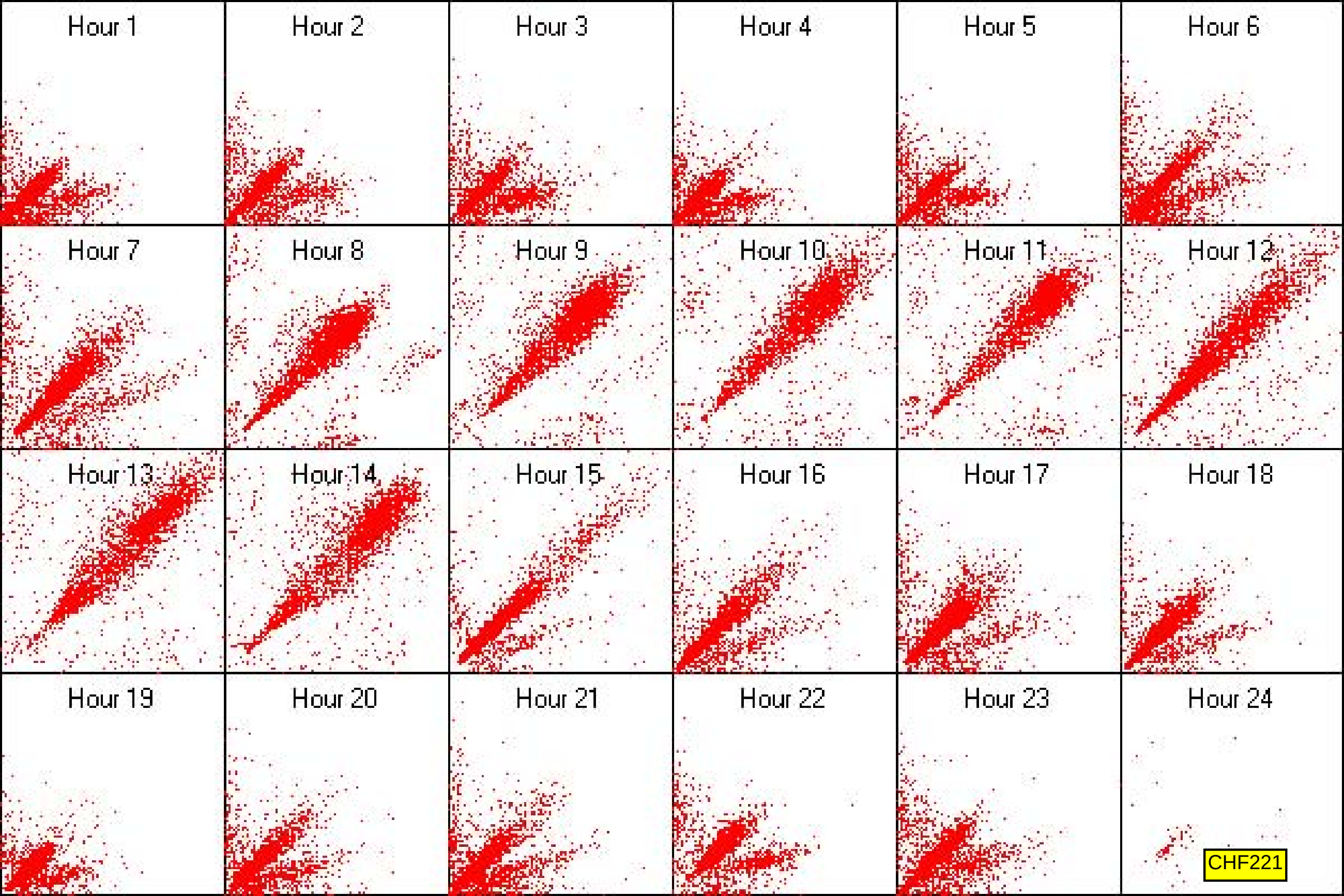
Hour 1
Hour 2
Hour 3
Hour 4
Hour 5
Hour 6
Hour 7
Hour 8
Hour 9
Hour 10
Hour 11
Hour 12
Hour 13
Hour 14
Hour 15
Hour 16
Hour 17
Hour 18
Hour 19
Hour 20
Hour 21
Hour 22
Hour 23
Hour 24
CHF221

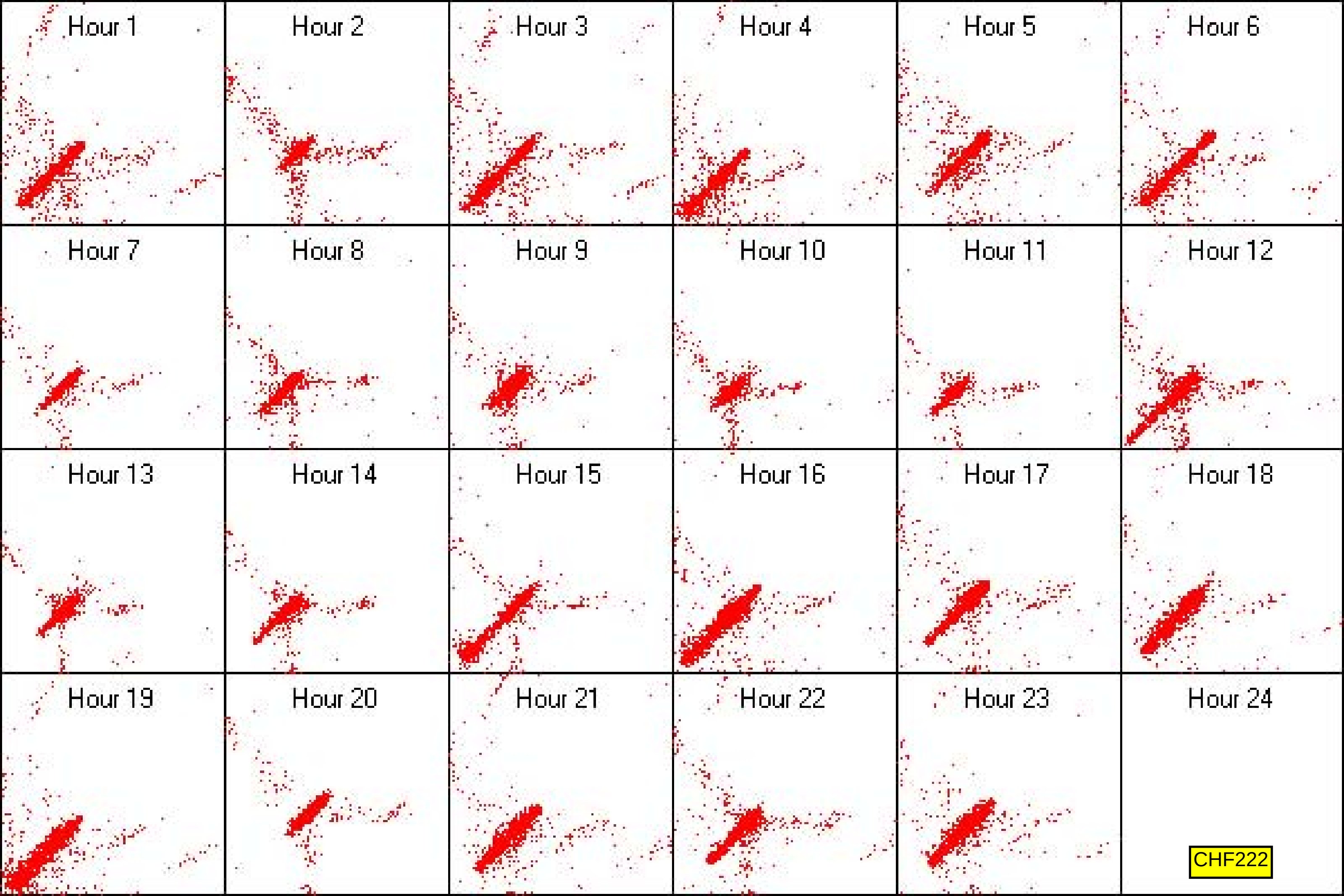

Hour 1
Hour 2
Hour 3
Hour 4
Hour 5
Hour 6
Hour 7
Hour 8
Hour 9
Hour 10
Hour 11
Hour 12
Hour 13
Hour 14
Hour 15
Hour 16
Hour 17
Hour 18
Hour 19
Hour 20
Hour 21
Hour 22
Hour 23
Hour 24
CHF222

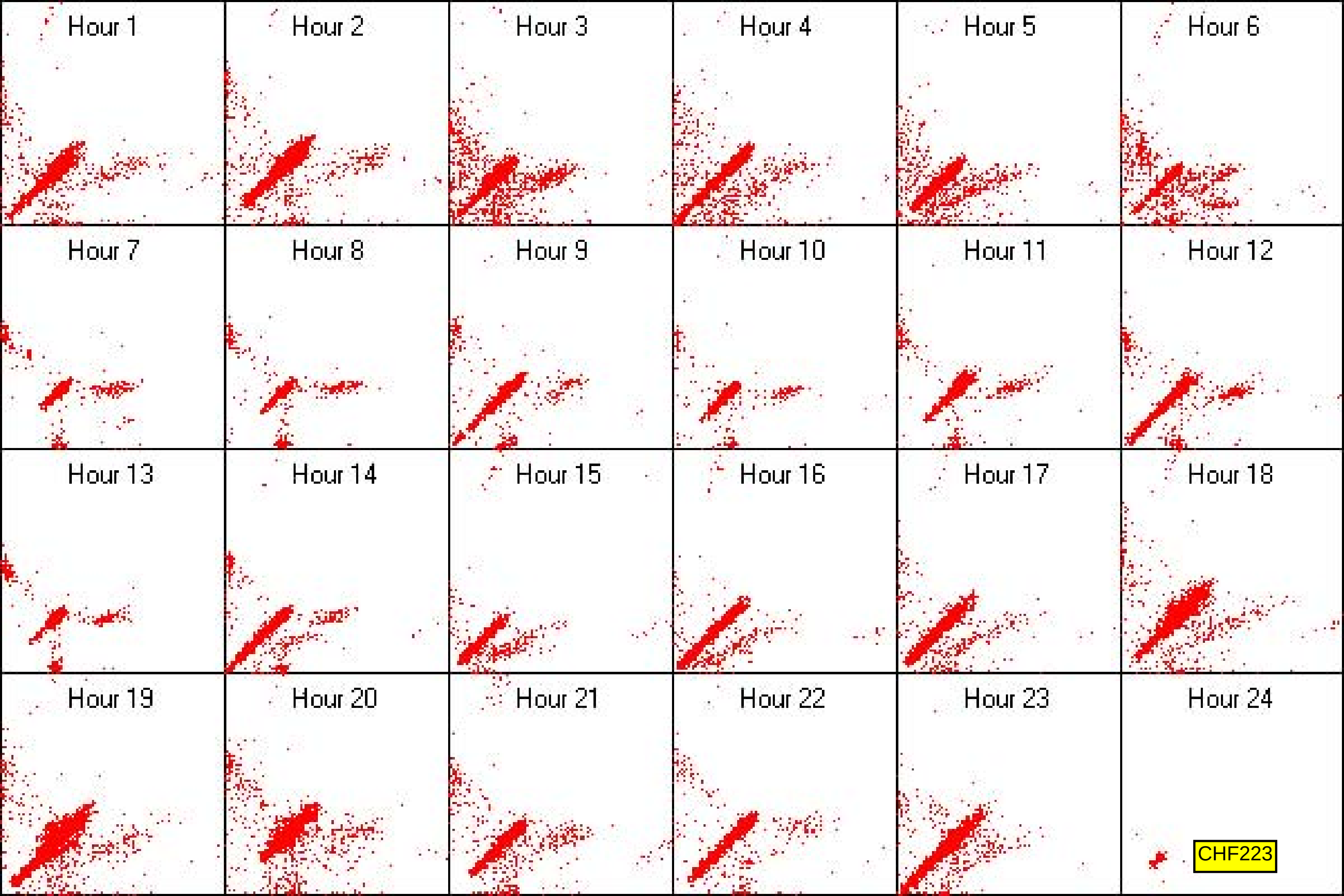

Hour 1
Hour 2
Hour 3
Hour 4
Hour 5
Hour 6
Hour 7
Hour 8
Hour 9
Hour 10
Hour 11
Hour 12
Hour 13
Hour 14
Hour 15
Hour 16
Hour 17
Hour 18
Hour 19
Hour 20
Hour 21
Hour 22
Hour 23
Hour 24
CHF223

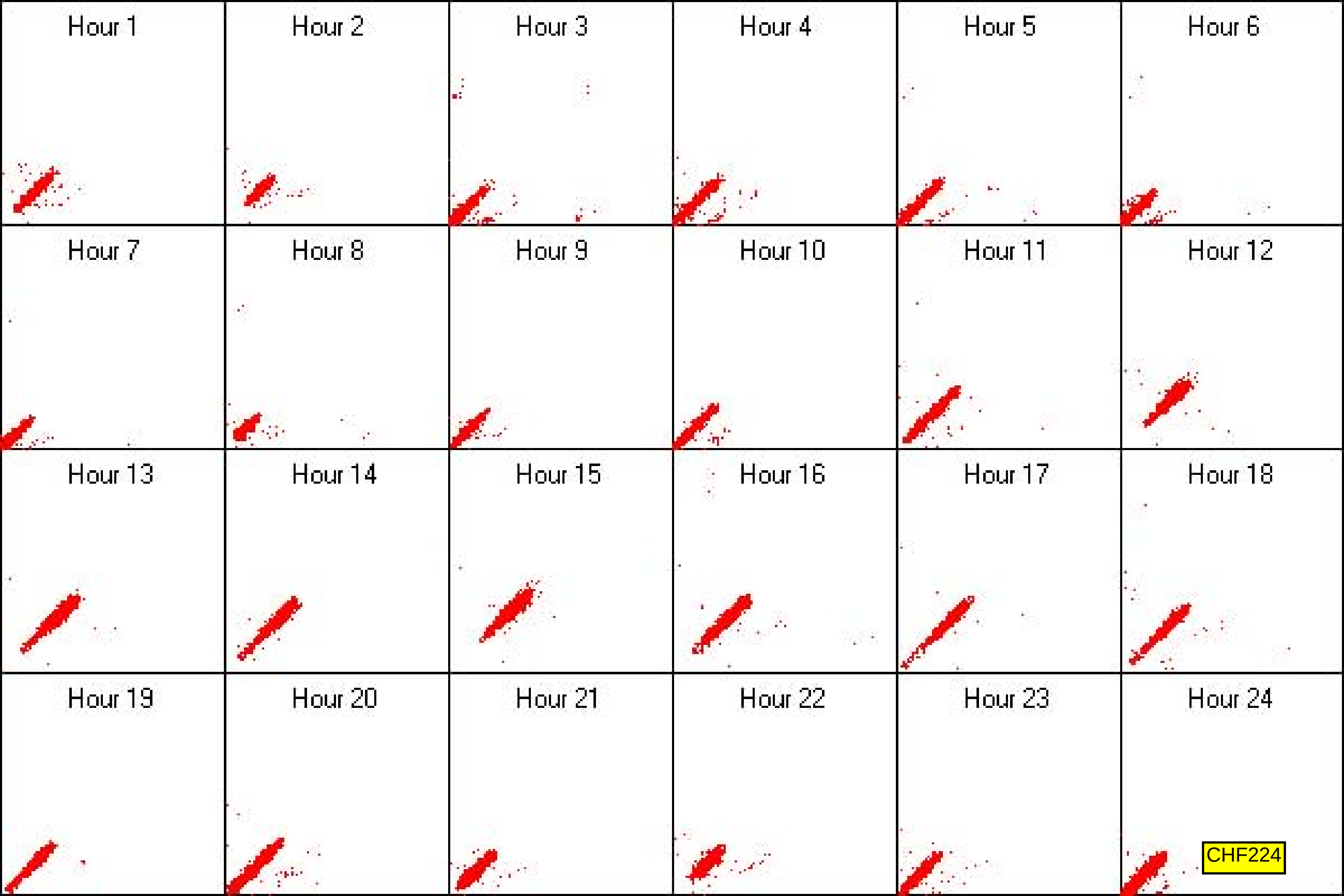
Hour 1
Hour 2
Hour 3
Hour 4
Hour 5
Hour 6
Hour 7
Hour 8
Hour 9
Hour 10
Hour 11
Hour 12
Hour 13
Hour 14
Hour 15
Hour 16
Hour 17
Hour 18
Hour 19
Hour 20
Hour 21
Hour 22
Hour 23
Hour 24
CHF224

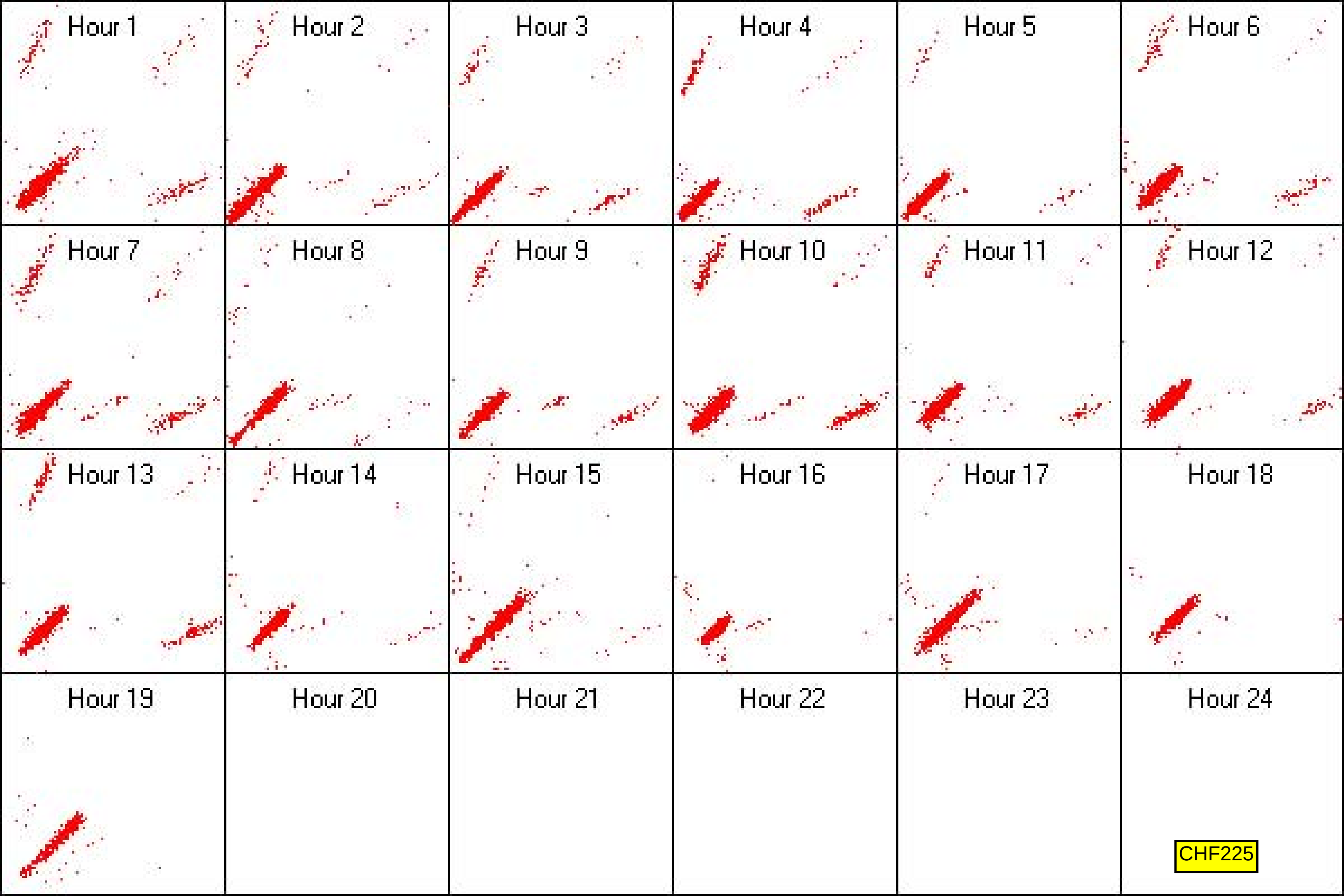
Hour 1
Hour 2
Hour 3
Hour 4
Hour 5
Hour 6
Hour 7
Hour 8
Hour 9
Hour 10
Hour 11
Hour 12
Hour 13
Hour 14
Hour 15
Hour 16
Hour 17
Hour 18
Hour 19
Hour 20
Hour 21
Hour 22
Hour 23
Hour 24
CHF225

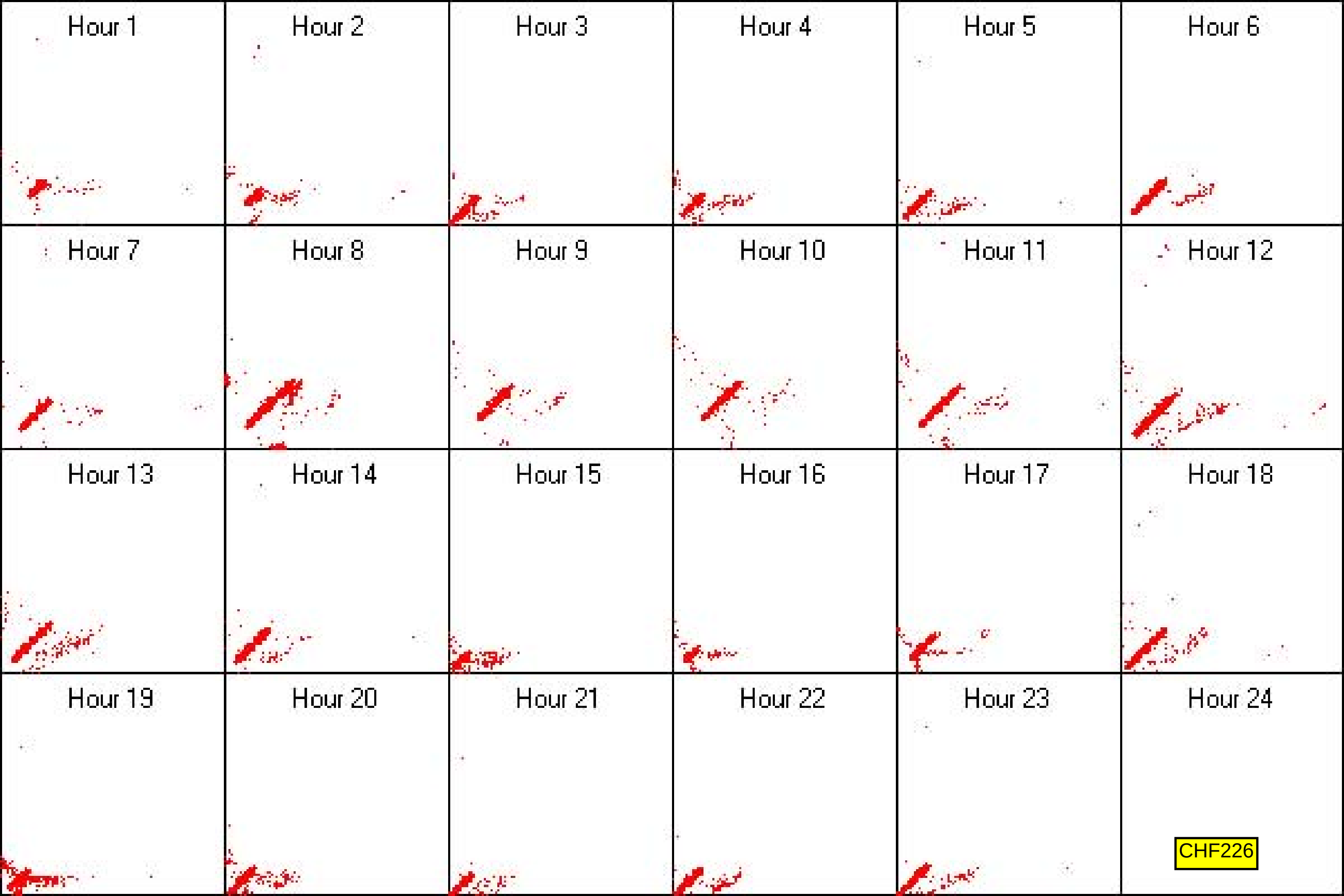
Hour 1
Hour 2
Hour 3
Hour 4
Hour 5
Hour 6
Hour 7
Hour 8
Hour 9
Hour 10
Hour 11
Hour 12
Hour 13
Hour 14
Hour 15
Hour 16
Hour 17
Hour 18
Hour 19
Hour 20
Hour 21
Hour 22
Hour 23
Hour 24
CHF226

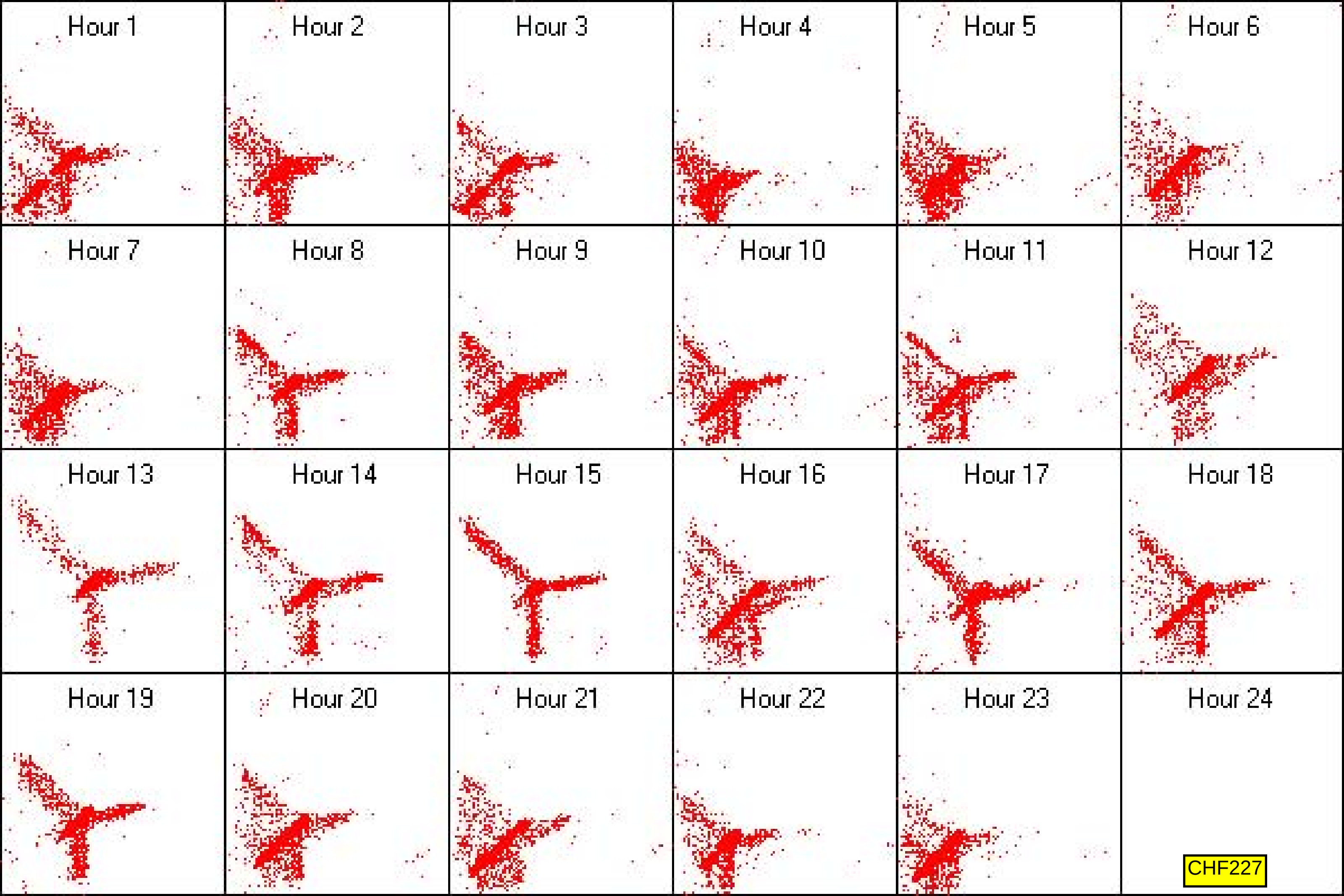
Hour 1
Hour 2
Hour 3
Hour 4
Hour 5
Hour 6
Hour 7
Hour 8
Hour 9
Hour 10
Hour 11
Hour 12
Hour 13
Hour 14
Hour 15
Hour 16
Hour 17
Hour 18
Hour 19
Hour 20
Hour 21
Hour 22
Hour 23
Hour 24
CHF227

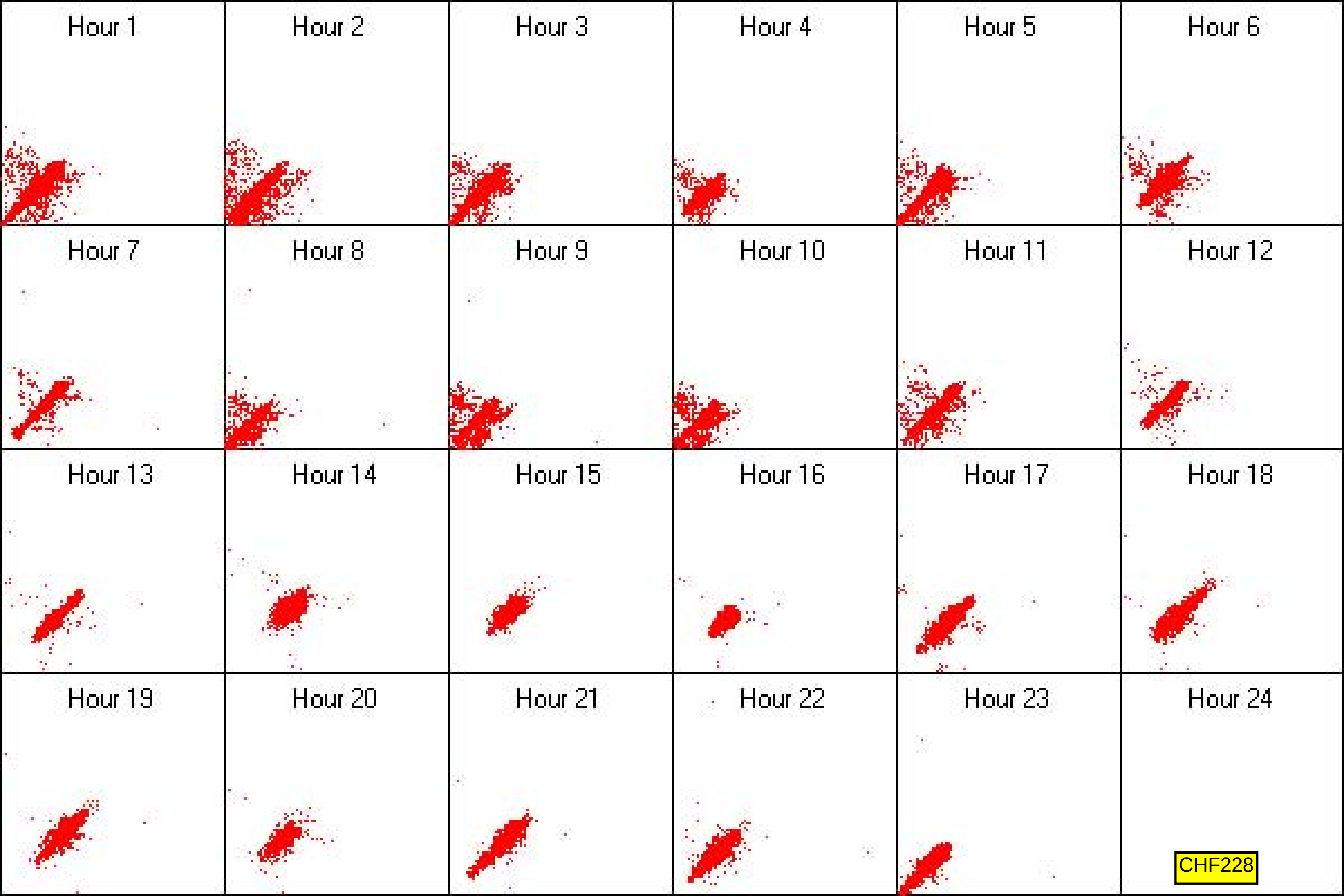

Hour 1
Hour 2
Hour 3
Hour 4
Hour 5
Hour 6
Hour 7
Hour 8
Hour 9
Hour 10
Hour 11
Hour 12
Hour 13
Hour 14
Hour 15
Hour 16
Hour 17
Hour 18
Hour 19
Hour 20
Hour 21
Hour 22
Hour 23
Hour 24
CHF228

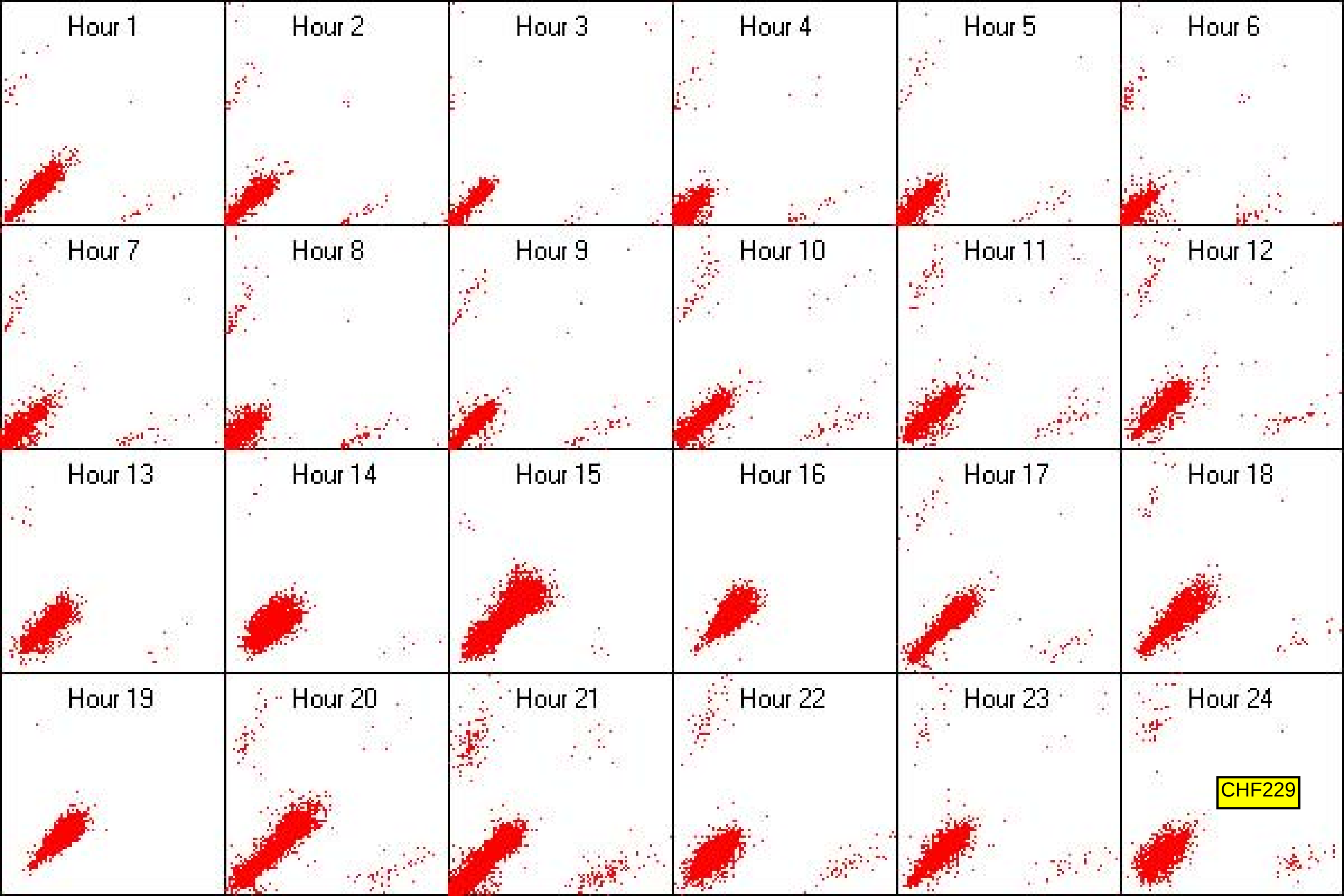
Hour 1
Hour 2
Hour 3
Hour 4
Hour 5
Hour 6
Hour 7
Hour 8
Hour 9
Hour 10
Hour 11
Hour 12
Hour 13
Hour 14
Hour 15
Hour 16
Hour 17
Hour 18
Hour 19
Hour 20
Hour 21
Hour 22
Hour 23
Hour 24
CHF229
