# Supplementary material for: Loss of lag-response curvilinearity of indices of heart rate variability in congestive heart failure
Source: BMC Cardiovasc Disord. 2006 Jun 12;6:27. doi: 10.1186/1471-2261-6-27 (PMC1523370; doi:10.1186/1471-2261-6-27)
Supplement: Additional file 3 — This file has hourly Poincaré plots for the 54 control subjects. [file 1471-2261-6-27-S3.pdf]

Hourly Poincaré Plots for NSR subjects

This file contains the hourly Poincaré plots of all the NSR patients the data on whom was used in the study by Thakre and Smith. All the plots are plotted on a square representing 0.5 to 1.5 seconds on both axes. The x-axis represents the current beat while the y-axis represents the next beat. The identifier for the study subject is shown in the box representing the hour 24 at the bottom of each page.

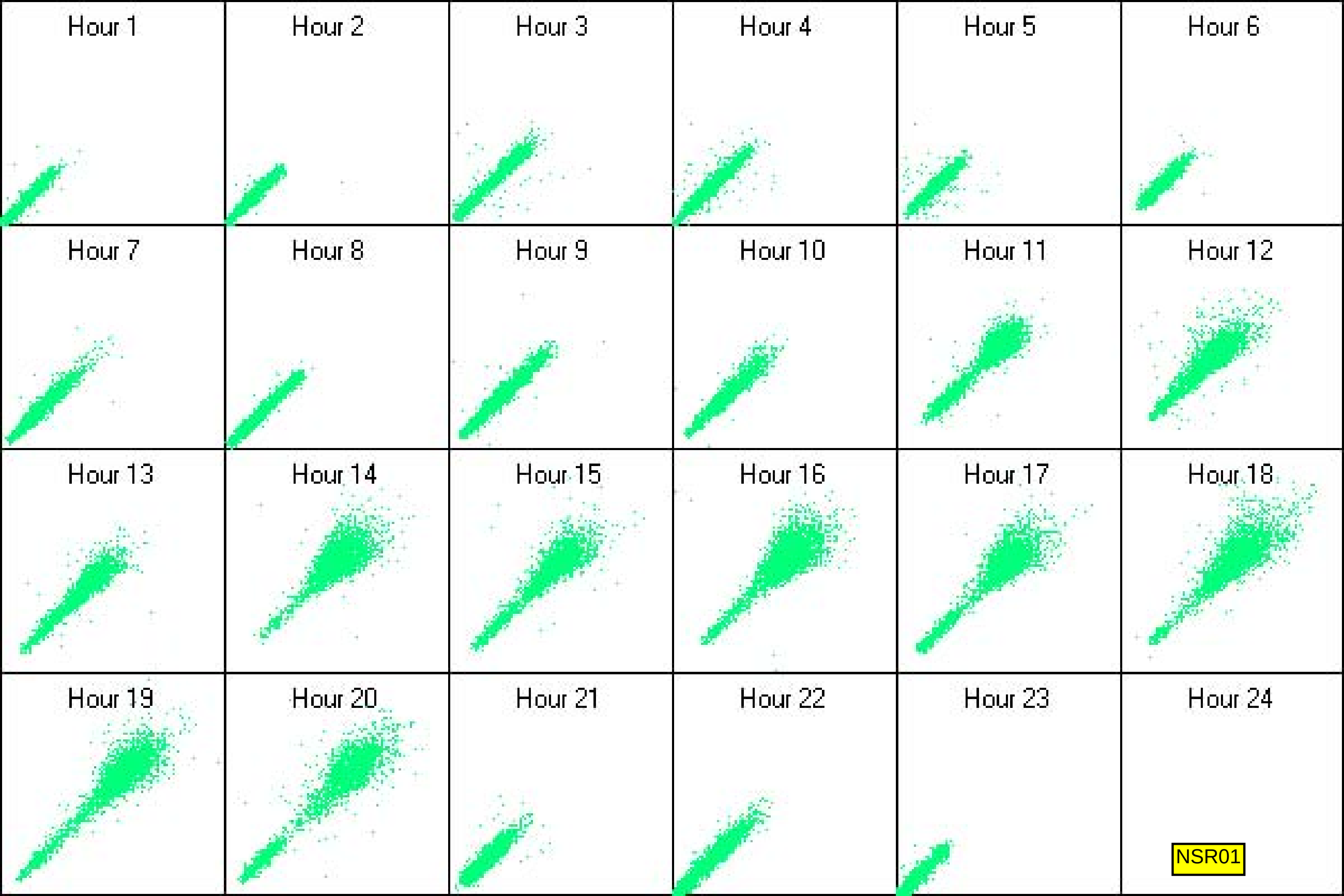
Hour 1
Hour 2
Hour 3
Hour 4
Hour 5
Hour 6
Hour 7
Hour 8
Hour 9
Hour 10
Hour 11
Hour 12
Hour 13
Hour 14
Hour 15
Hour 16
Hour 17
Hour 18
Hour 19
Hour 20
Hour 21
Hour 22
Hour 23
Hour 24
NSR01

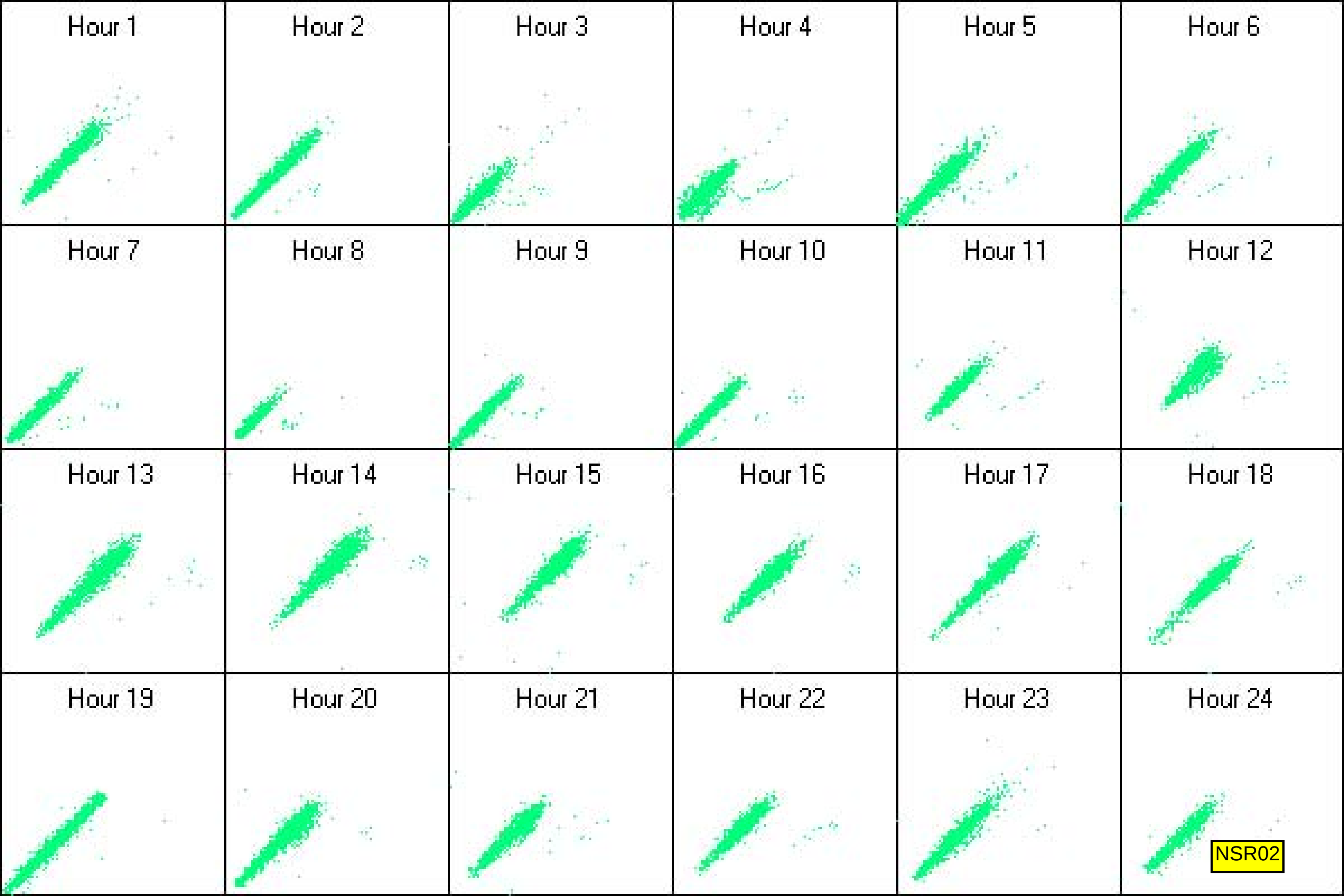
Hour 1
Hour 2
Hour 3
Hour 4
Hour 5
Hour 6
Hour 7
Hour 8
Hour 9
Hour 10
Hour 11
Hour 12
Hour 13
Hour 14
Hour 15
Hour 16
Hour 17
Hour 18
Hour 19
Hour 20
Hour 21
Hour 22
Hour 23
Hour 24
NSR02

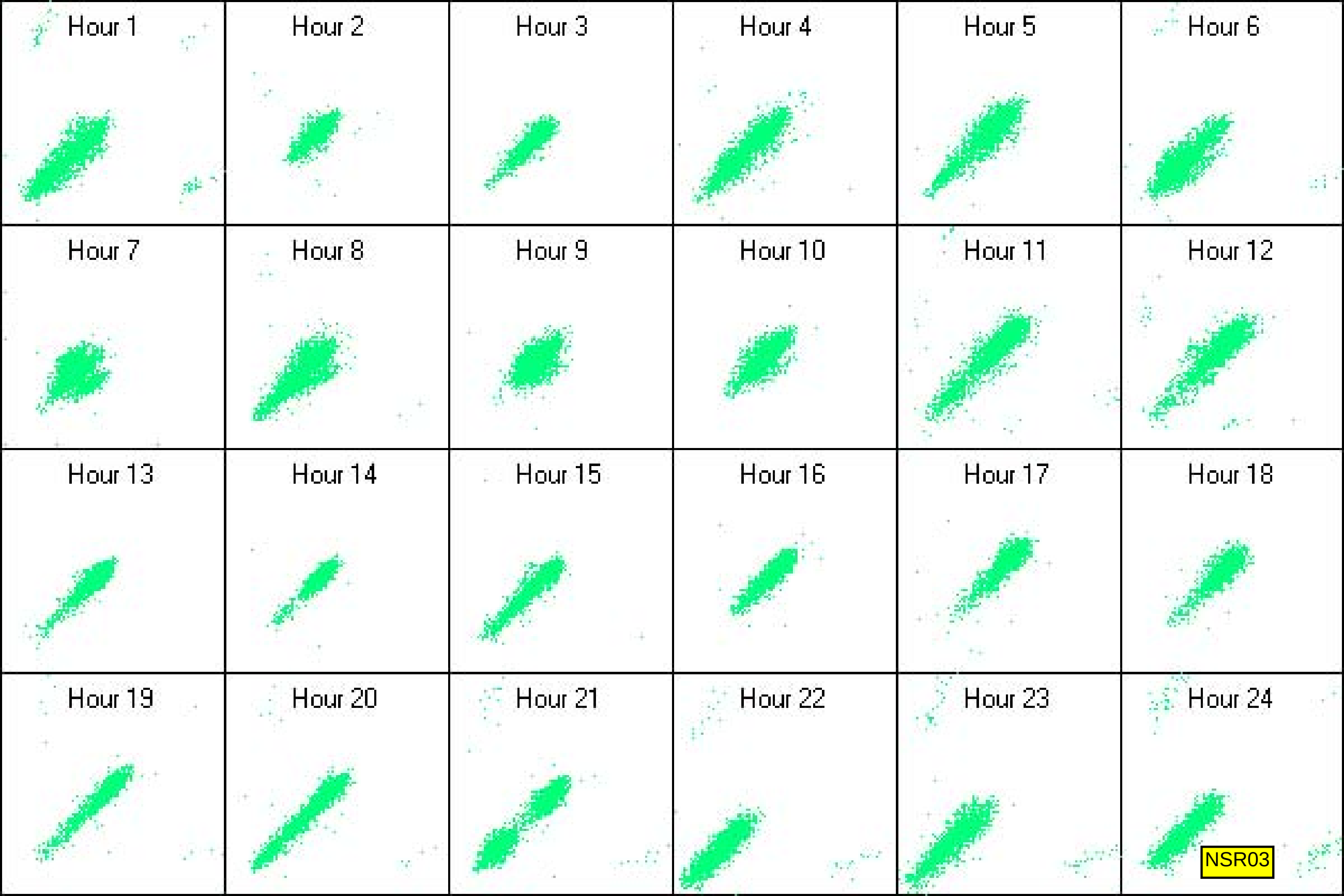

Hour 1
Hour 2
Hour 3
Hour 4
Hour 5
Hour 6
Hour 7
Hour 8
Hour 9
Hour 10
Hour 11
Hour 12
Hour 13
Hour 14
Hour 15
Hour 16
Hour 17
Hour 18
Hour 19
Hour 20
Hour 21
Hour 22
Hour 23
Hour 24
NSR03

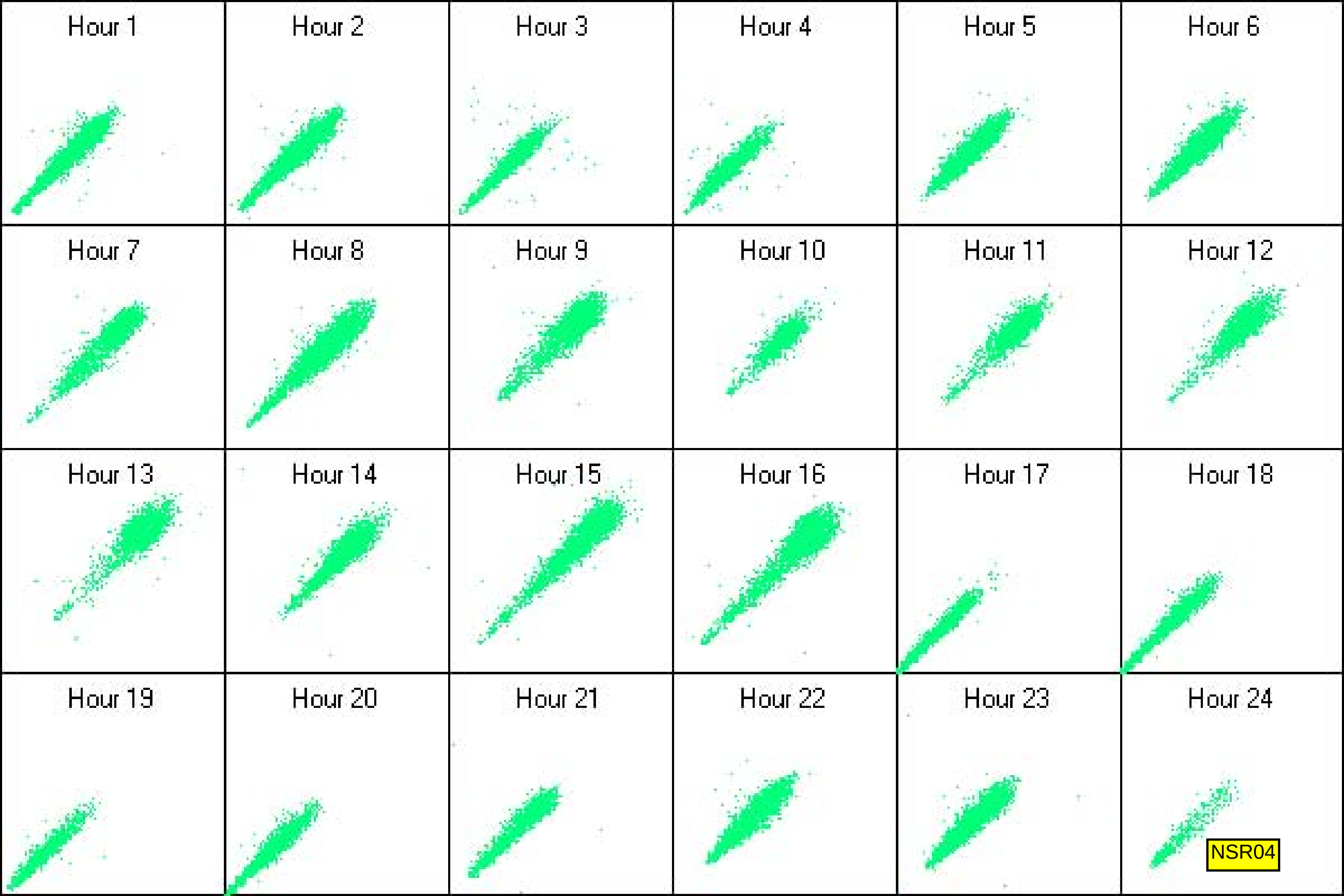
Hour 1
Hour 2
Hour 3
Hour 4
Hour 5
Hour 6
Hour 7
Hour 8
Hour 9
Hour 10
Hour 11
Hour 12
Hour 13
Hour 14
Hour 15
Hour 16
Hour 17
Hour 18
Hour 19
Hour 20
Hour 21
Hour 22
Hour 23
Hour 24
NSR04

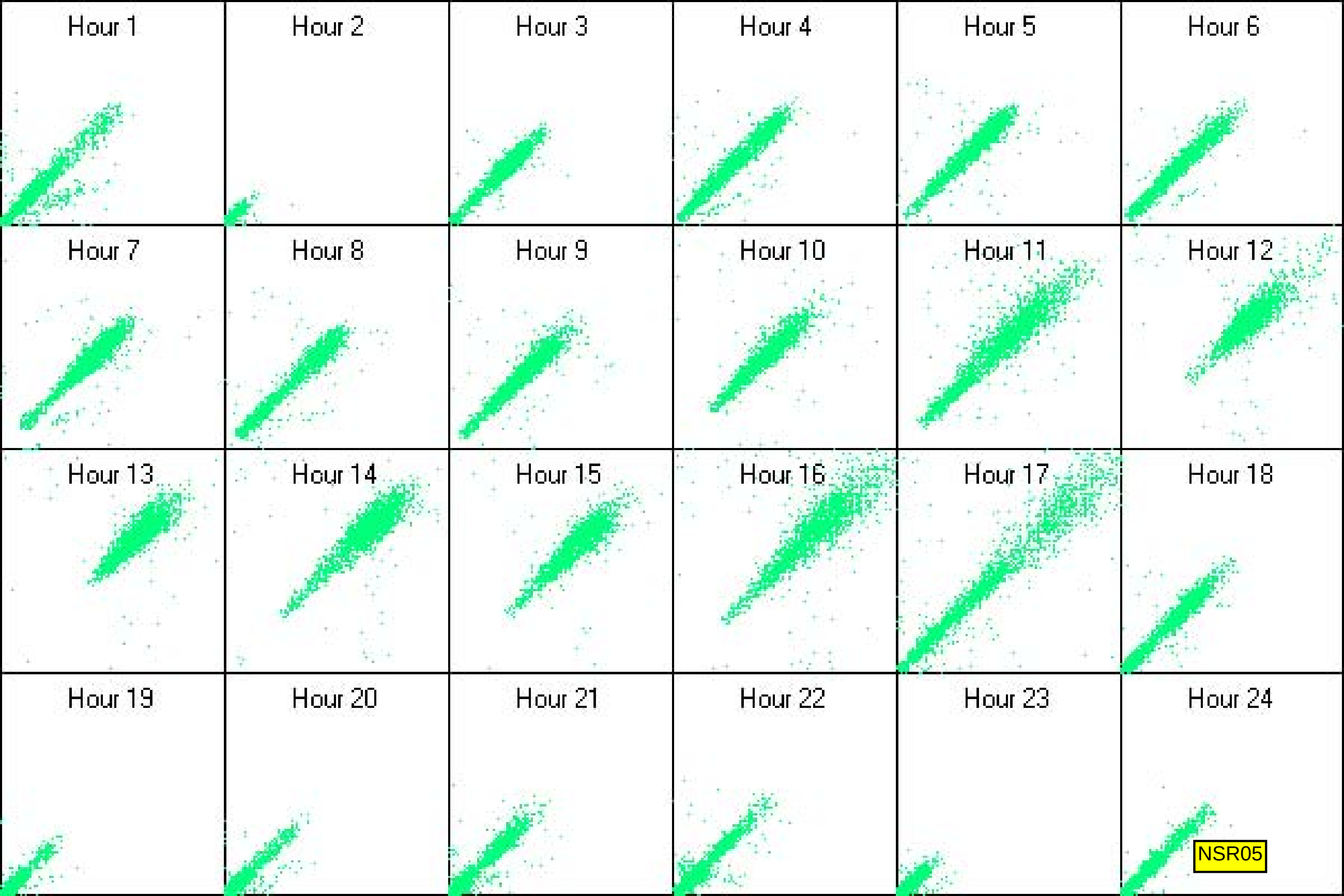
Hour 1
Hour 2
Hour 3
Hour 4
Hour 5
Hour 6
Hour 7
Hour 8
Hour 9
Hour 10
Hour 11
Hour 12
Hour 13
Hour 14
Hour 15
Hour 16
Hour 17
Hour 18
Hour 19
Hour 20
Hour 21
Hour 22
Hour 23
Hour 24
NSR05

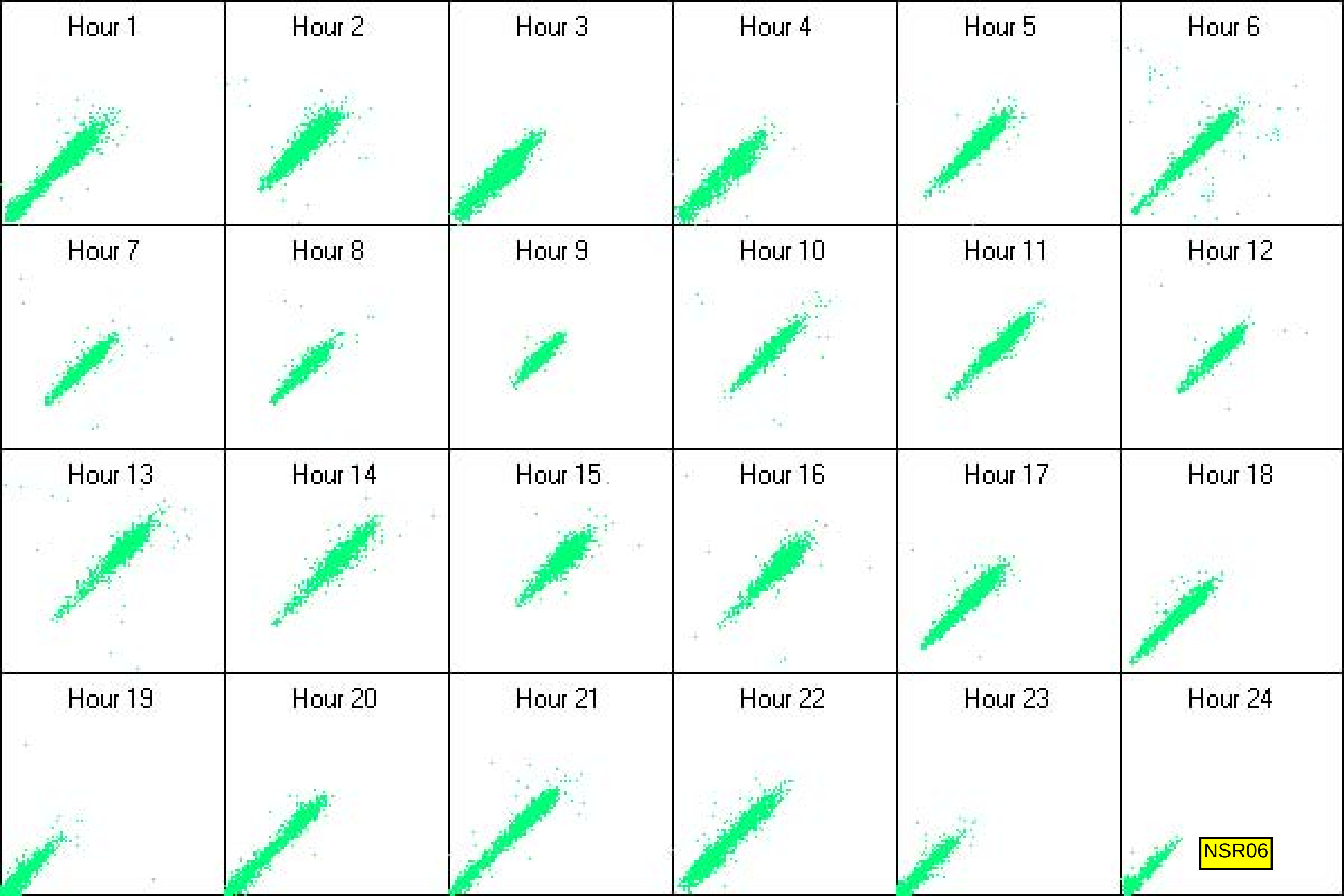
Hour 1
Hour 2
Hour 3
Hour 4
Hour 5
Hour 6
Hour 7
Hour 8
Hour 9
Hour 10
Hour 11
Hour 12
Hour 13
Hour 14
Hour 15
Hour 16
Hour 17
Hour 18
Hour 19
Hour 20
Hour 21
Hour 22
Hour 23
Hour 24
NSR06

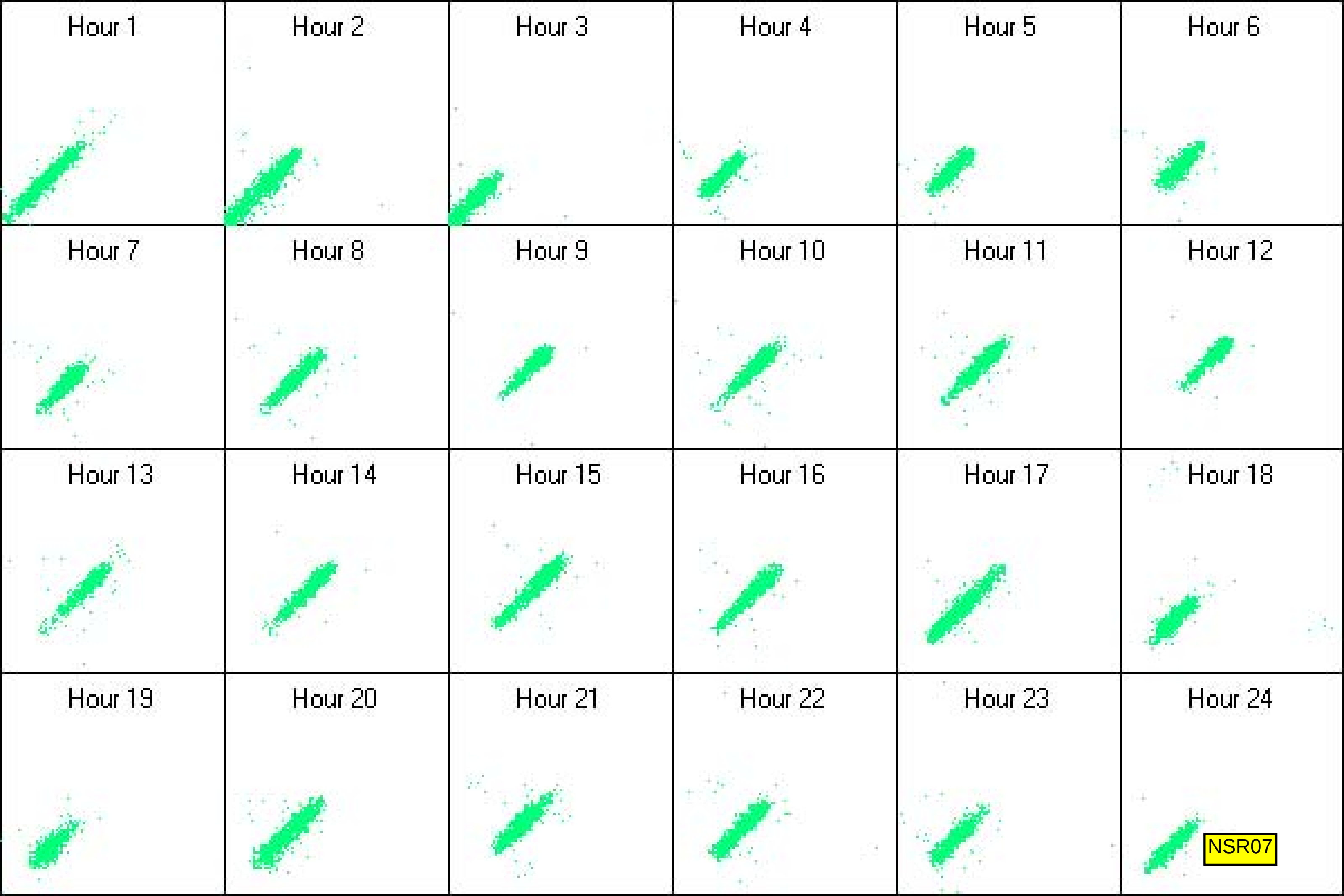
Hour 1
Hour 2
Hour 3
Hour 4
Hour 5
Hour 6
Hour 7
Hour 8
Hour 9
Hour 10
Hour 11
Hour 12
Hour 13
Hour 14
Hour 15
Hour 16
Hour 17
Hour 18
Hour 19
Hour 20
Hour 21
Hour 22
Hour 23
Hour 24
NSR07

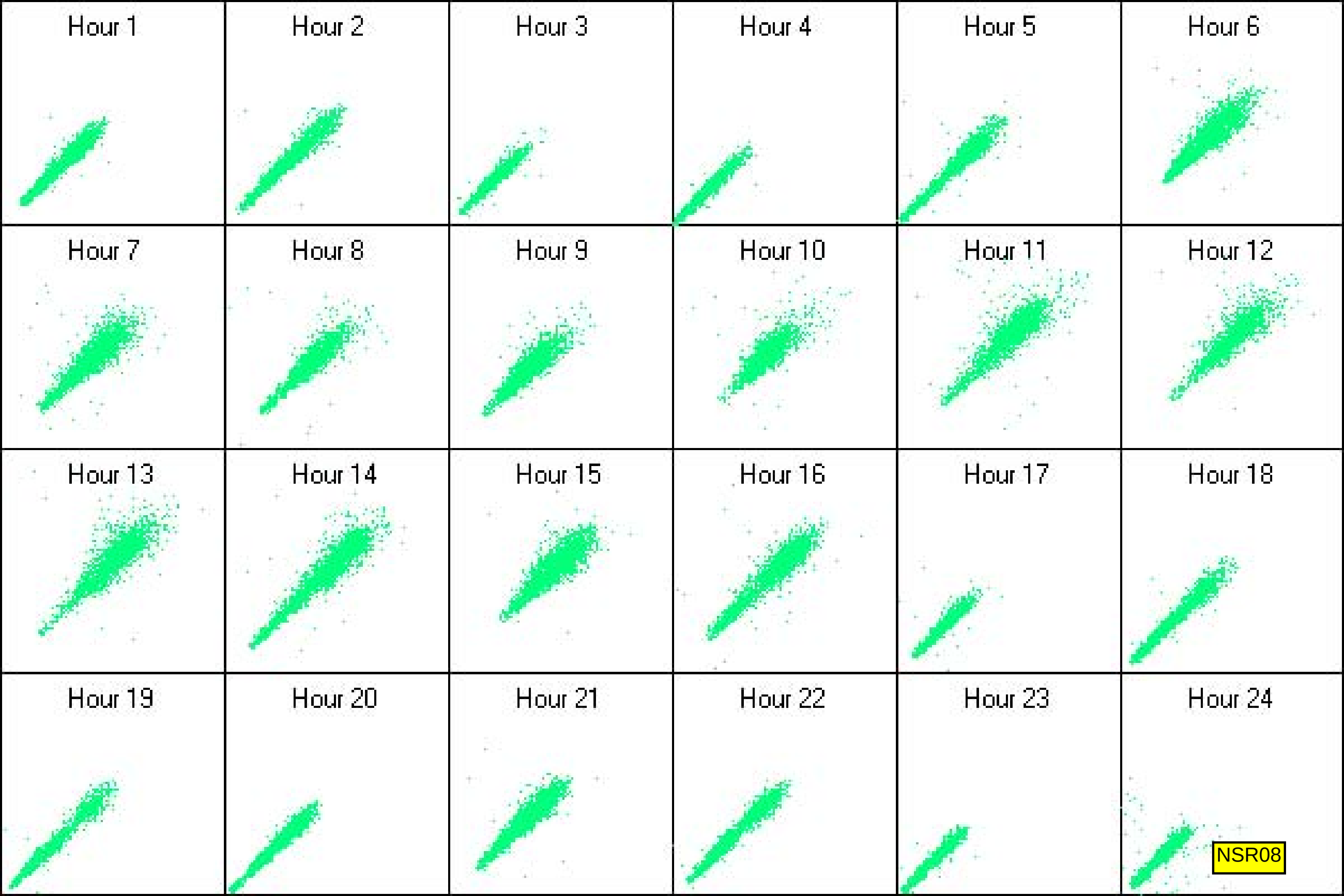
Hour 1
Hour 2
Hour 3
Hour 4
Hour 5
Hour 6
Hour 7
Hour 8
Hour 9
Hour 10
Hour 11
Hour 12
Hour 13
Hour 14
Hour 15
Hour 16
Hour 17
Hour 18
Hour 19
Hour 20
Hour 21
Hour 22
Hour 23
Hour 24
NSR08

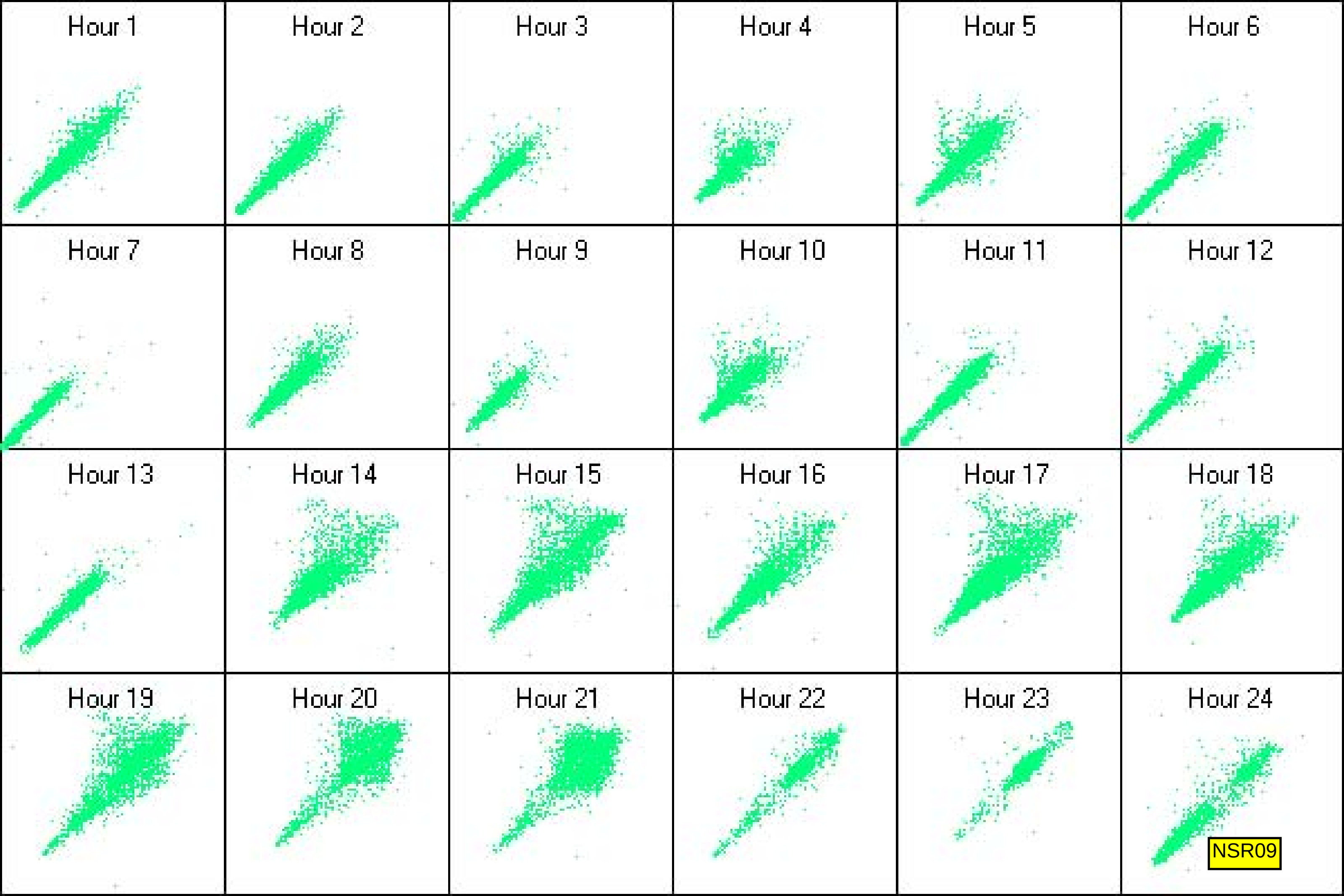
Hour 1
Hour 2
Hour 3
Hour 4
Hour 5
Hour 6
Hour 7
Hour 8
Hour 9
Hour 10
Hour 11
Hour 12
Hour 13
Hour 14
Hour 15
Hour 16
Hour 17
Hour 18
Hour 19
Hour 20
Hour 21
Hour 22
Hour 23
Hour 24
NSR09

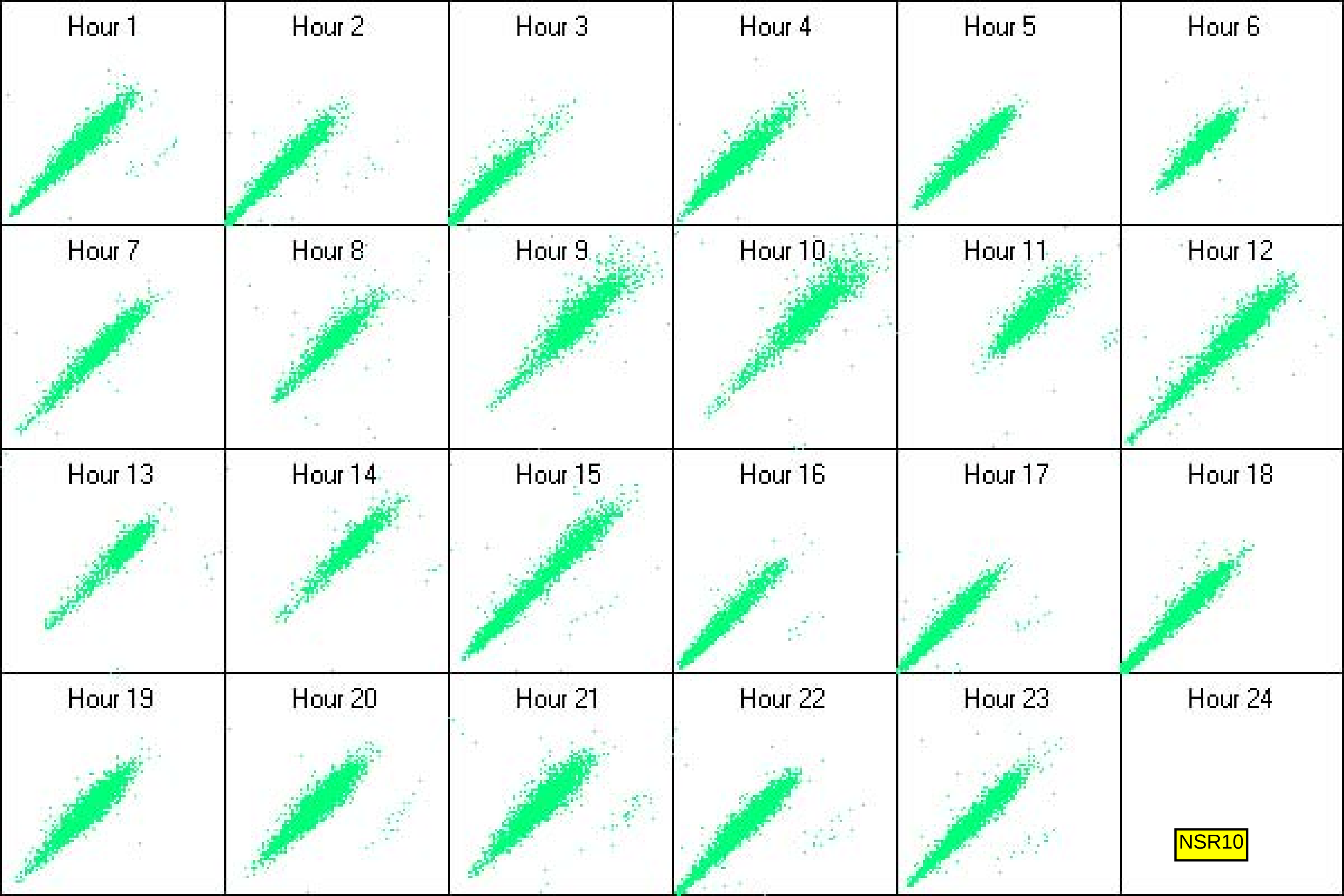
Hour 1
Hour 2
Hour 3
Hour 4
Hour 5
Hour 6
Hour 7
Hour 8
Hour 9
Hour 10
Hour 11
Hour 12
Hour 13
Hour 14
Hour 15
Hour 16
Hour 17
Hour 18
Hour 19
Hour 20
Hour 21
Hour 22
Hour 23
Hour 24
NSR10

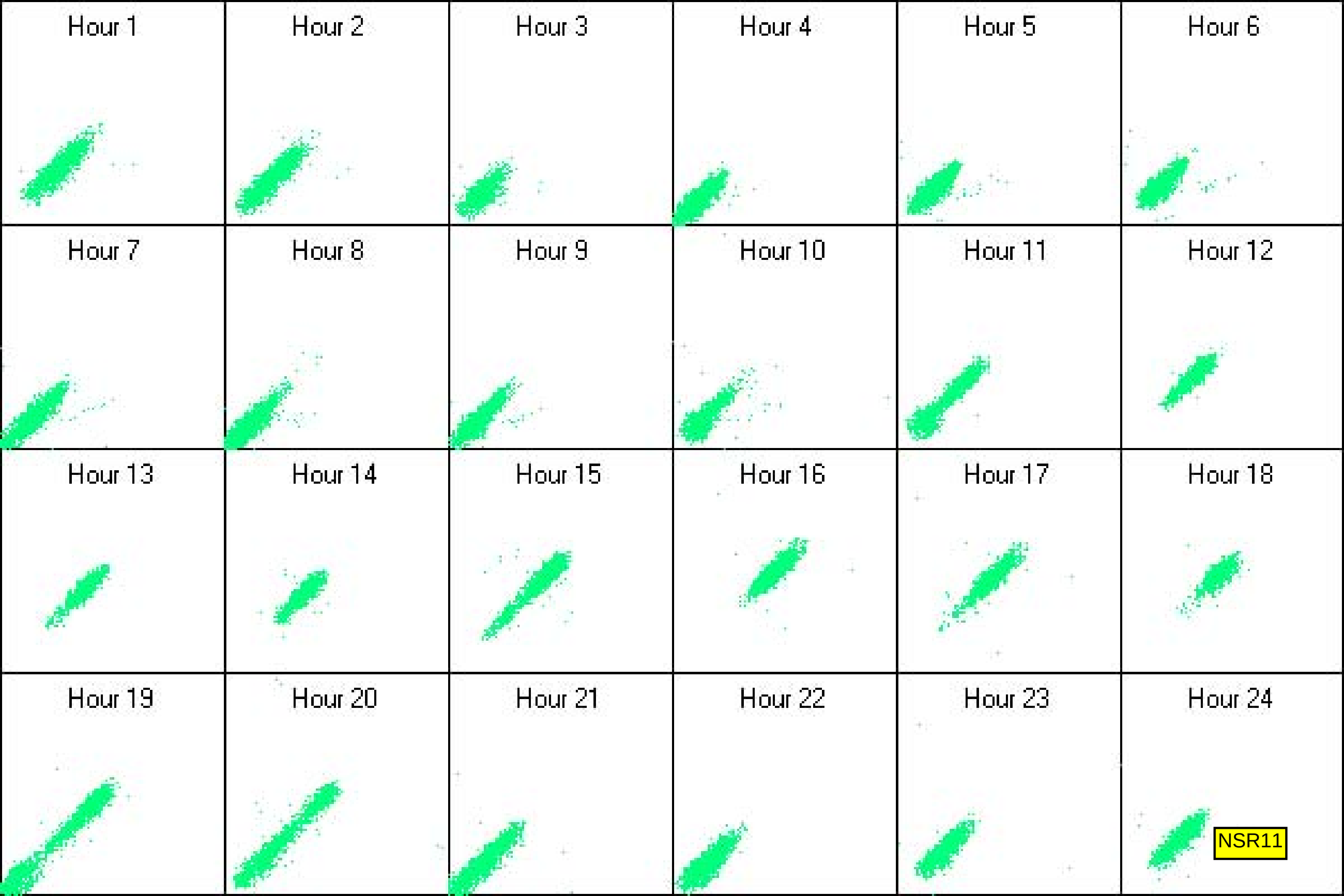
Hour 1
Hour 2
Hour 3
Hour 4
Hour 5
Hour 6
Hour 7
Hour 8
Hour 9
Hour 10
Hour 11
Hour 12
Hour 13
Hour 14
Hour 15
Hour 16
Hour 17
Hour 18
Hour 19
Hour 20
Hour 21
Hour 22
Hour 23
Hour 24
NSR11

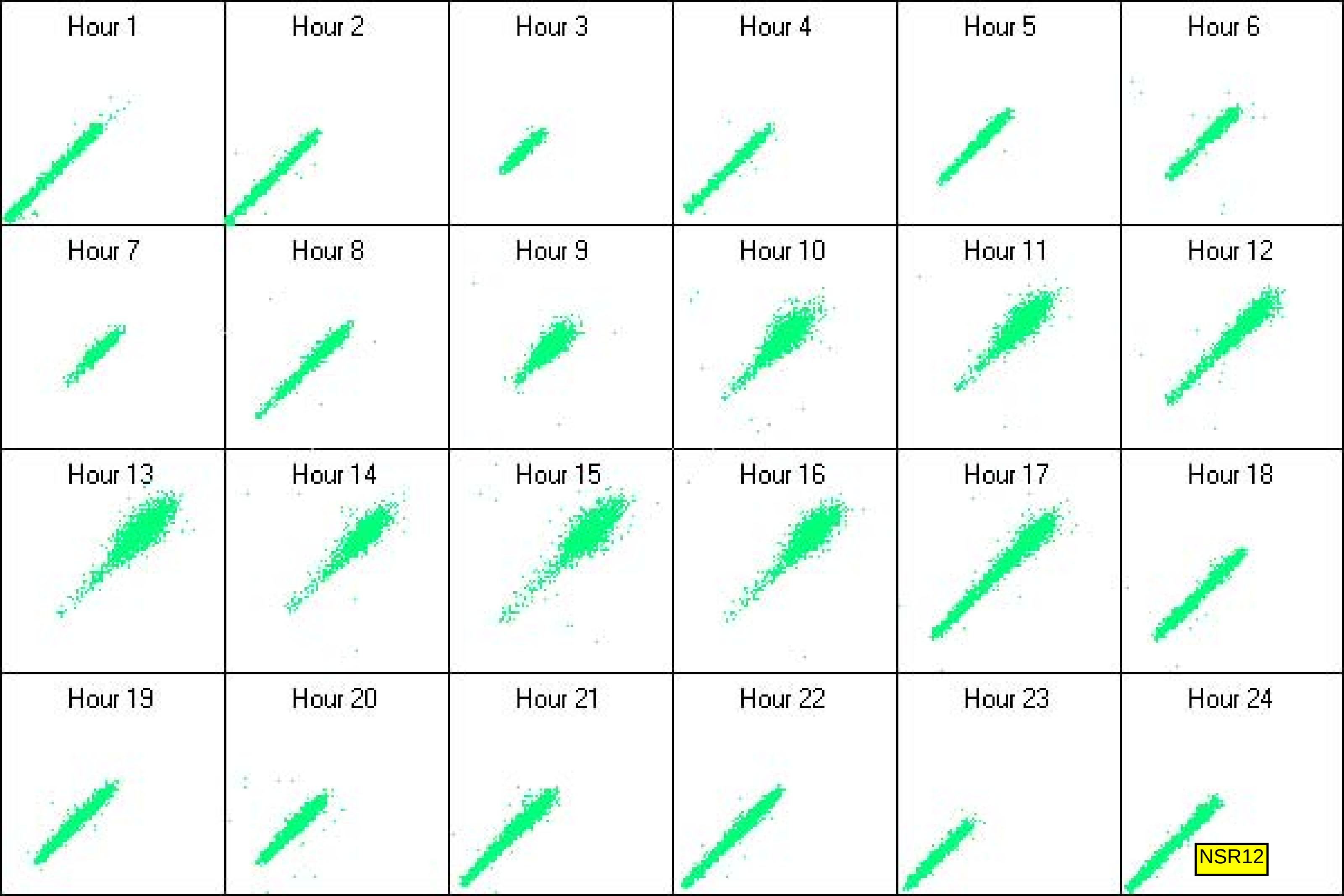
Hour 1
Hour 2
Hour 3
Hour 4
Hour 5
Hour 6
Hour 7
Hour 8
Hour 9
Hour 10
Hour 11
Hour 12
Hour 13
Hour 14
Hour 15
Hour 16
Hour 17
Hour 18
Hour 19
Hour 20
Hour 21
Hour 22
Hour 23
Hour 24
NSR12

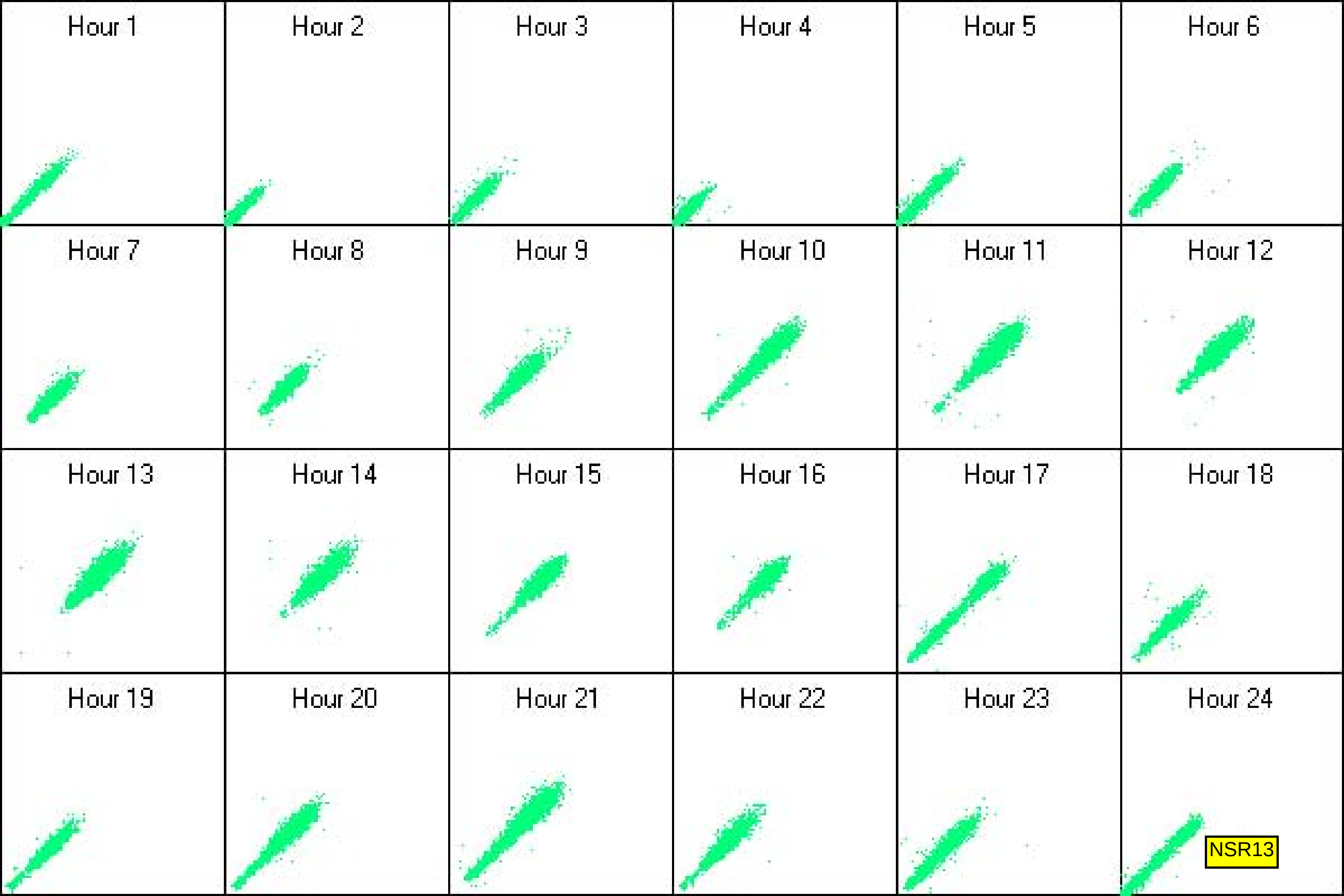

Hour 1
Hour 2
Hour 3
Hour 4
Hour 5
Hour 6
Hour 7
Hour 8
Hour 9
Hour 10
Hour 11
Hour 12
Hour 13
Hour 14
Hour 15
Hour 16
Hour 17
Hour 18
Hour 19
Hour 20
Hour 21
Hour 22
Hour 23
Hour 24
NSR13

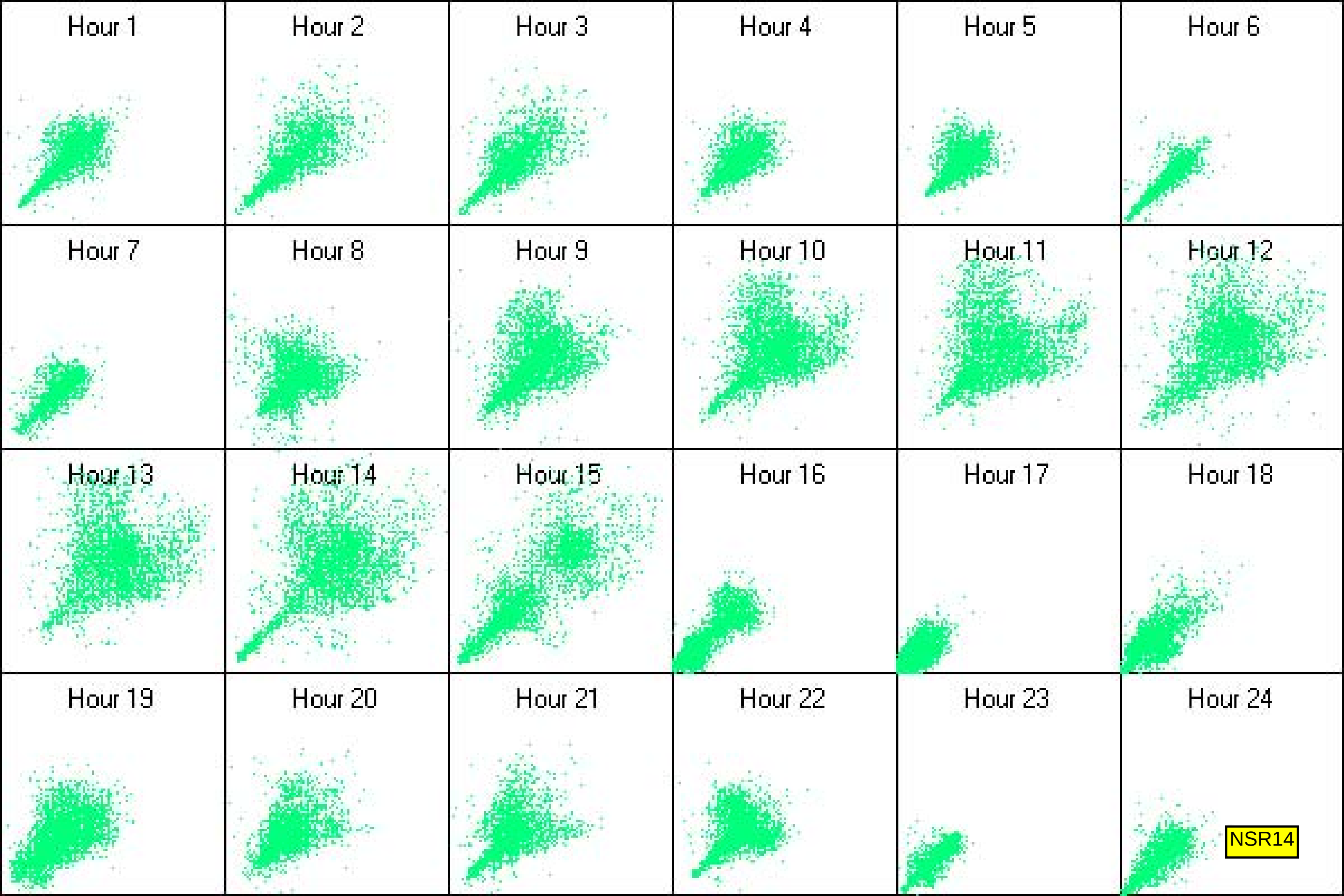
Hour 1
Hour 2
Hour 3
Hour 4
Hour 5
Hour 6
Hour 7
Hour 8
Hour 9
Hour 10
Hour 11
Hour 12
Hour 13
Hour 14
Hour 15
Hour 16
Hour 17
Hour 18
Hour 19
Hour 20
Hour 21
Hour 22
Hour 23
Hour 24
NSR14

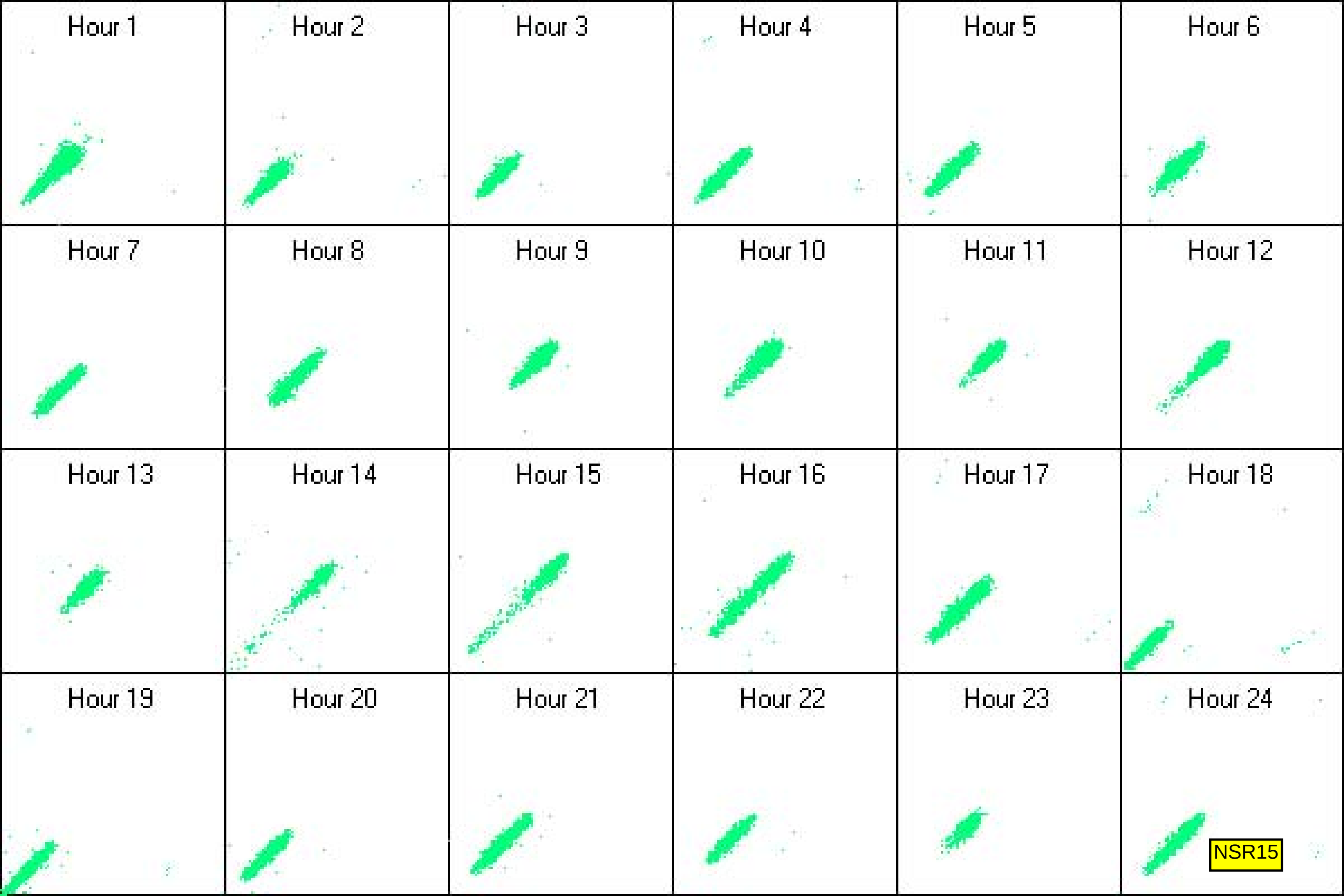
Hour 1
Hour 2
Hour 3
Hour 4
Hour 5
Hour 6
Hour 7
Hour 8
Hour 9
Hour 10
Hour 11
Hour 12
Hour 13
Hour 14
Hour 15
Hour 16
Hour 17
Hour 18
Hour 19
Hour 20
Hour 21
Hour 22
Hour 23
Hour 24
NSR15

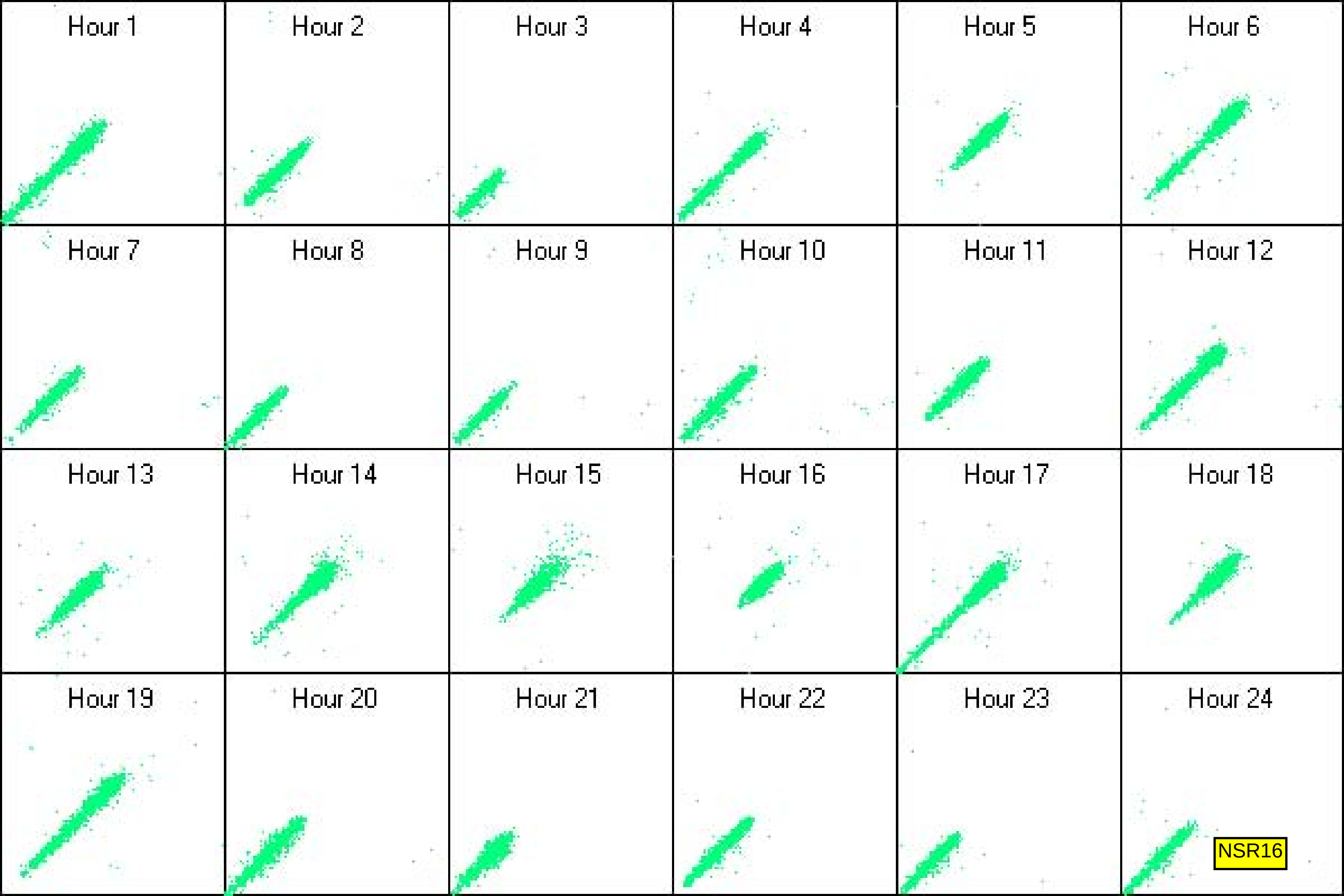
Hour 1
Hour 2
Hour 3
Hour 4
Hour 5
Hour 6
Hour 7
Hour 8
Hour 9
Hour 10
Hour 11
Hour 12
Hour 13
Hour 14
Hour 15
Hour 16
Hour 17
Hour 18
Hour 19
Hour 20
Hour 21
Hour 22
Hour 23
Hour 24
NSR16

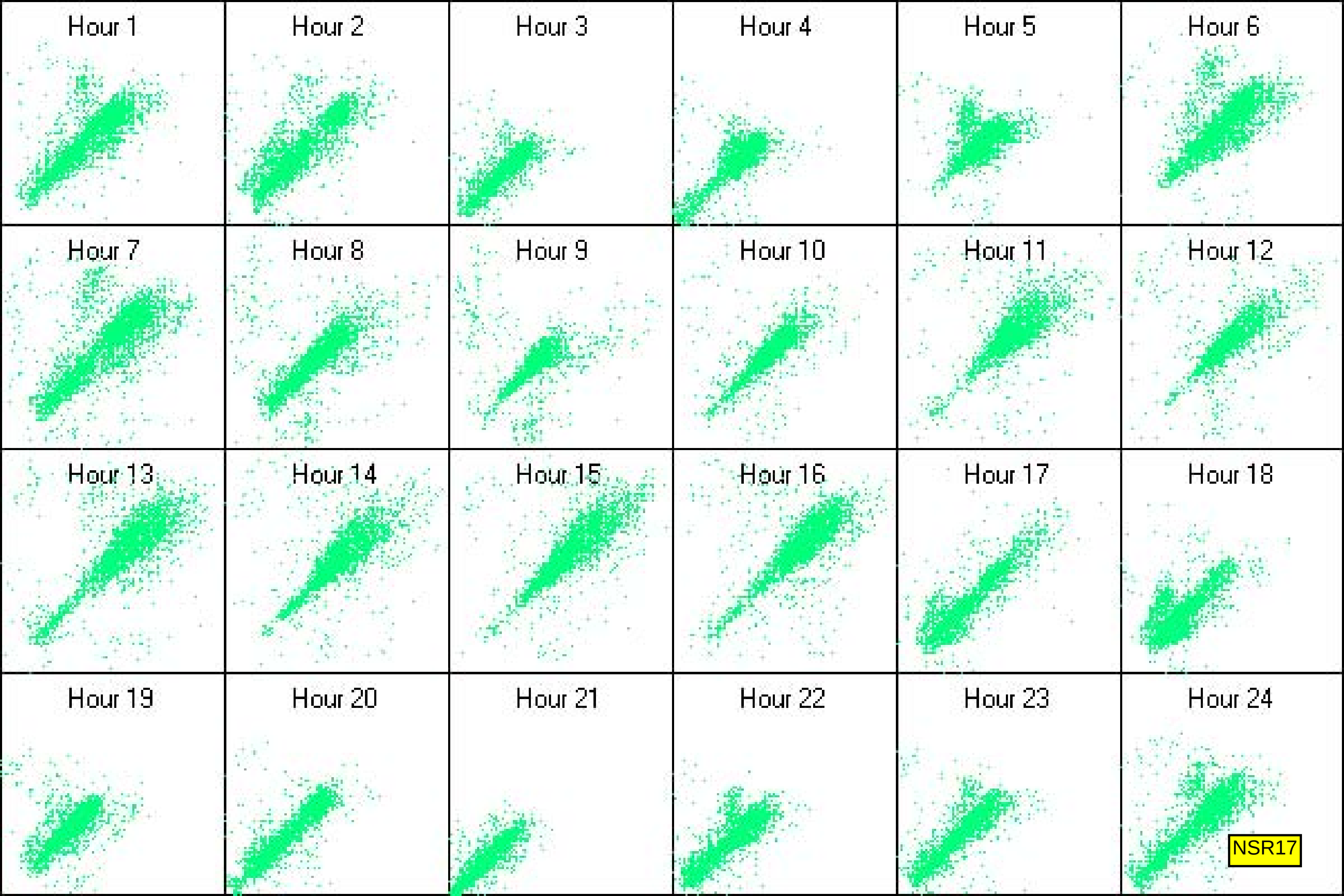
Hour 1
Hour 2
Hour 3
Hour 4
Hour 5
Hour 6
Hour 7
Hour 8
Hour 9
Hour 10
Hour 11
Hour 12
Hour 13
Hour 14
Hour 15
Hour 16
Hour 17
Hour 18
Hour 19
Hour 20
Hour 21
Hour 22
Hour 23
Hour 24
NSR17

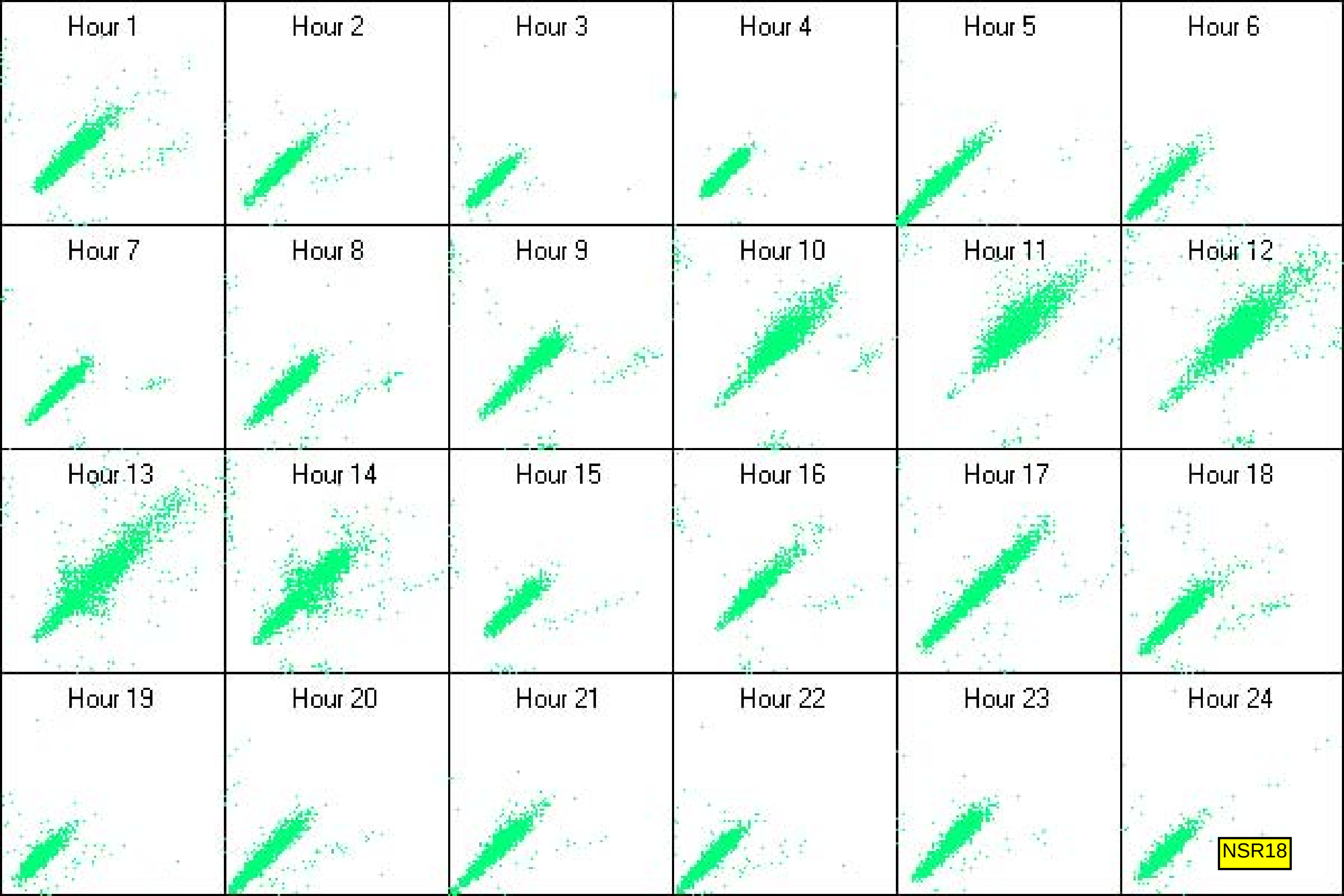
Hour 1
Hour 2
Hour 3
Hour 4
Hour 5
Hour 6
Hour 7
Hour 8
Hour 9
Hour 10
Hour 11
Hour 12
Hour 13
Hour 14
Hour 15
Hour 16
Hour 17
Hour 18
Hour 19
Hour 20
Hour 21
Hour 22
Hour 23
Hour 24
NSR18

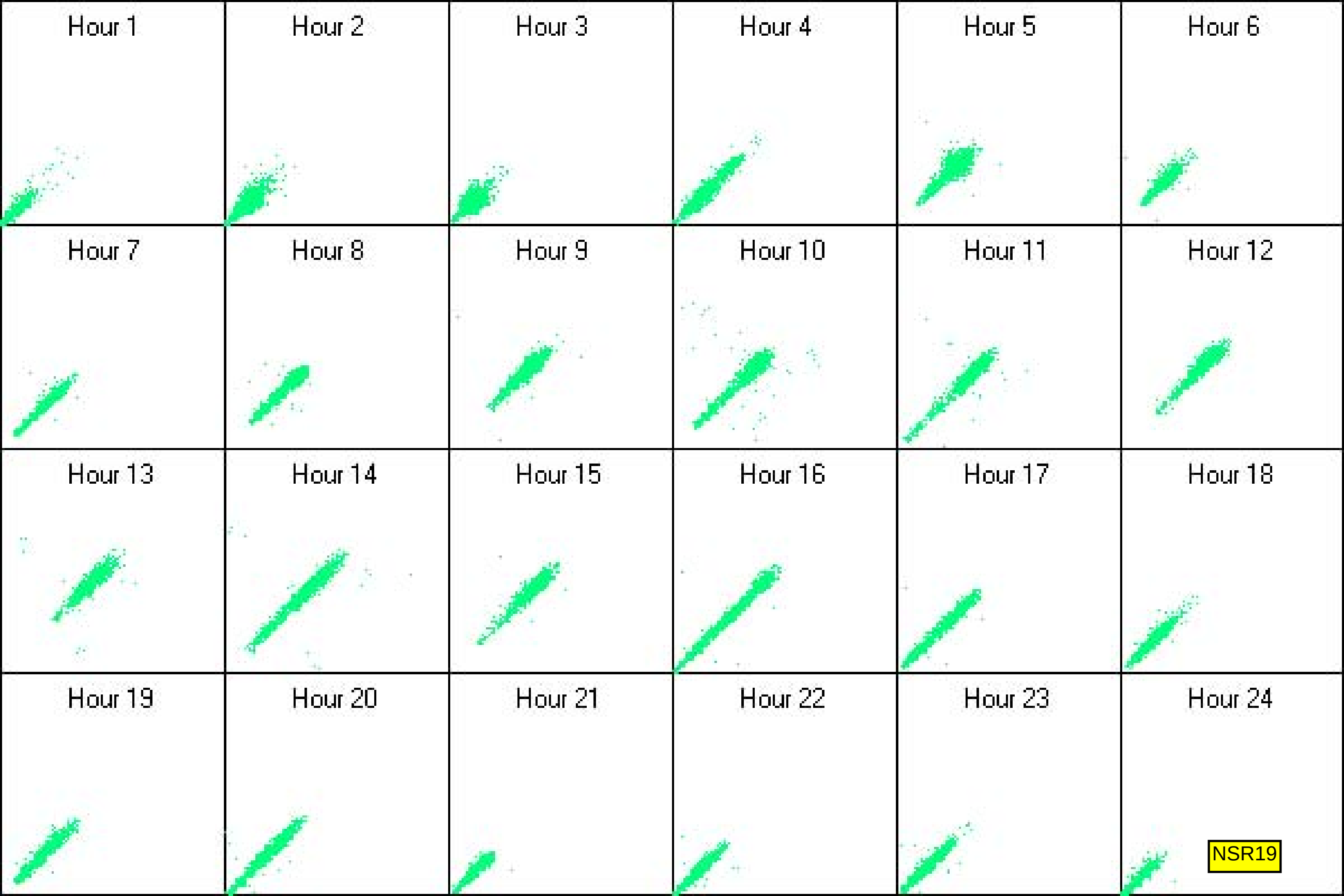
Hour 1
Hour 2
Hour 3
Hour 4
Hour 5
Hour 6
Hour 7
Hour 8
Hour 9
Hour 10
Hour 11
Hour 12
Hour 13
Hour 14
Hour 15
Hour 16
Hour 17
Hour 18
Hour 19
Hour 20
Hour 21
Hour 22
Hour 23
Hour 24
NSR19

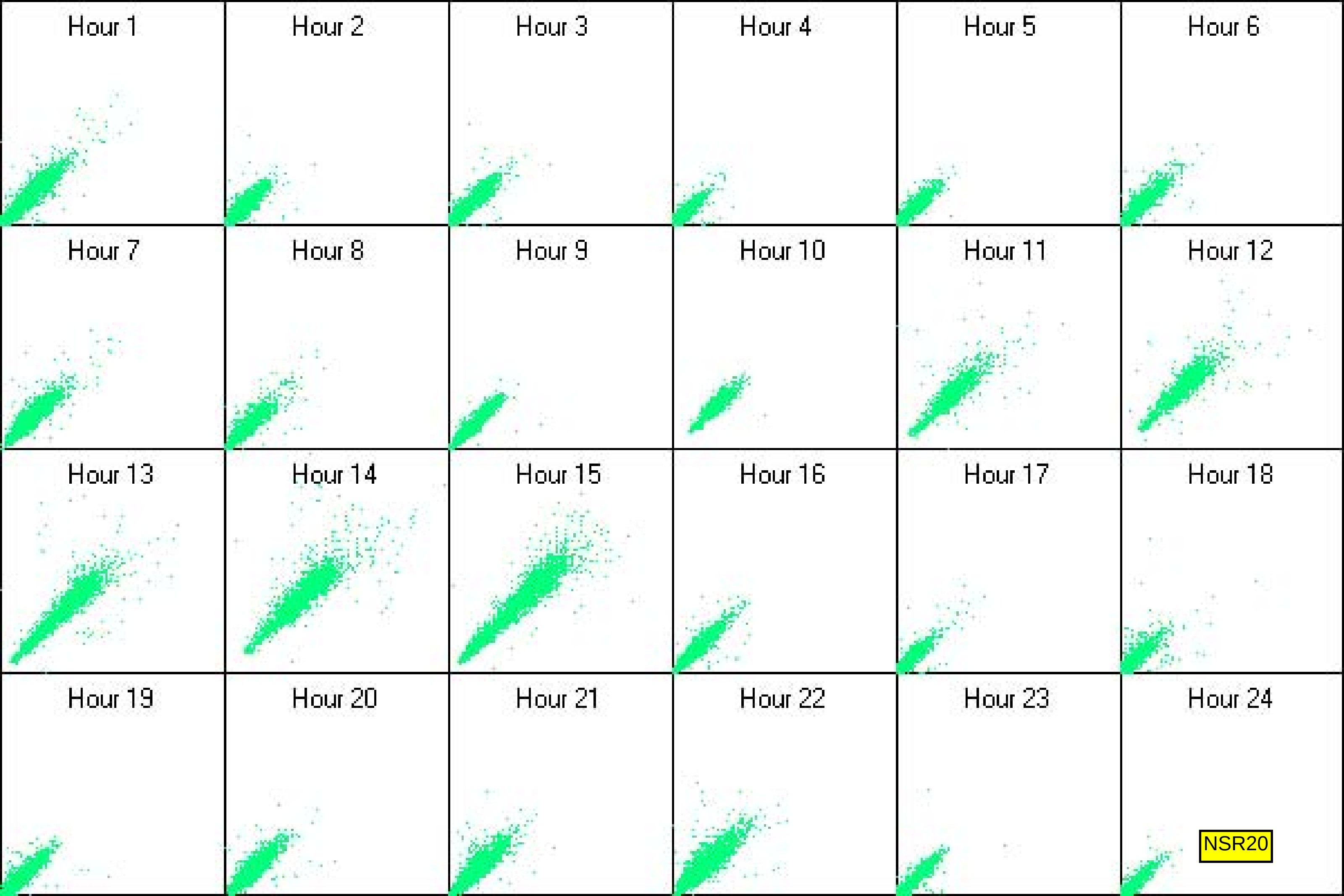

Hour 1
Hour 2
Hour 3
Hour 4
Hour 5
Hour 6
Hour 7
Hour 8
Hour 9
Hour 10
Hour 11
Hour 12
Hour 13
Hour 14
Hour 15
Hour 16
Hour 17
Hour 18
Hour 19
Hour 20
Hour 21
Hour 22
Hour 23
Hour 24
NSR20

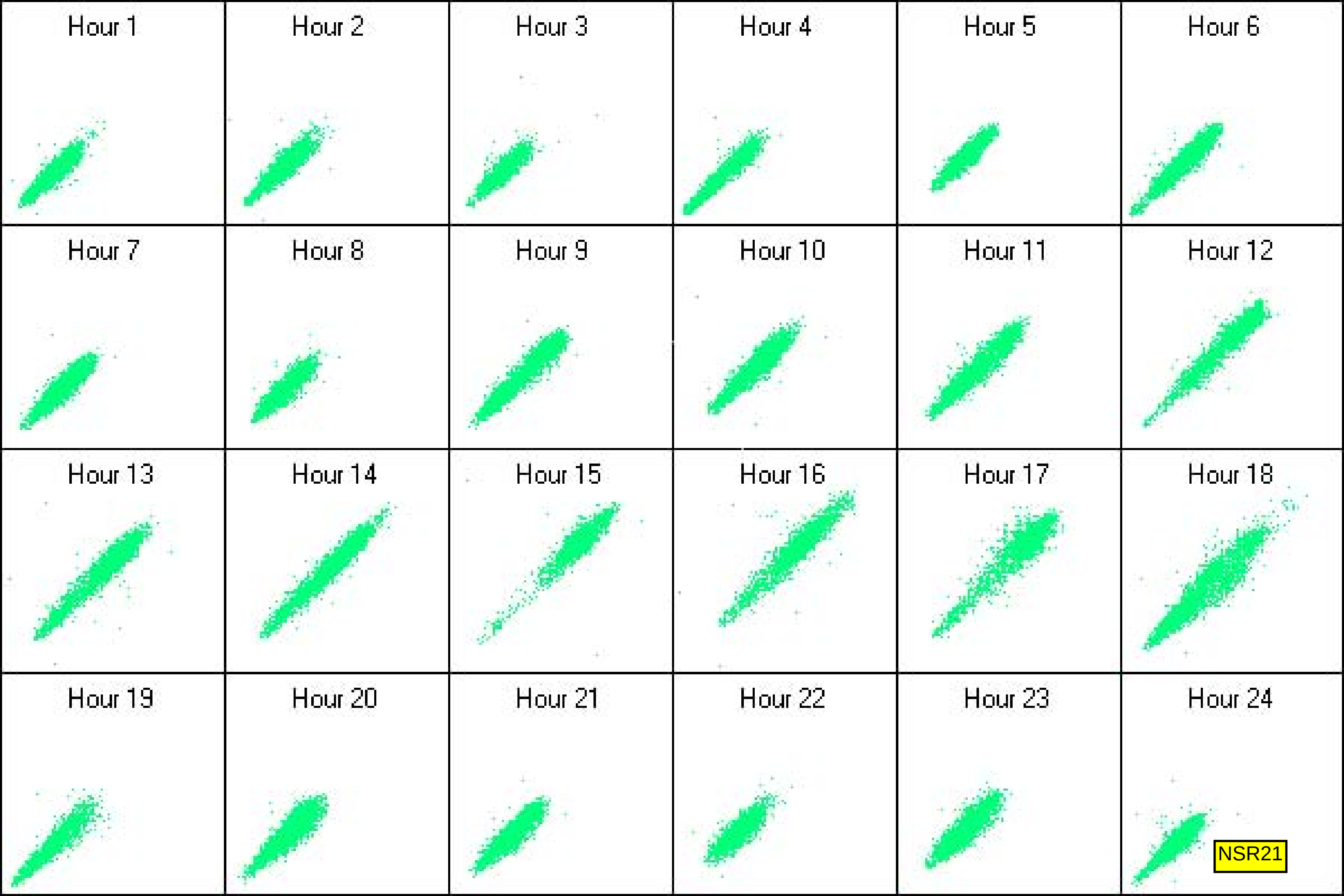
Hour 1
Hour 2
Hour 3
Hour 4
Hour 5
Hour 6
Hour 7
Hour 8
Hour 9
Hour 10
Hour 11
Hour 12
Hour 13
Hour 14
Hour 15
Hour 16
Hour 17
Hour 18
Hour 19
Hour 20
Hour 21
Hour 22
Hour 23
Hour 24
NSR21

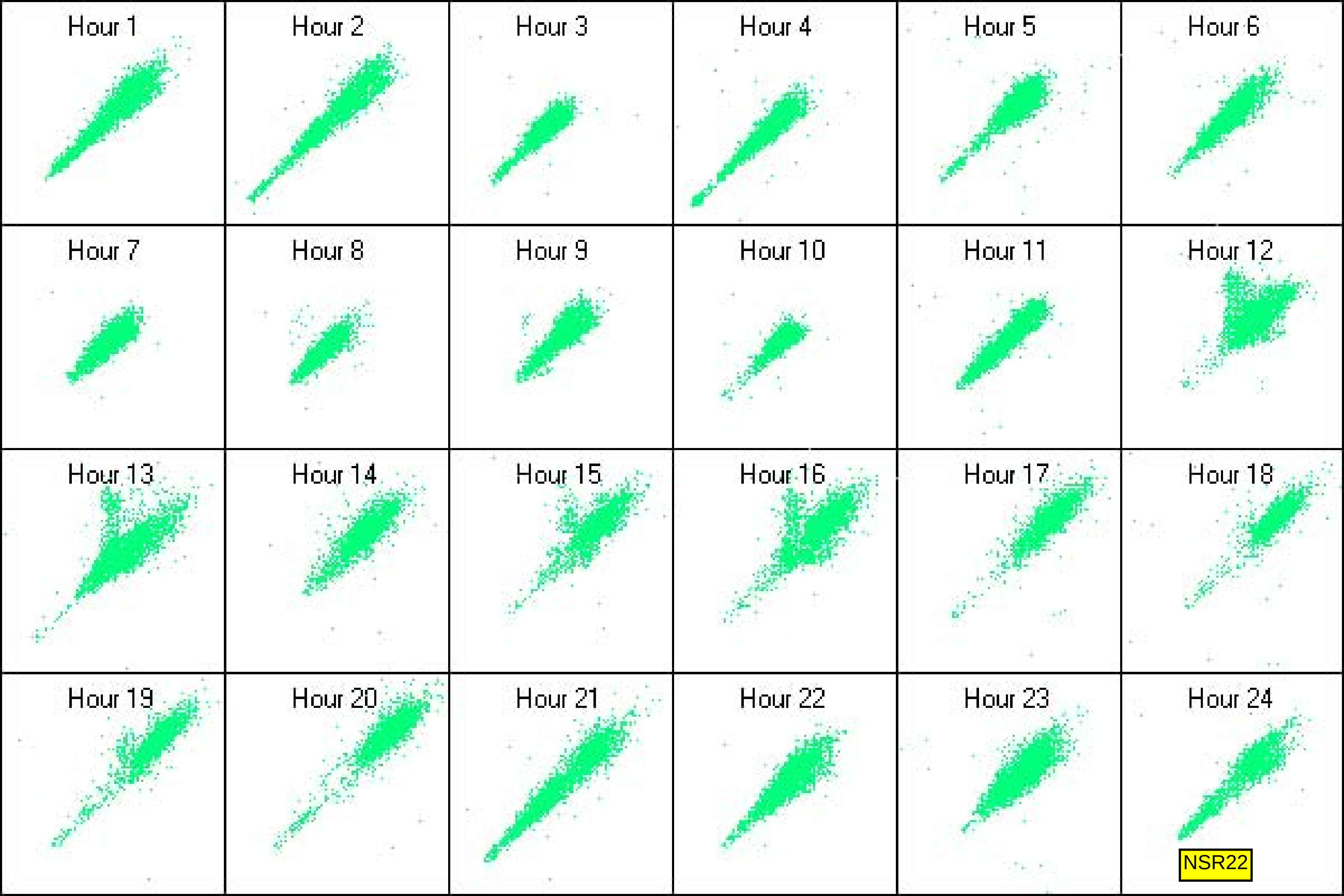
Hour 1
Hour 2
Hour 3
Hour 4
Hour 5
Hour 6
Hour 7
Hour 8
Hour 9
Hour 10
Hour 11
Hour 12
Hour 13
Hour 14
Hour 15
Hour 16
Hour 17
Hour 18
Hour 19
Hour 20
Hour 21
Hour 22
Hour 23
Hour 24
NSR22

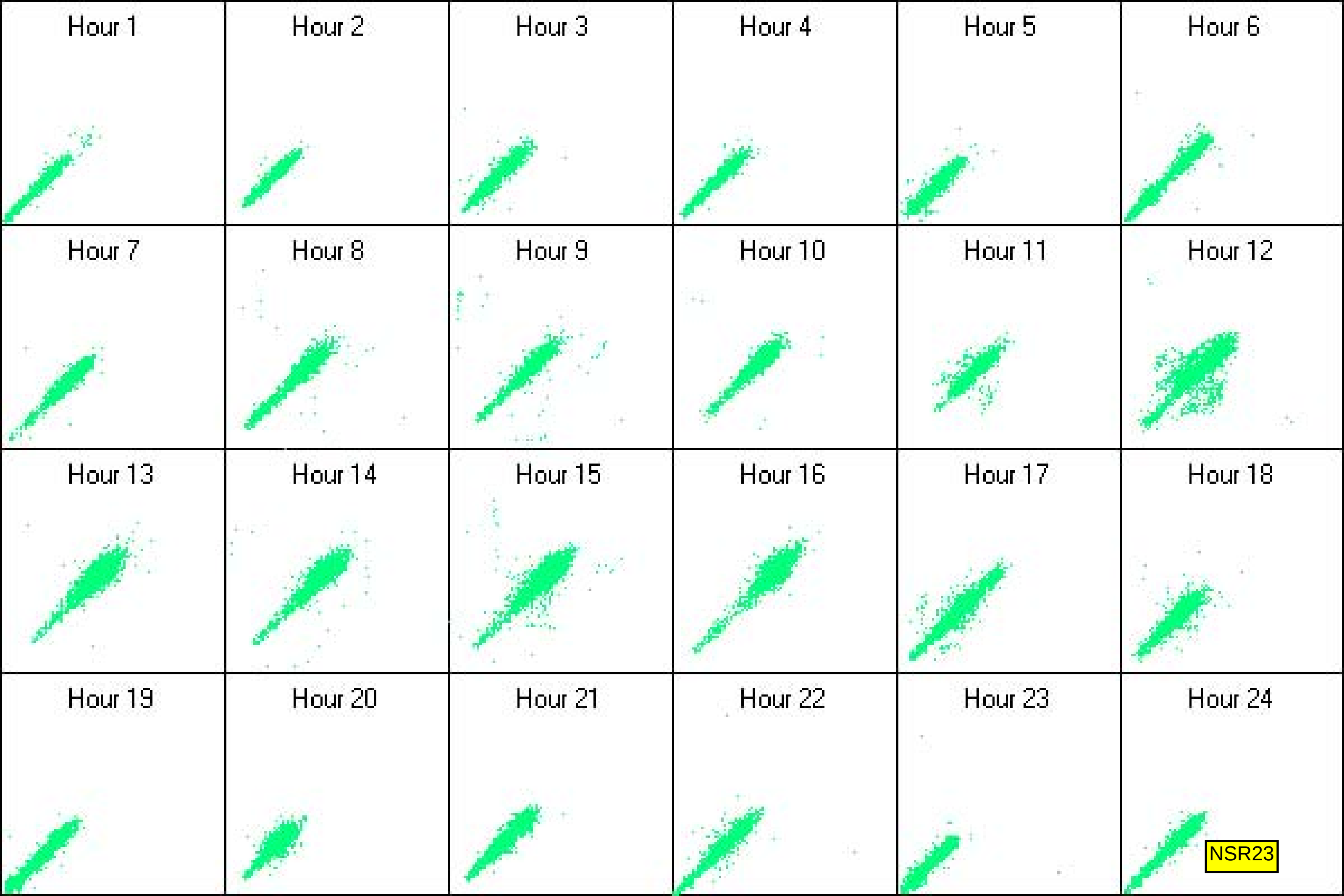
Hour 1
Hour 2
Hour 3
Hour 4
Hour 5
Hour 6
Hour 7
Hour 8
Hour 9
Hour 10
Hour 11
Hour 12
Hour 13
Hour 14
Hour 15
Hour 16
Hour 17
Hour 18
Hour 19
Hour 20
Hour 21
Hour 22
Hour 23
Hour 24
NSR23

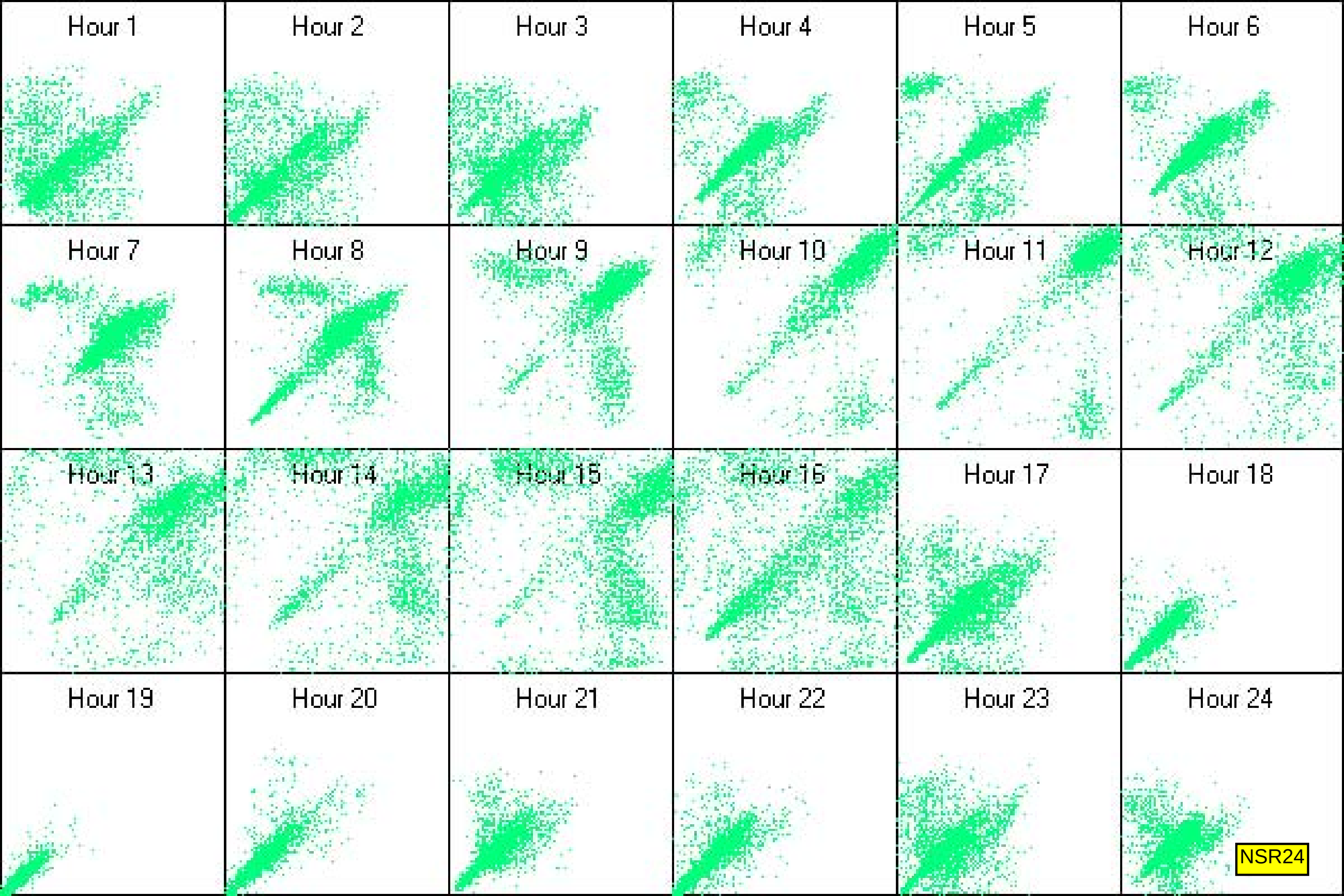
Hour 1
Hour 2
Hour 3
Hour 4
Hour 5
Hour 6
Hour 7
Hour 8
Hour 9
Hour 10
Hour 11
Hour 12
Hour 13
Hour 14
Hour 15
Hour 16
Hour 17
Hour 18
Hour 19
Hour 20
Hour 21
Hour 22
Hour 23
Hour 24
NSR24

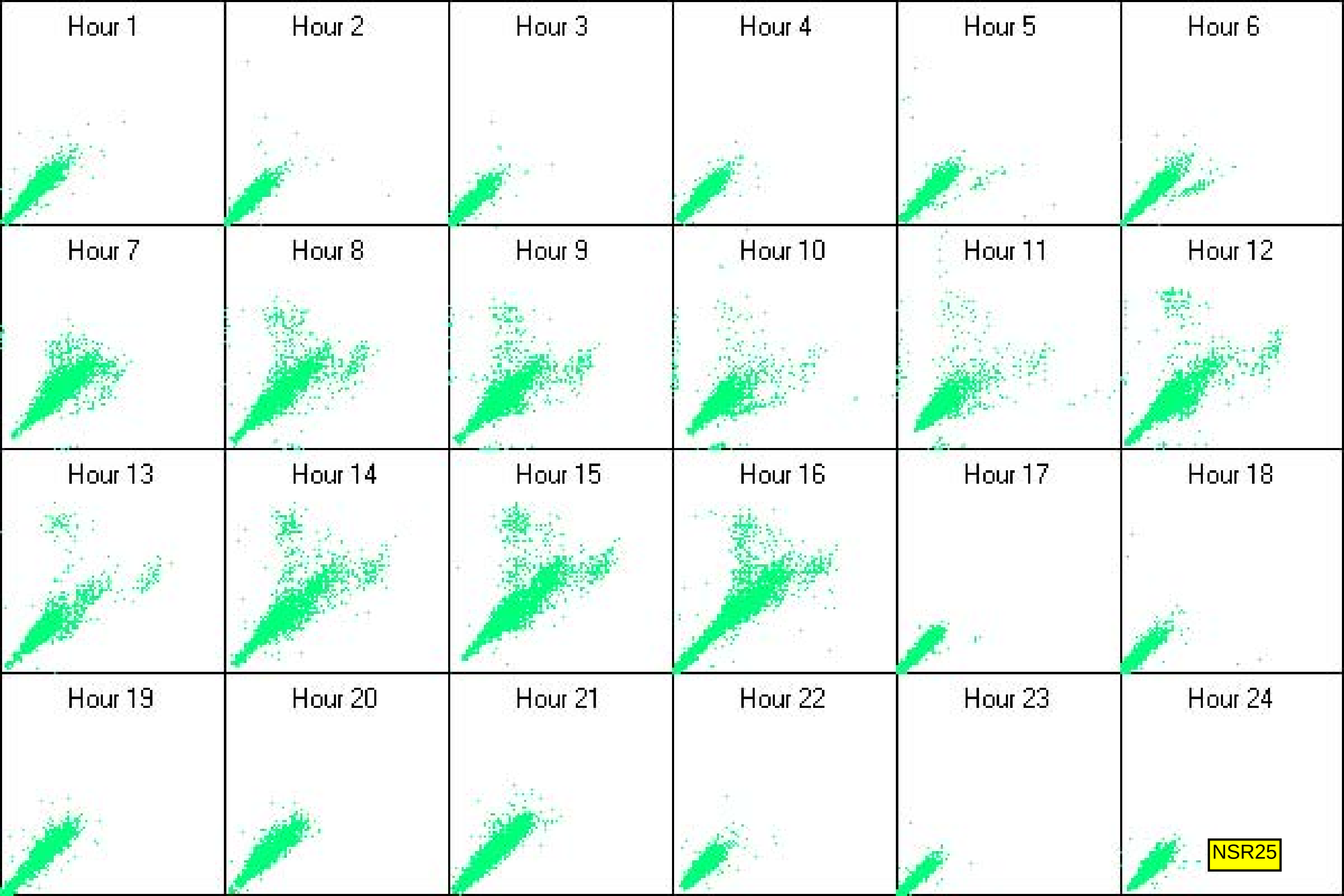
Hour 1
Hour 2
Hour 3
Hour 4
Hour 5
Hour 6
Hour 7
Hour 8
Hour 9
Hour 10
Hour 11
Hour 12
Hour 13
Hour 14
Hour 15
Hour 16
Hour 17
Hour 18
Hour 19
Hour 20
Hour 21
Hour 22
Hour 23
Hour 24
NSR25

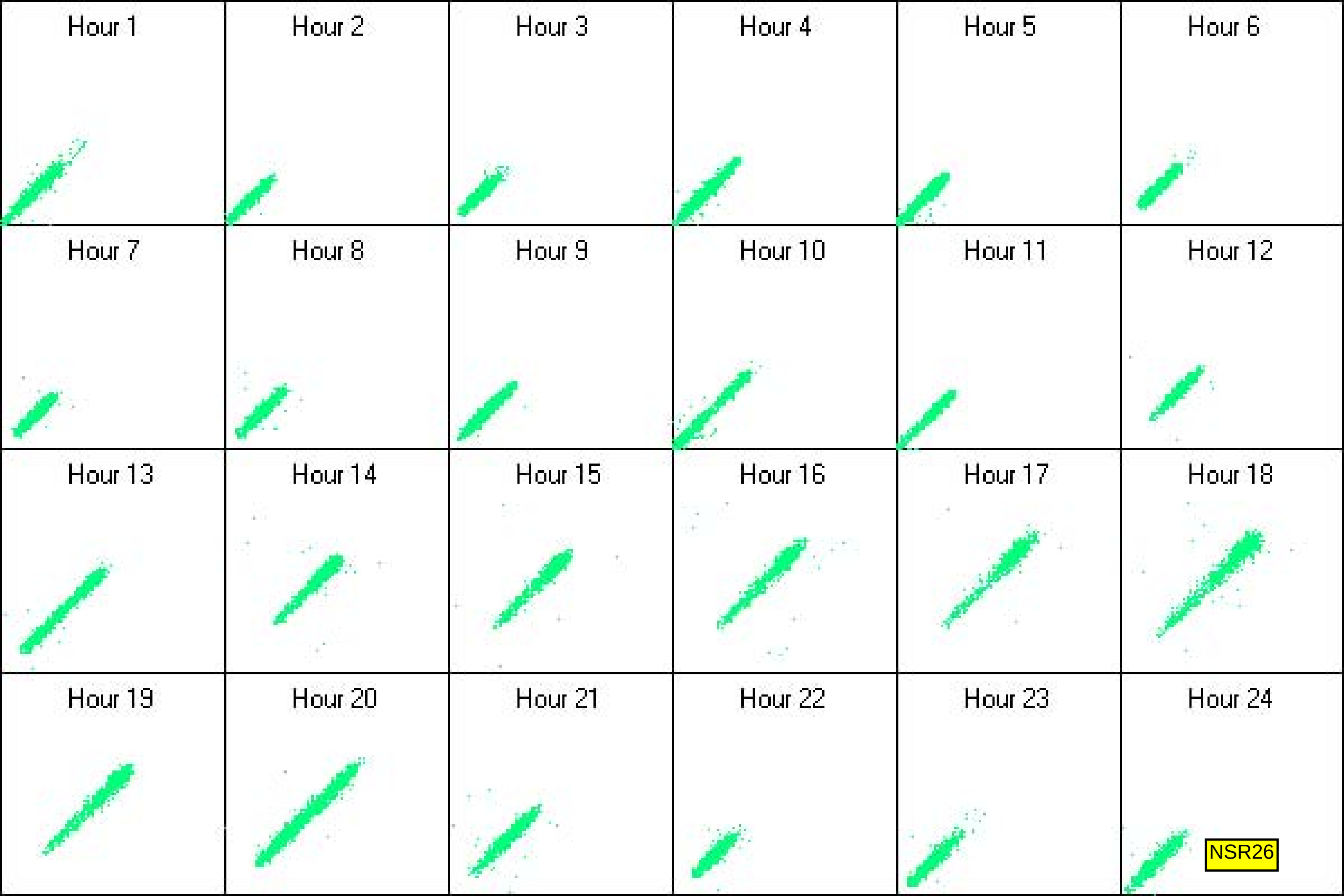
Hour 1
Hour 2
Hour 3
Hour 4
Hour 5
Hour 6
Hour 7
Hour 8
Hour 9
Hour 10
Hour 11
Hour 12
Hour 13
Hour 14
Hour 15
Hour 16
Hour 17
Hour 18
Hour 19
Hour 20
Hour 21
Hour 22
Hour 23
Hour 24
NSR26

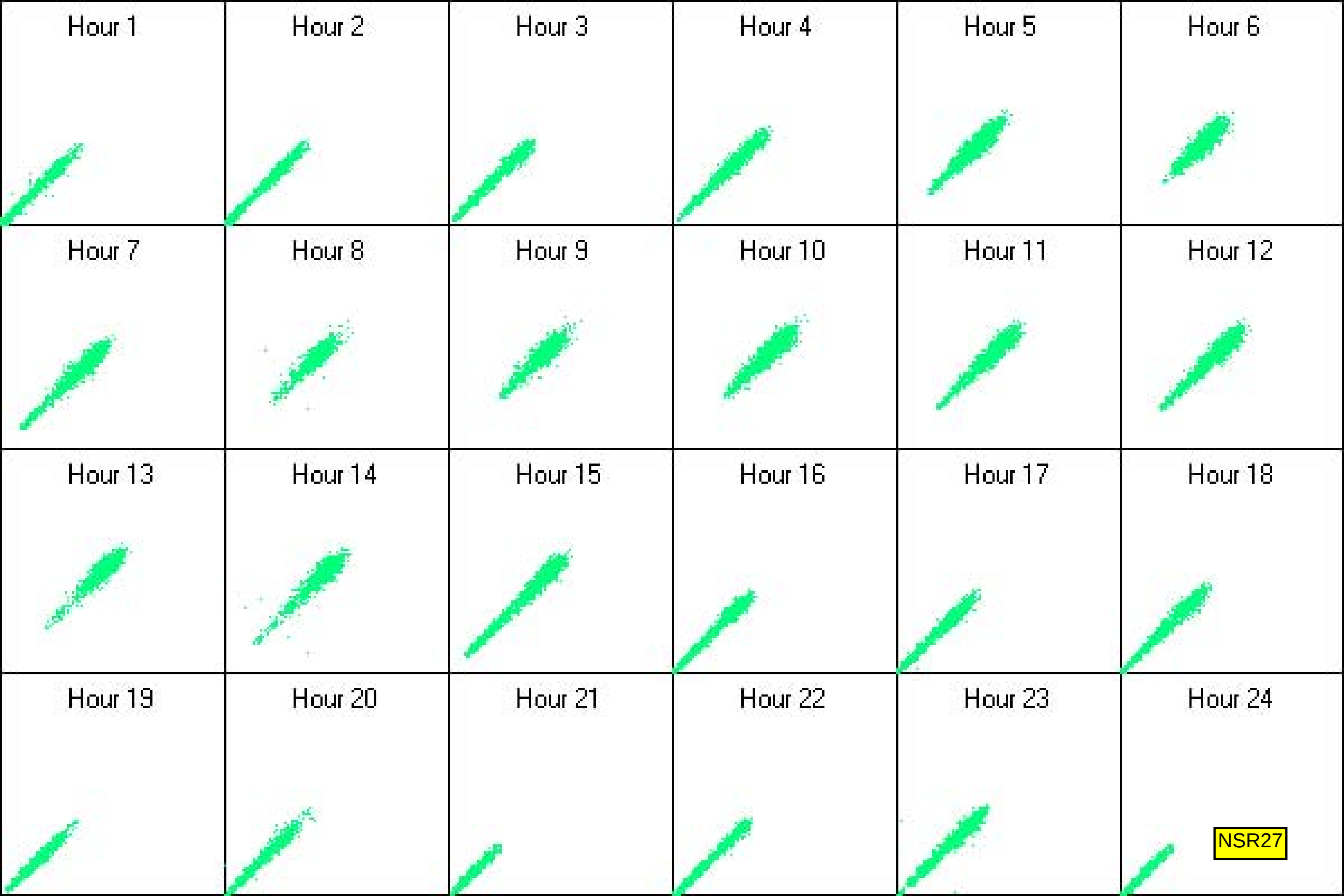
Hour 1
Hour 2
Hour 3
Hour 4
Hour 5
Hour 6
Hour 7
Hour 8
Hour 9
Hour 10
Hour 11
Hour 12
Hour 13
Hour 14
Hour 15
Hour 16
Hour 17
Hour 18
Hour 19
Hour 20
Hour 21
Hour 22
Hour 23
Hour 24
NSR27

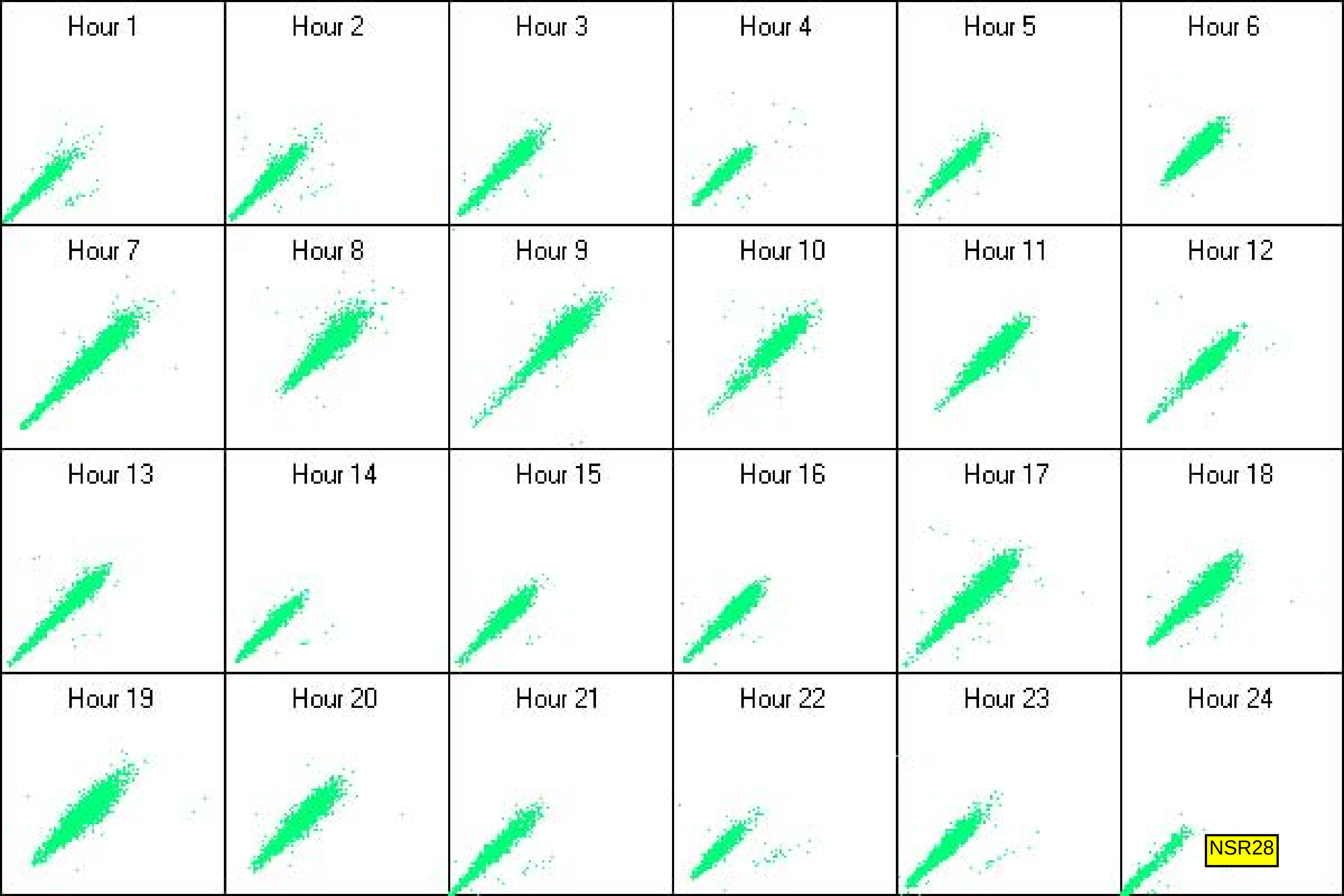

Hour 1
Hour 2
Hour 3
Hour 4
Hour 5
Hour 6
Hour 7
Hour 8
Hour 9
Hour 10
Hour 11
Hour 12
Hour 13
Hour 14
Hour 15
Hour 16
Hour 17
Hour 18
Hour 19
Hour 20
Hour 21
Hour 22
Hour 23
Hour 24
NSR28

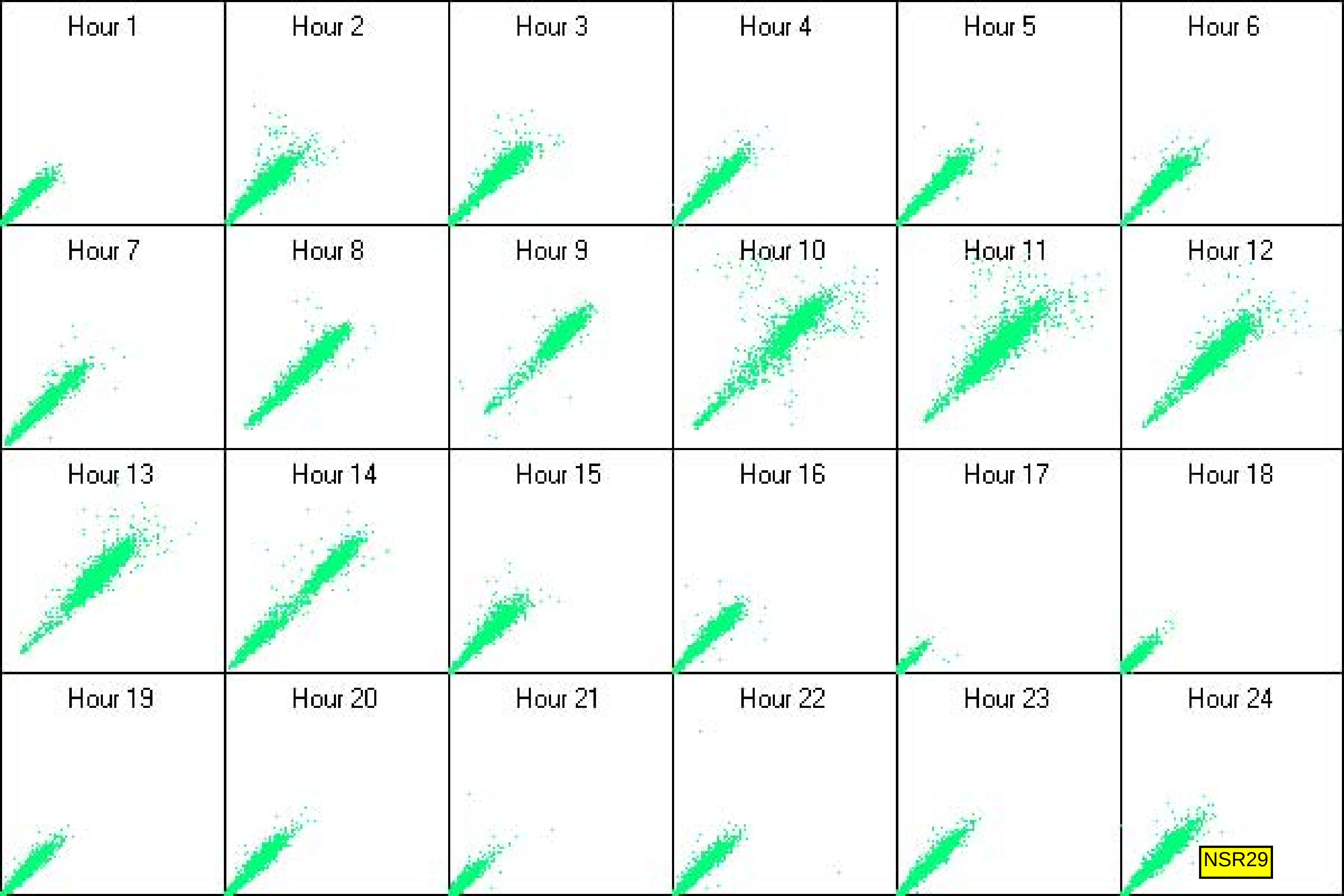
Hour 1
Hour 2
Hour 3
Hour 4
Hour 5
Hour 6
Hour 7
Hour 8
Hour 9
Hour 10
Hour 11
Hour 12
Hour 13
Hour 14
Hour 15
Hour 16
Hour 17
Hour 18
Hour 19
Hour 20
Hour 21
Hour 22
Hour 23
Hour 24
NSR29

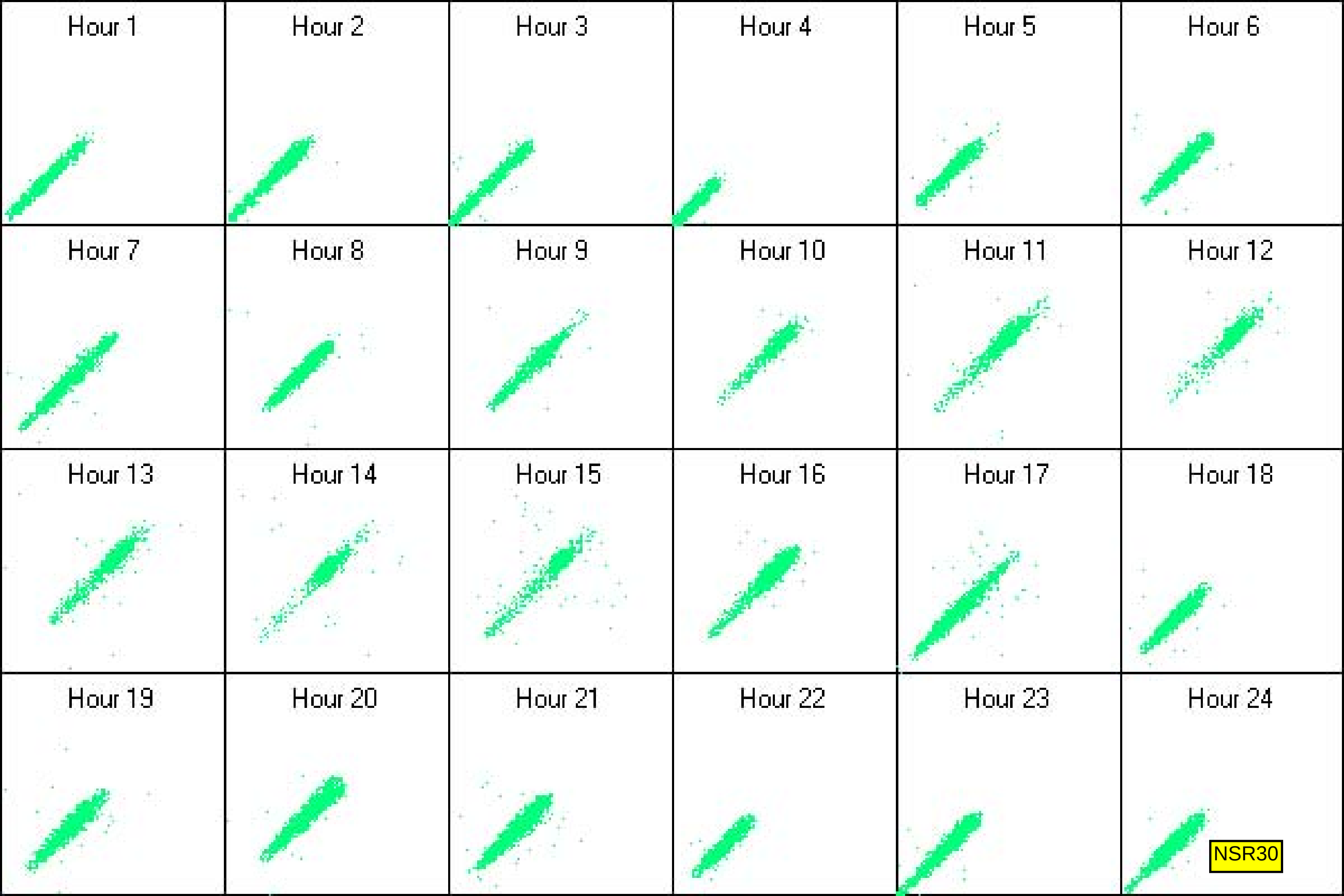
Hour 1
Hour 2
Hour 3
Hour 4
Hour 5
Hour 6
Hour 7
Hour 8
Hour 9
Hour 10
Hour 11
Hour 12
Hour 13
Hour 14
Hour 15
Hour 16
Hour 17
Hour 18
Hour 19
Hour 20
Hour 21
Hour 22
Hour 23
Hour 24
NSR30

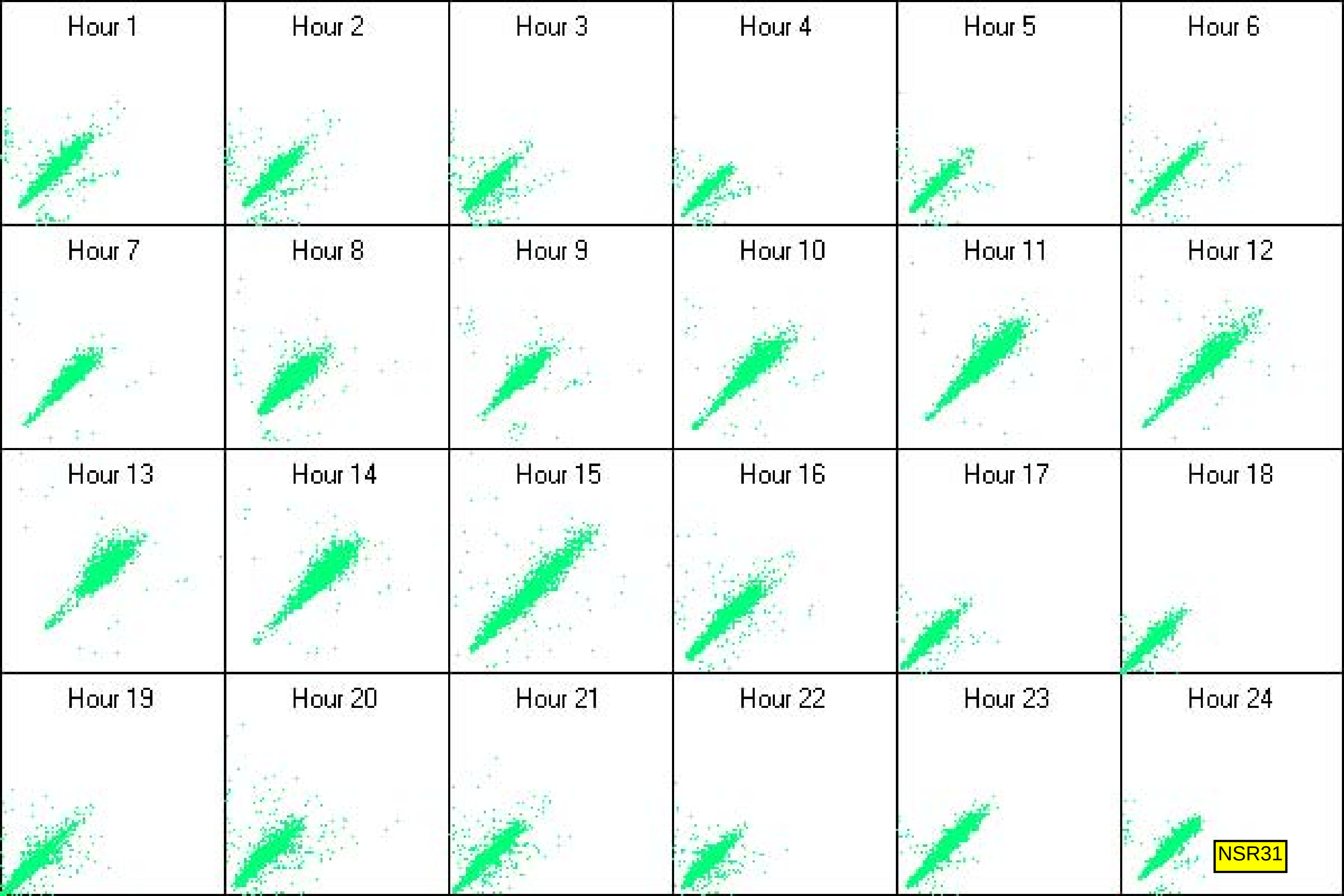
Hour 1
Hour 2
Hour 3
Hour 4
Hour 5
Hour 6
Hour 7
Hour 8
Hour 9
Hour 10
Hour 11
Hour 12
Hour 13
Hour 14
Hour 15
Hour 16
Hour 17
Hour 18
Hour 19
Hour 20
Hour 21
Hour 22
Hour 23
Hour 24
NSR31

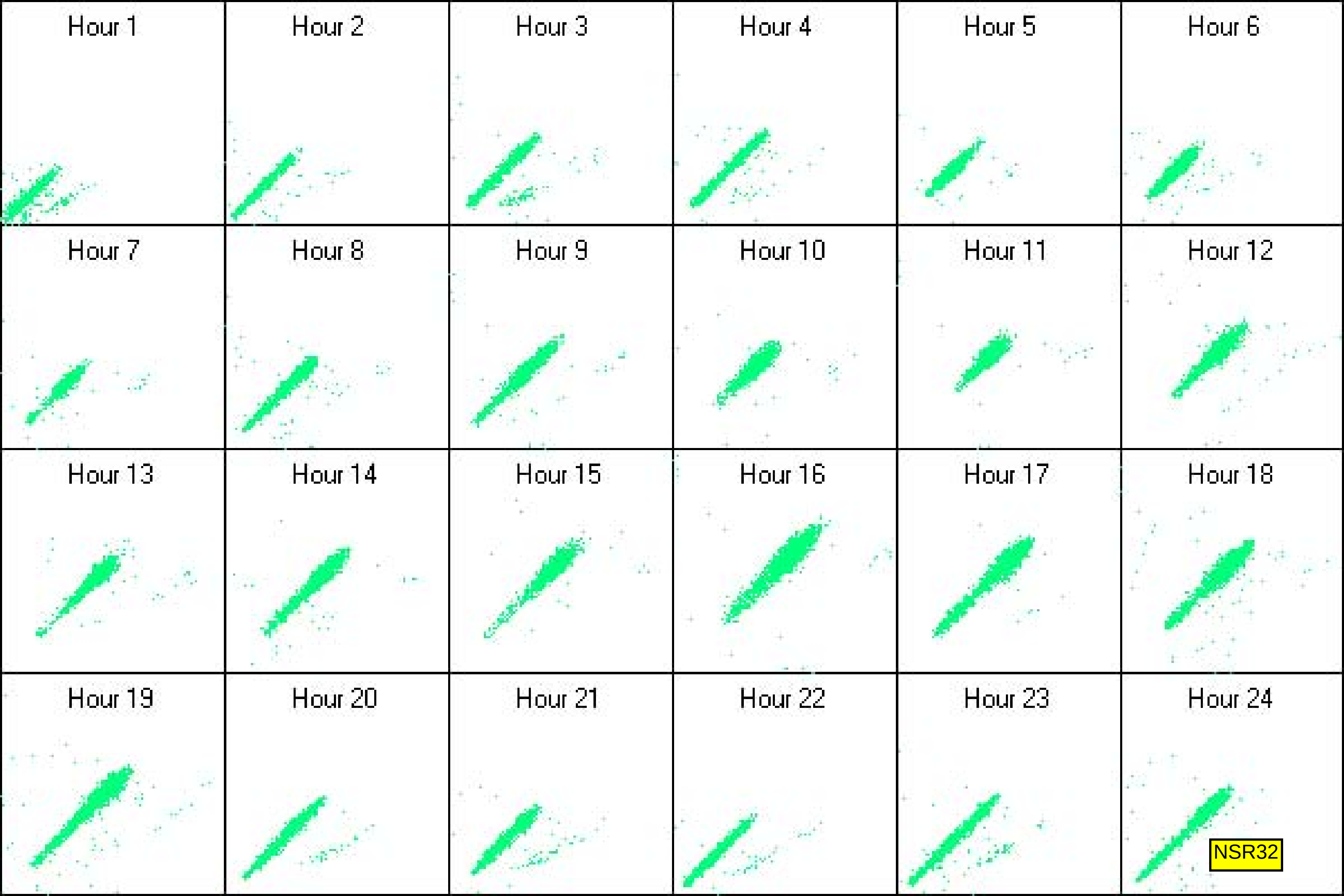

Hour 1
Hour 2
Hour 3
Hour 4
Hour 5
Hour 6
Hour 7
Hour 8
Hour 9
Hour 10
Hour 11
Hour 12
Hour 13
Hour 14
Hour 15
Hour 16
Hour 17
Hour 18
Hour 19
Hour 20
Hour 21
Hour 22
Hour 23
Hour 24
NSR32

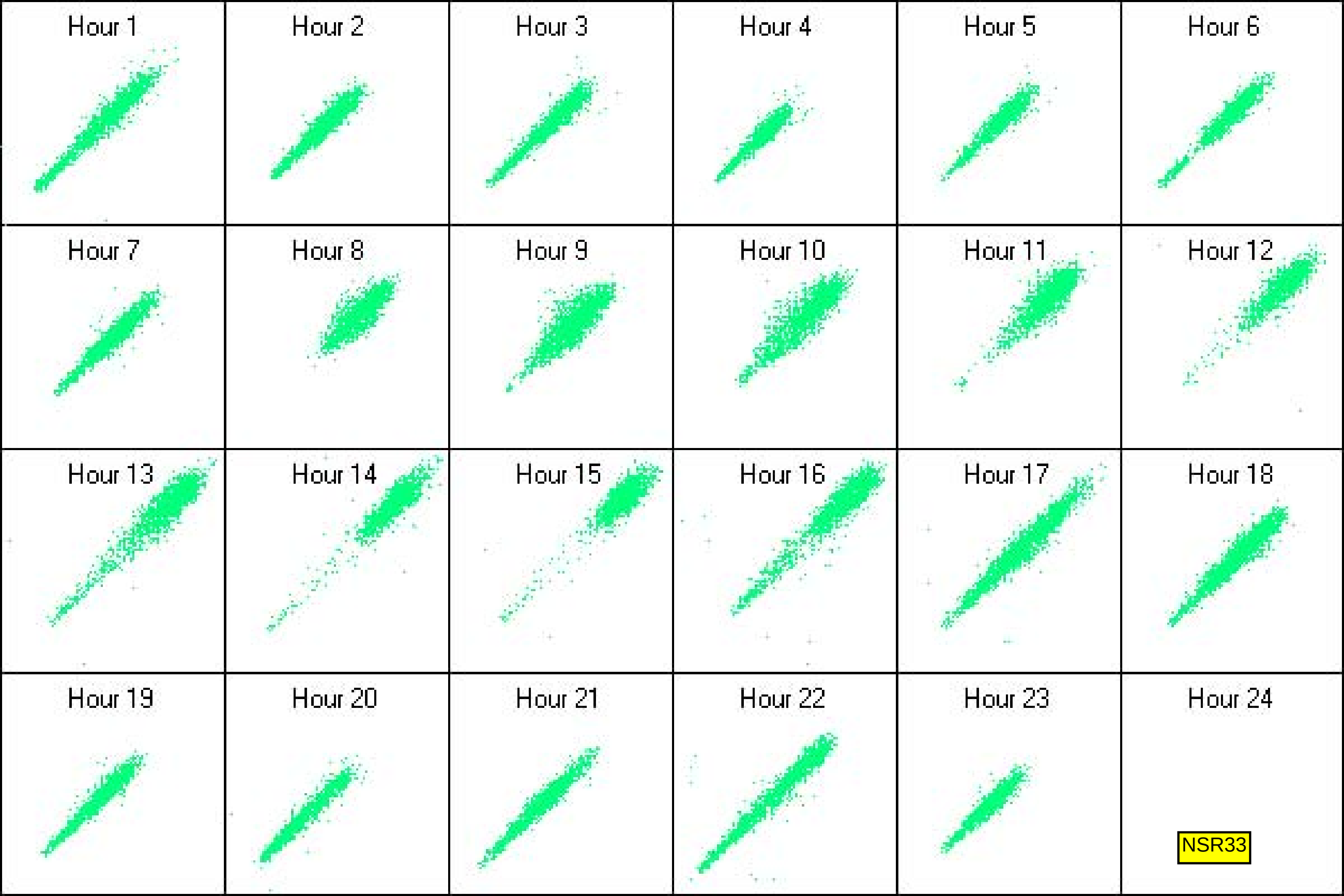

Hour 1
Hour 2
Hour 3
Hour 4
Hour 5
Hour 6
Hour 7
Hour 8
Hour 9
Hour 10
Hour 11
Hour 12
Hour 13
Hour 14
Hour 15
Hour 16
Hour 17
Hour 18
Hour 19
Hour 20
Hour 21
Hour 22
Hour 23
Hour 24
NSR33

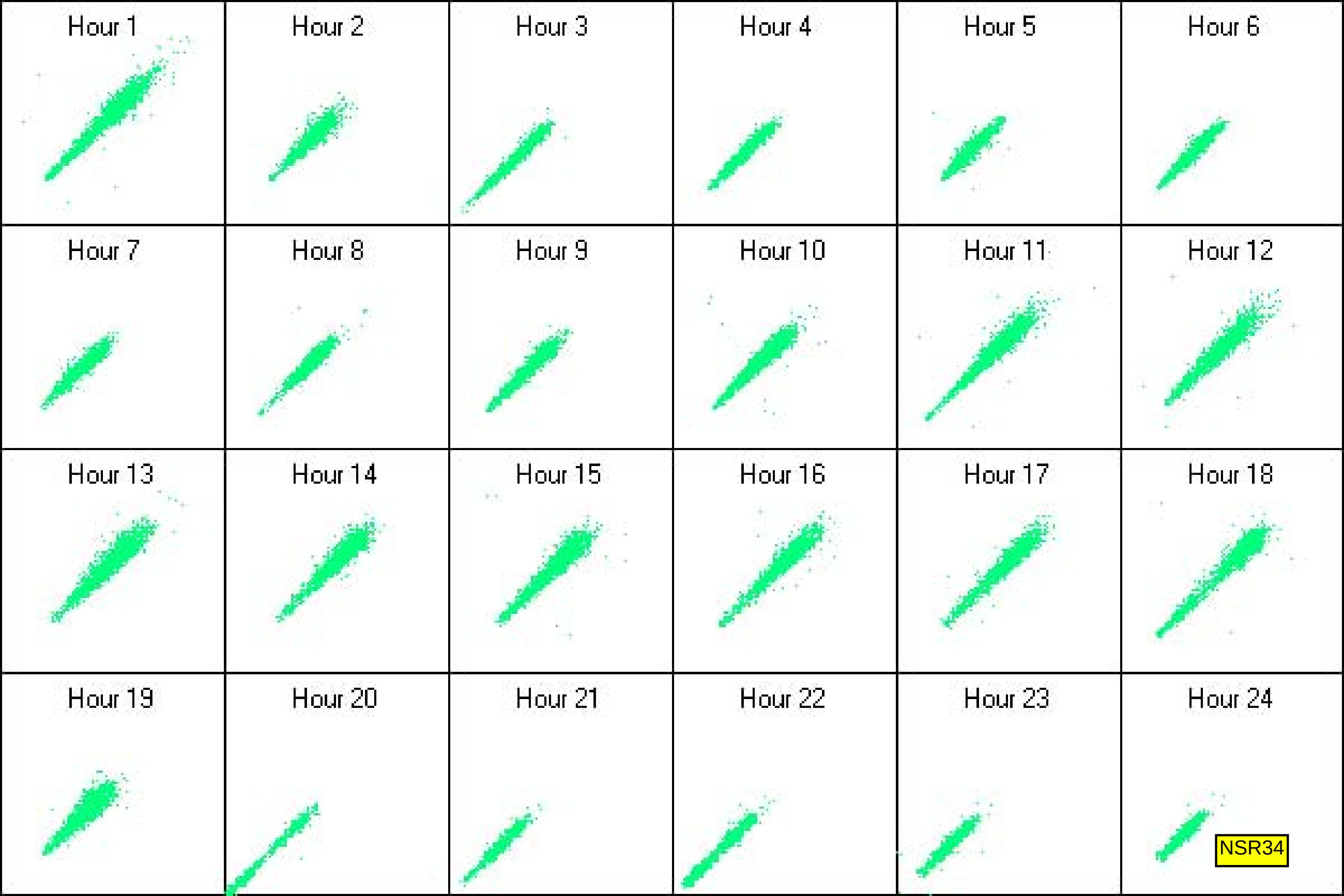
Hour 1
Hour 2
Hour 3
Hour 4
Hour 5
Hour 6
Hour 7
Hour 8
Hour 9
Hour 10
Hour 11
Hour 12
Hour 13
Hour 14
Hour 15
Hour 16
Hour 17
Hour 18
Hour 19
Hour 20
Hour 21
Hour 22
Hour 23
Hour 24
NSR34

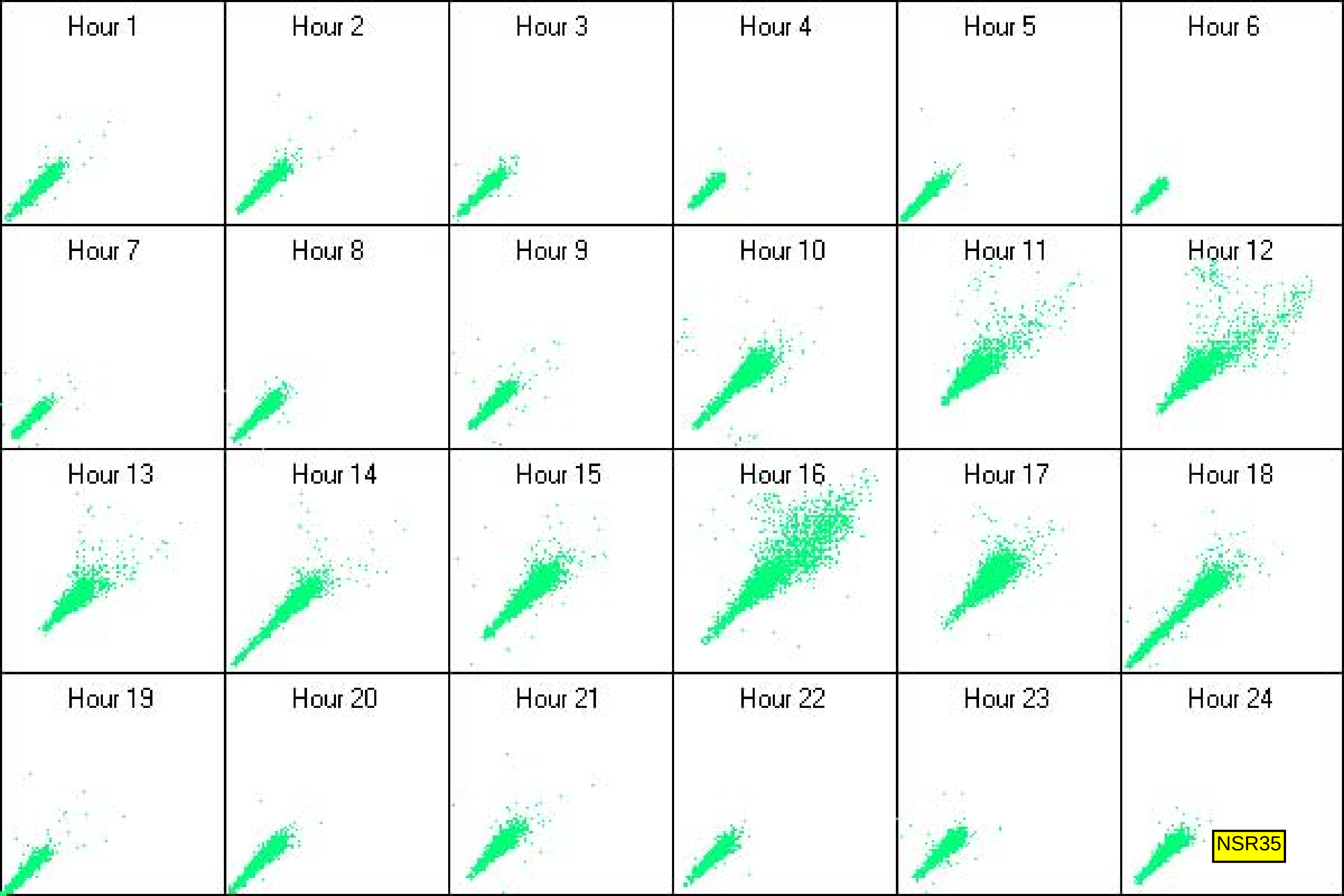
Hour 1
Hour 2
Hour 3
Hour 4
Hour 5
Hour 6
Hour 7
Hour 8
Hour 9
Hour 10
Hour 11
Hour 12
Hour 13
Hour 14
Hour 15
Hour 16
Hour 17
Hour 18
Hour 19
Hour 20
Hour 21
Hour 22
Hour 23
Hour 24
NSR35

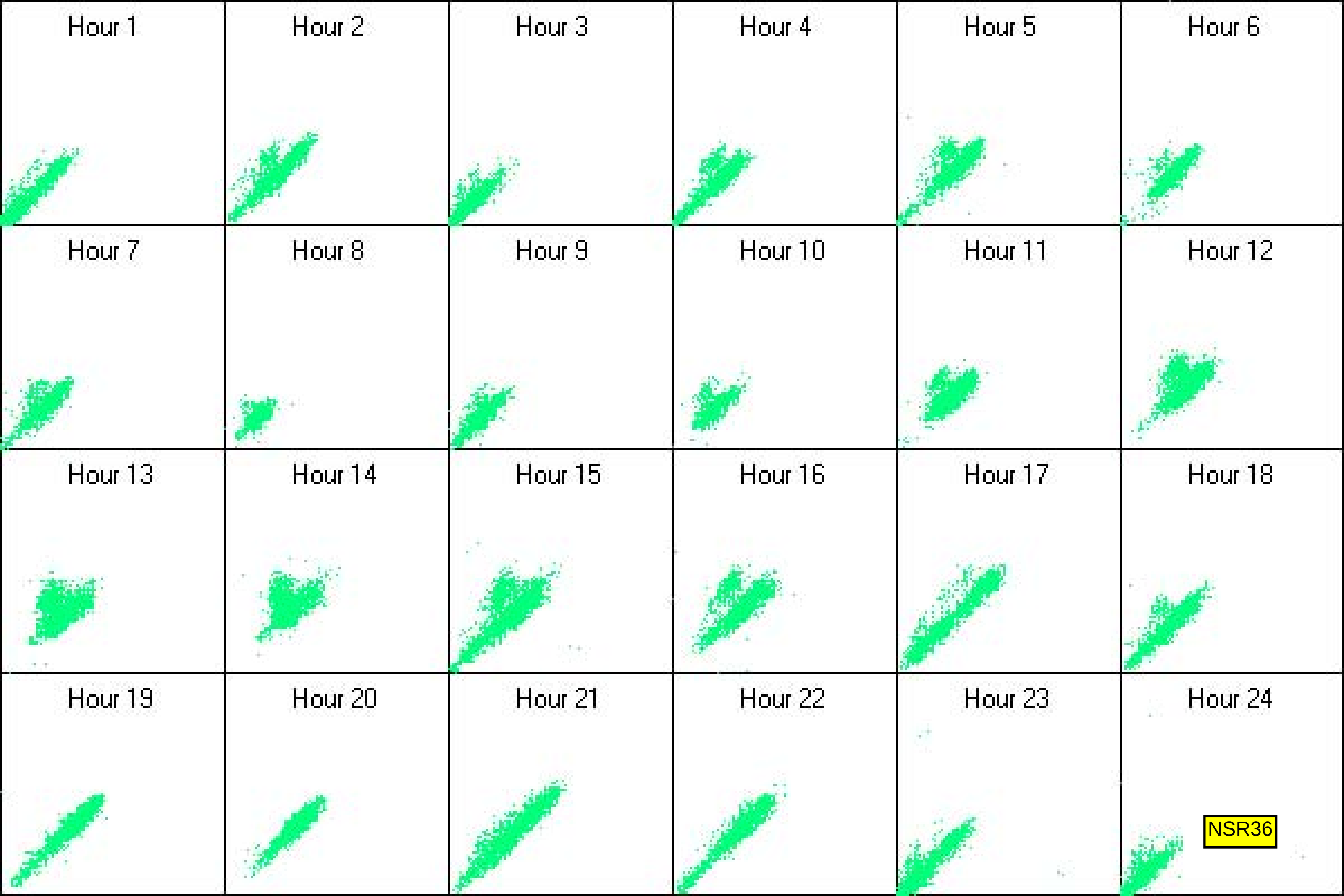

Hour 1
Hour 2
Hour 3
Hour 4
Hour 5
Hour 6
Hour 7
Hour 8
Hour 9
Hour 10
Hour 11
Hour 12
Hour 13
Hour 14
Hour 15
Hour 16
Hour 17
Hour 18
Hour 19
Hour 20
Hour 21
Hour 22
Hour 23
Hour 24
NSR36

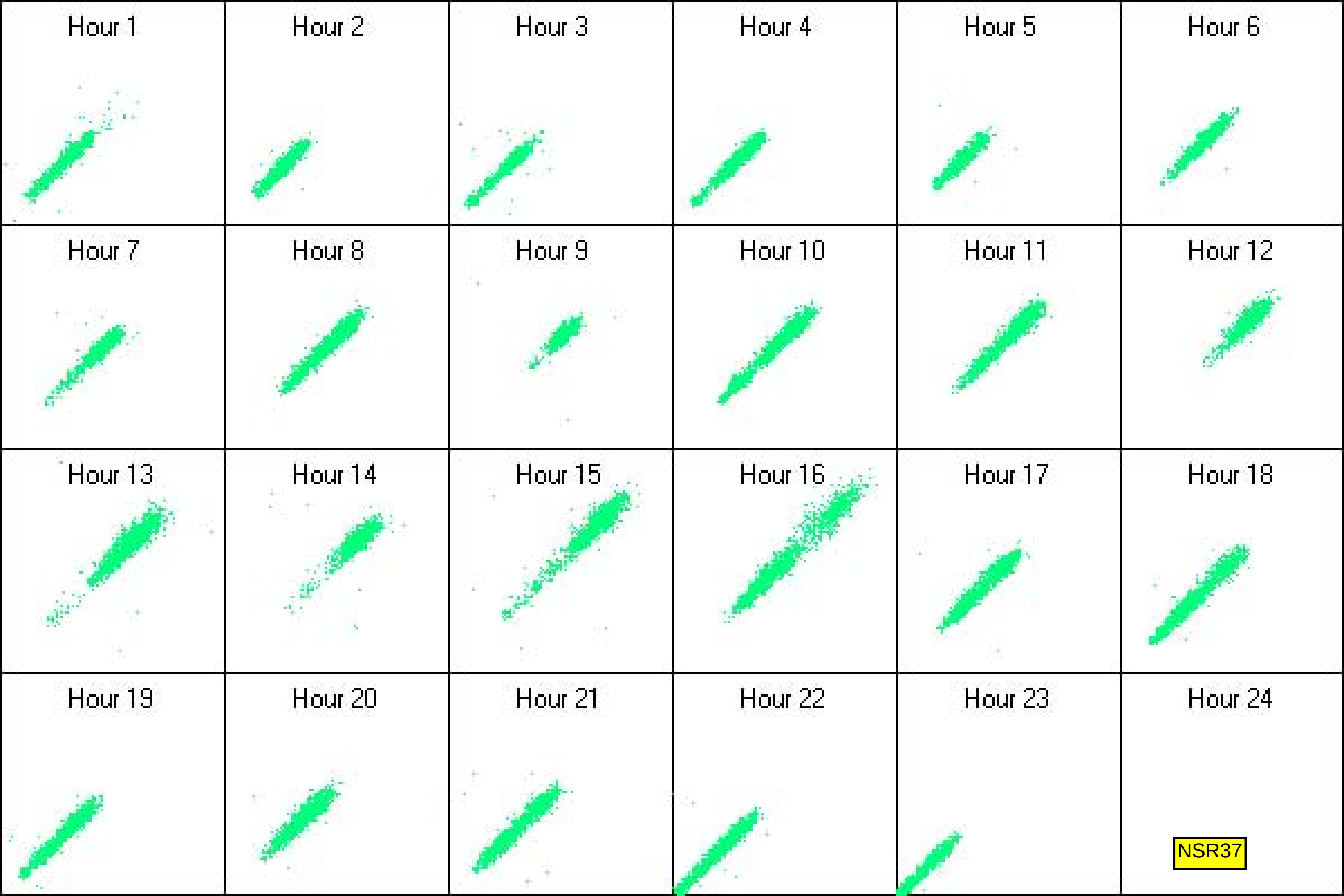
Hour 1
Hour 2
Hour 3
Hour 4
Hour 5
Hour 6
Hour 7
Hour 8
Hour 9
Hour 10
Hour 11
Hour 12
Hour 13
Hour 14
Hour 15
Hour 16
Hour 17
Hour 18
Hour 19
Hour 20
Hour 21
Hour 22
Hour 23
Hour 24
NSR37

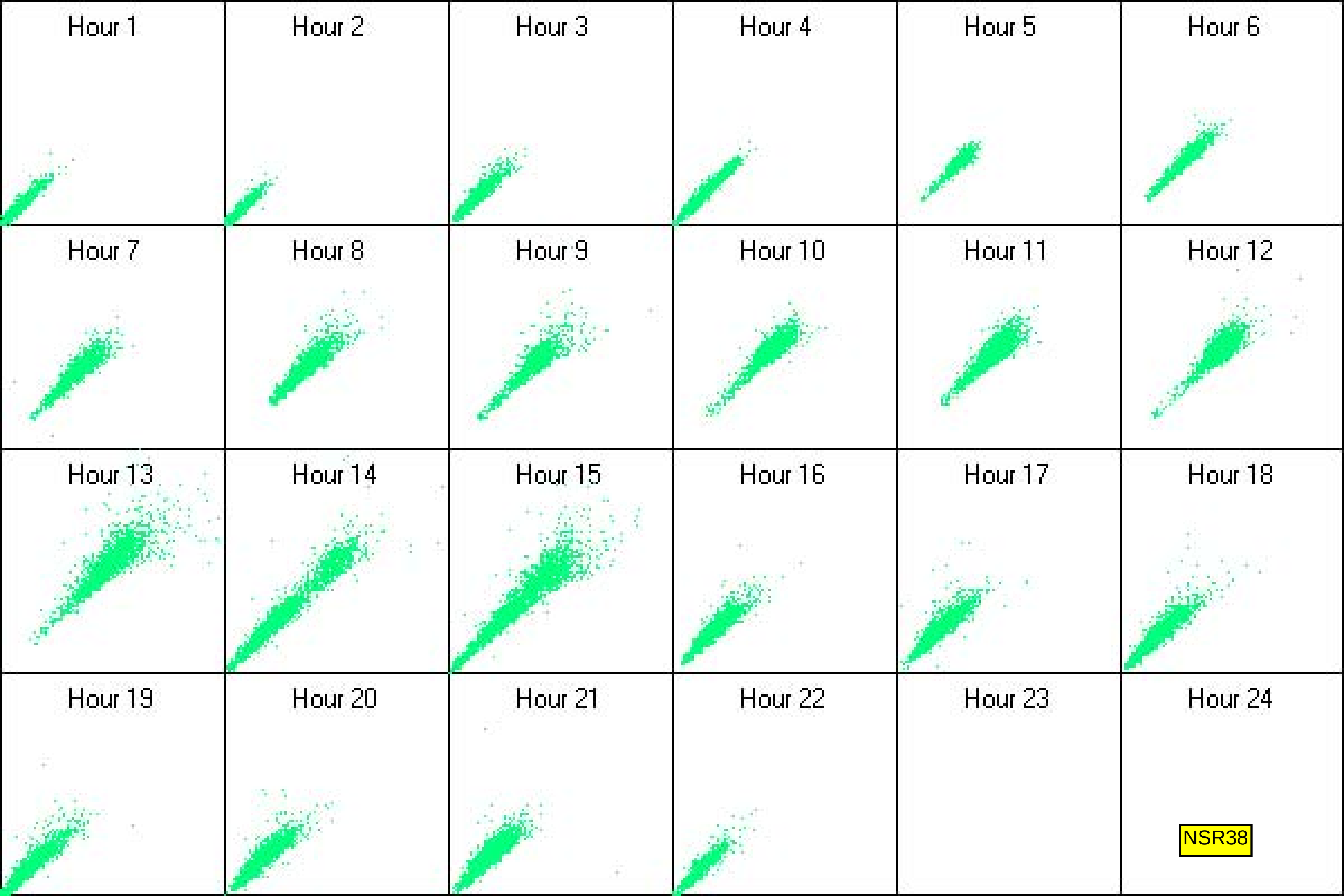
Hour 1
Hour 2
Hour 3
Hour 4
Hour 5
Hour 6
Hour 7
Hour 8
Hour 9
Hour 10
Hour 11
Hour 12
Hour 13
Hour 14
Hour 15
Hour 16
Hour 17
Hour 18
Hour 19
Hour 20
Hour 21
Hour 22
Hour 23
Hour 24
NSR38

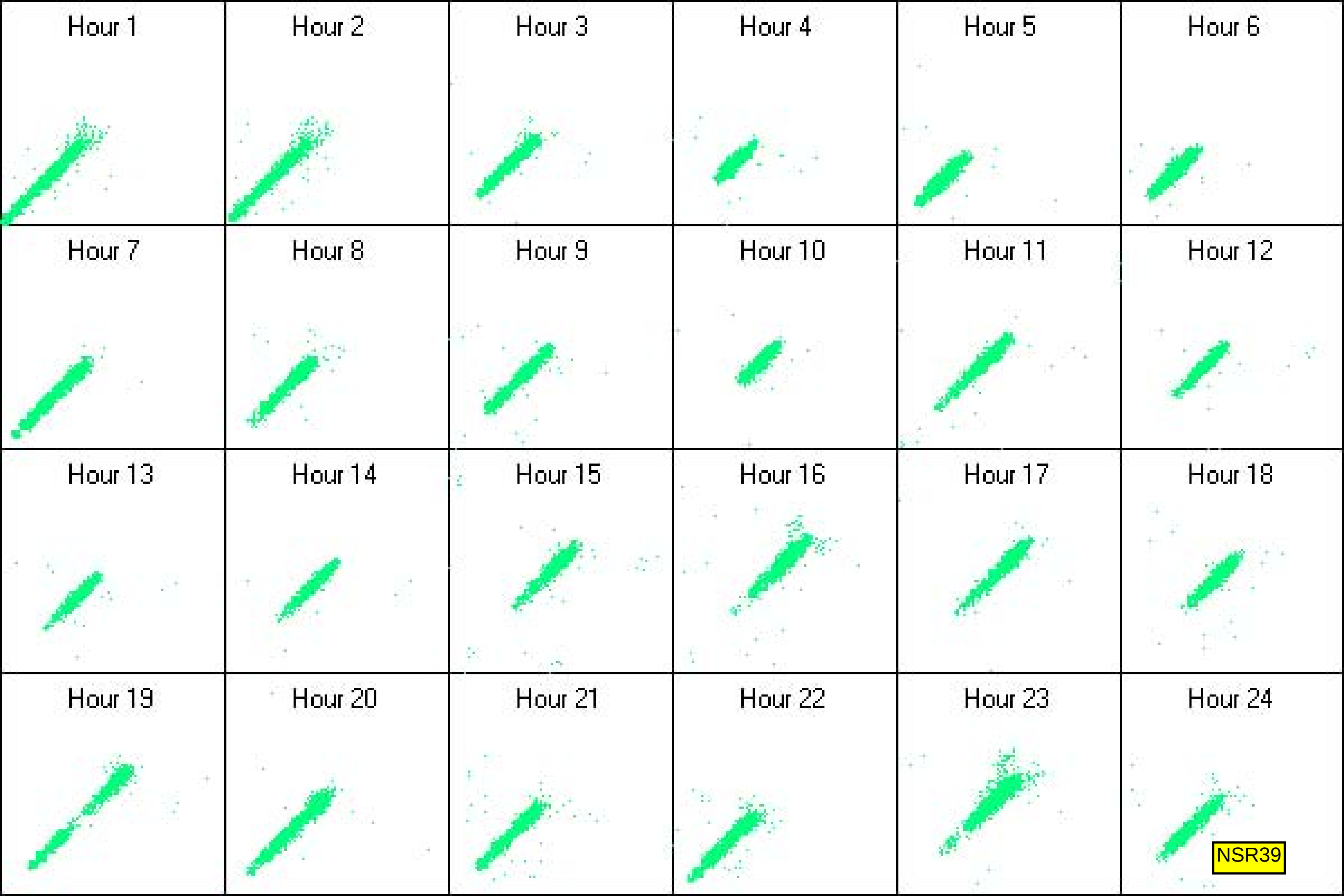
Hour 1
Hour 2
Hour 3
Hour 4
Hour 5
Hour 6
Hour 7
Hour 8
Hour 9
Hour 10
Hour 11
Hour 12
Hour 13
Hour 14
Hour 15
Hour 16
Hour 17
Hour 18
Hour 19
Hour 20
Hour 21
Hour 22
Hour 23
Hour 24
NSR39

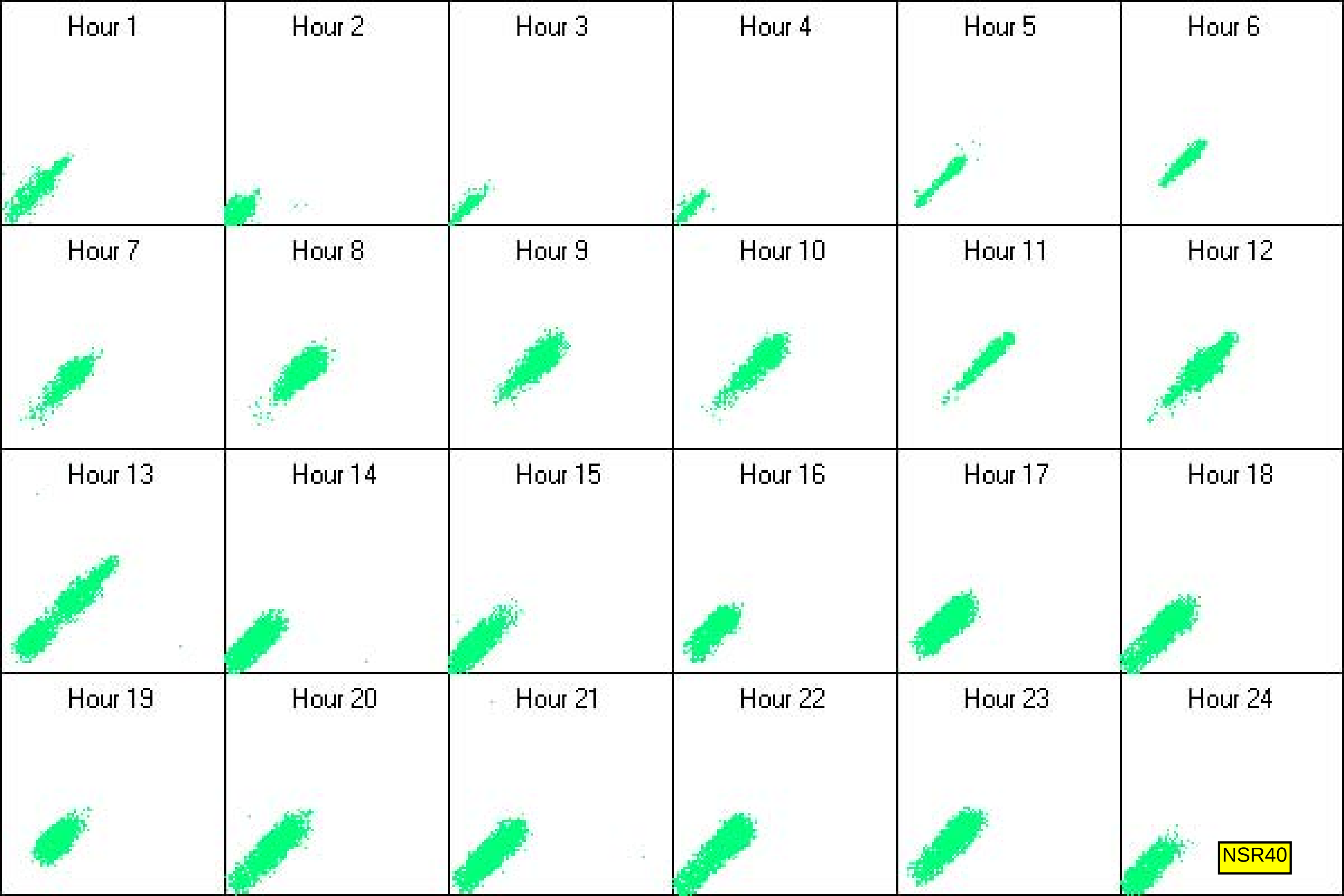

Hour 1
Hour 2
Hour 3
Hour 4
Hour 5
Hour 6
Hour 7
Hour 8
Hour 9
Hour 10
Hour 11
Hour 12
Hour 13
Hour 14
Hour 15
Hour 16
Hour 17
Hour 18
Hour 19
Hour 20
Hour 21
Hour 22
Hour 23
Hour 24
NSR40

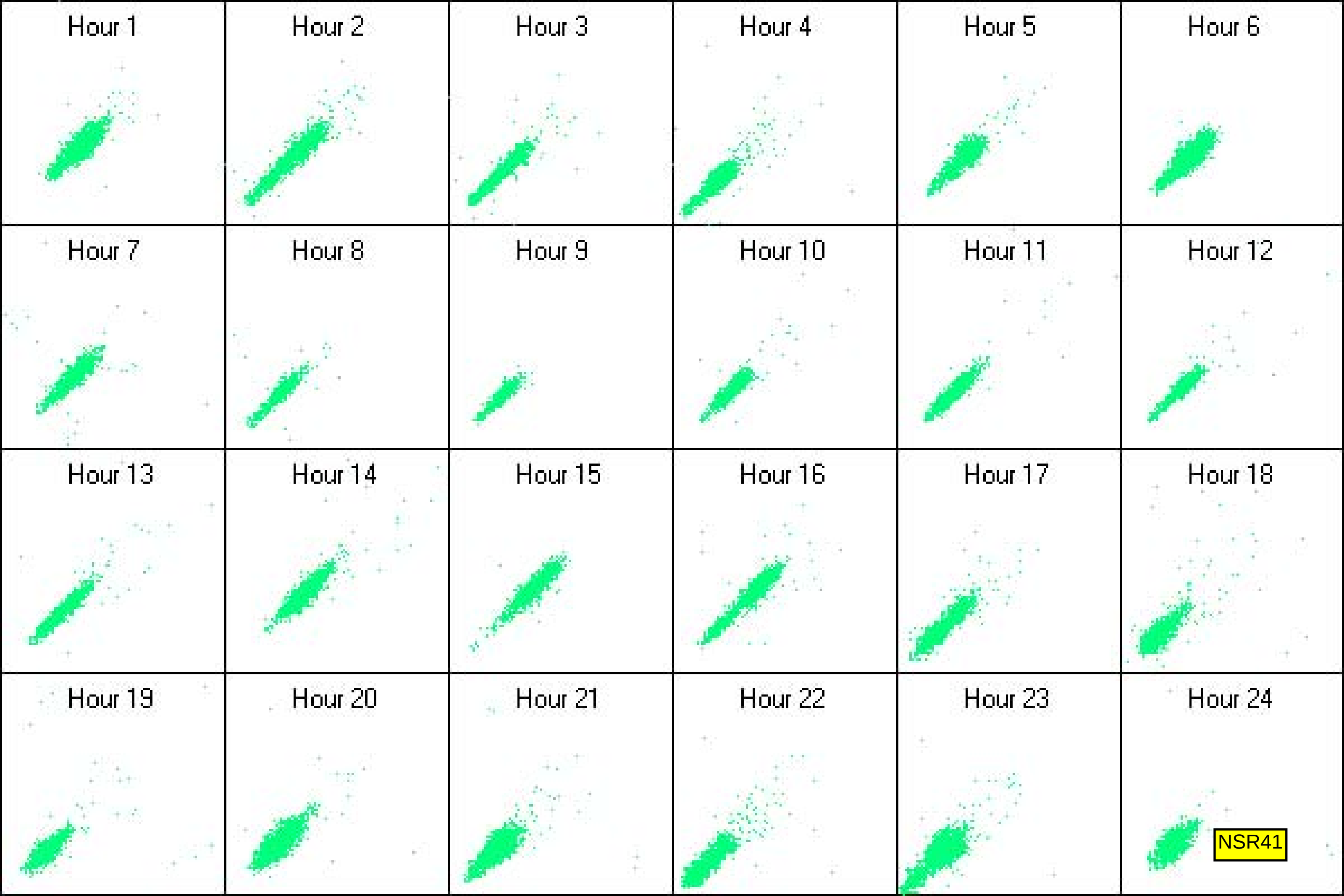
Hour 1
Hour 2
Hour 3
Hour 4
Hour 5
Hour 6
Hour 7
Hour 8
Hour 9
Hour 10
Hour 11
Hour 12
Hour 13
Hour 14
Hour 15
Hour 16
Hour 17
Hour 18
Hour 19
Hour 20
Hour 21
Hour 22
Hour 23
Hour 24
NSR41

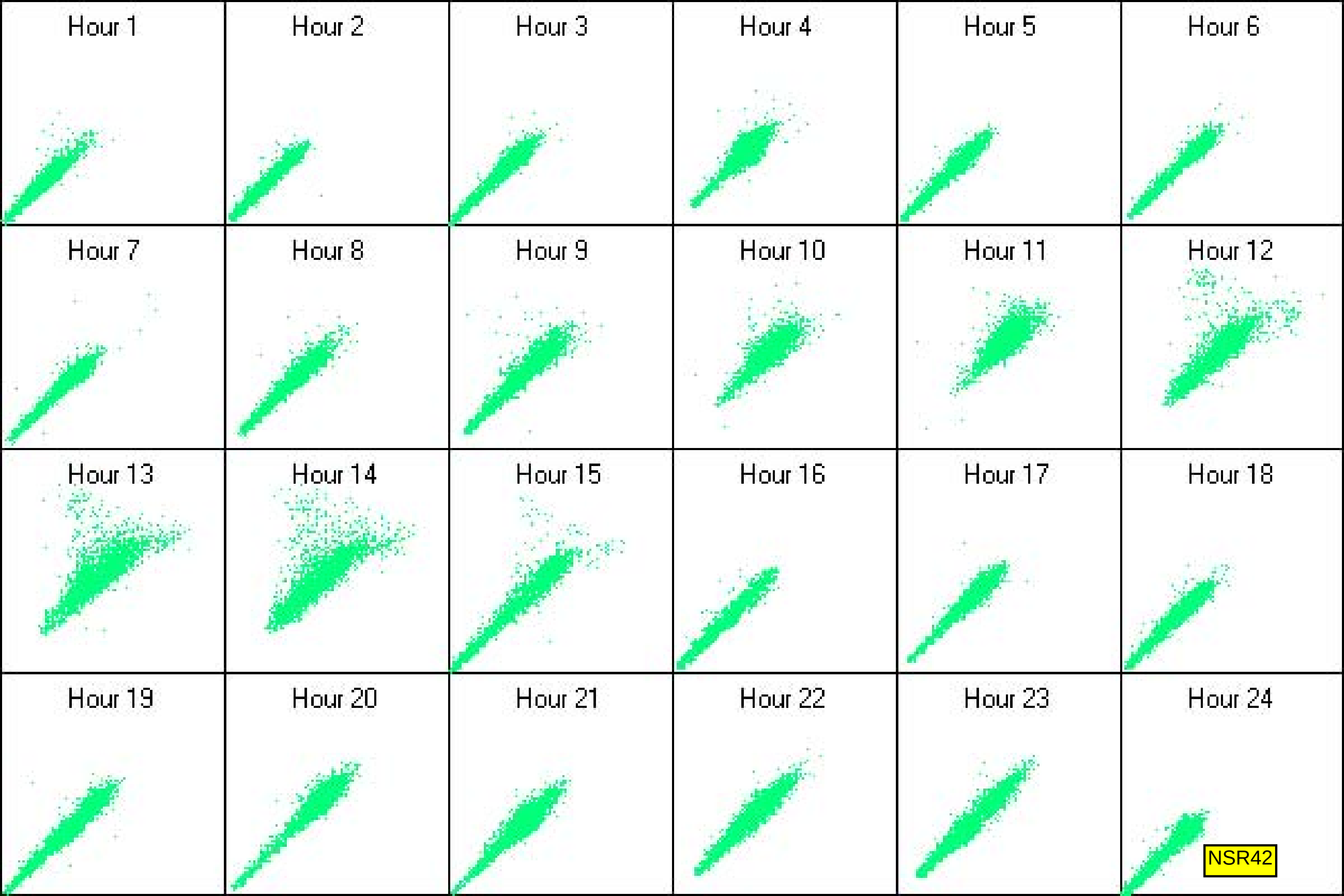
Hour 1
Hour 2
Hour 3
Hour 4
Hour 5
Hour 6
Hour 7
Hour 8
Hour 9
Hour 10
Hour 11
Hour 12
Hour 13
Hour 14
Hour 15
Hour 16
Hour 17
Hour 18
Hour 19
Hour 20
Hour 21
Hour 22
Hour 23
Hour 24
NSR42

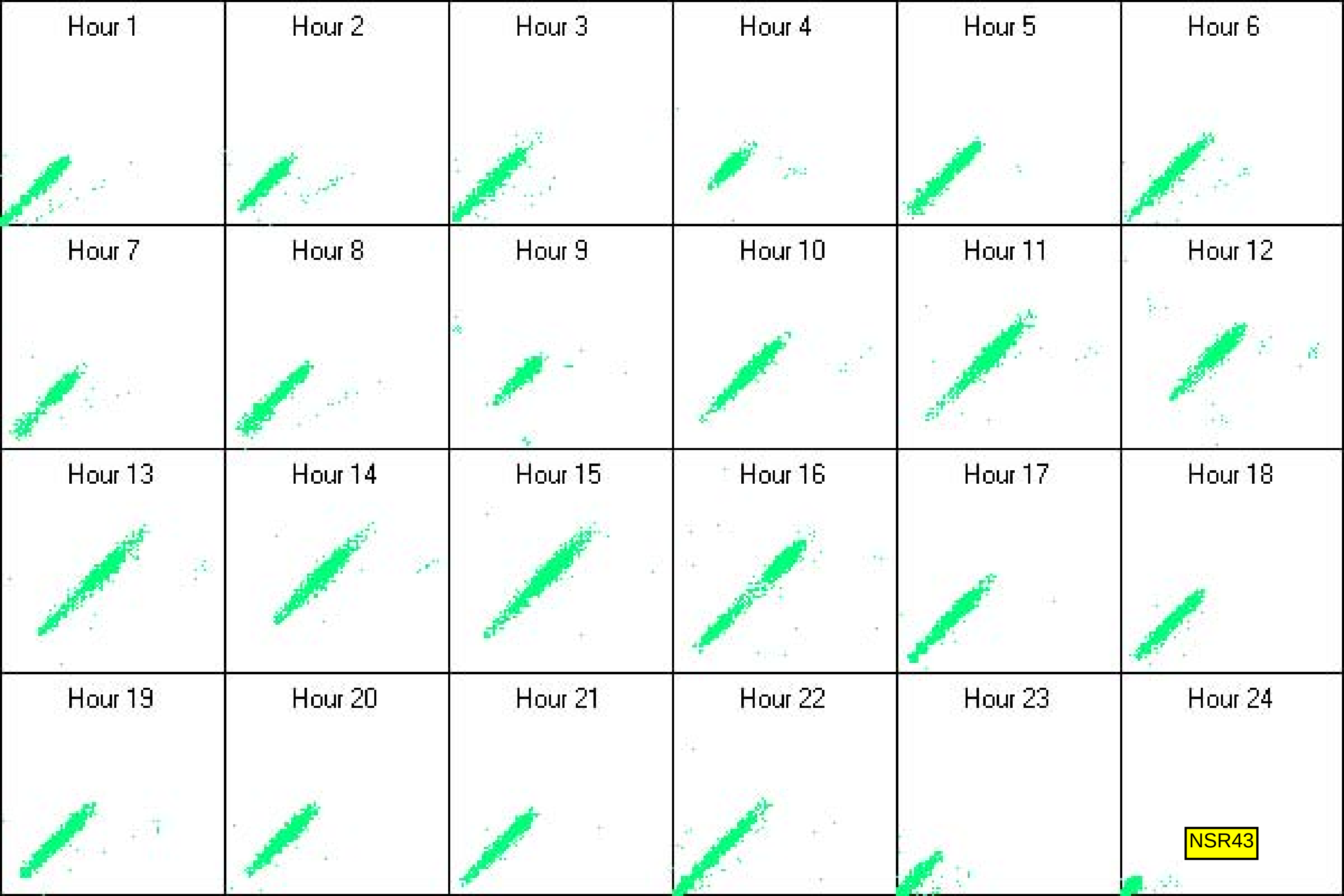
Hour 1
Hour 2
Hour 3
Hour 4
Hour 5
Hour 6
Hour 7
Hour 8
Hour 9
Hour 10
Hour 11
Hour 12
Hour 13
Hour 14
Hour 15
Hour 16
Hour 17
Hour 18
Hour 19
Hour 20
Hour 21
Hour 22
Hour 23
Hour 24
NSR43

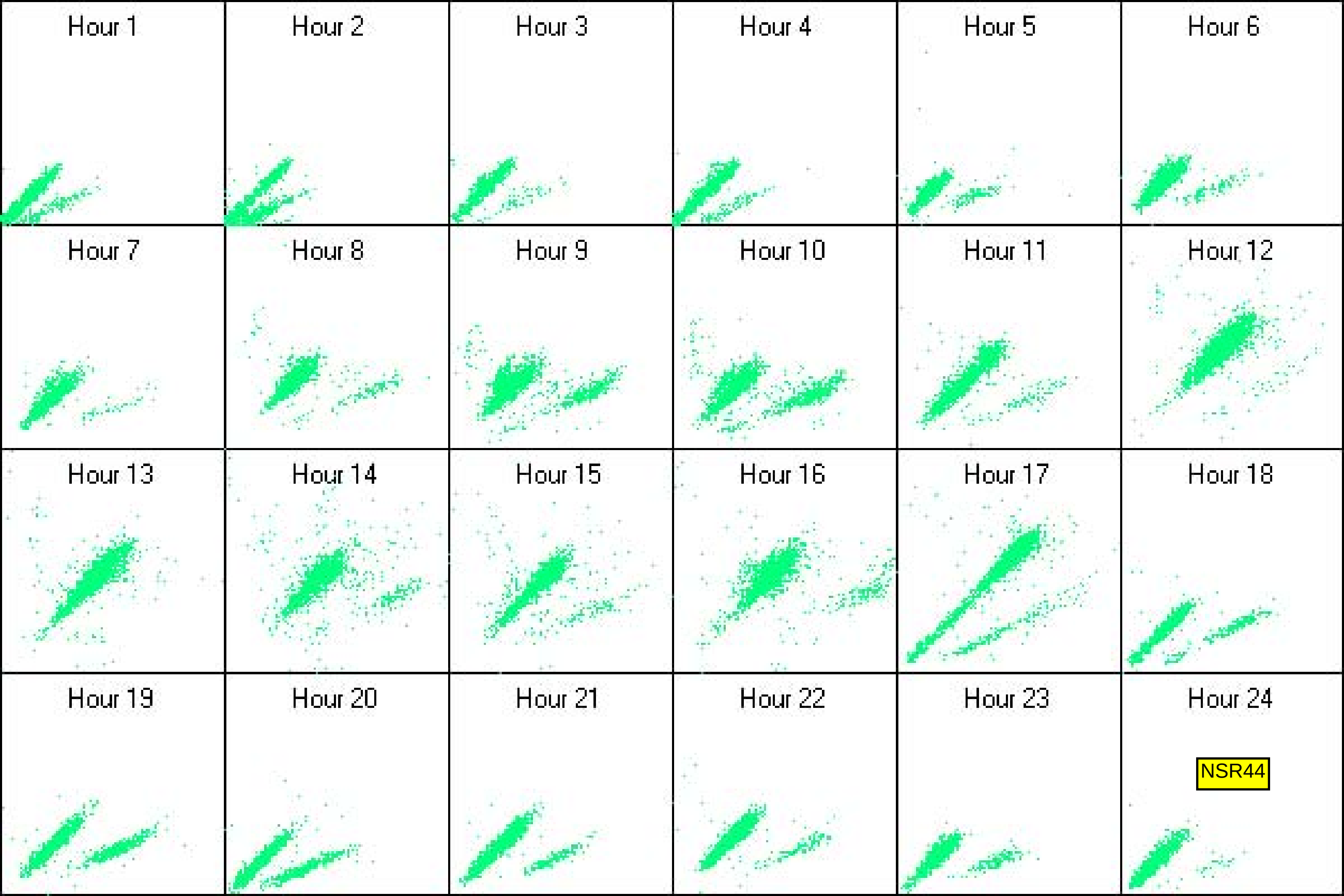
Hour 1
Hour 2
Hour 3
Hour 4
Hour 5
Hour 6
Hour 7
Hour 8
Hour 9
Hour 10
Hour 11
Hour 12
Hour 13
Hour 14
Hour 15
Hour 16
Hour 17
Hour 18
Hour 19
Hour 20
Hour 21
Hour 22
Hour 23
Hour 24
NSR44

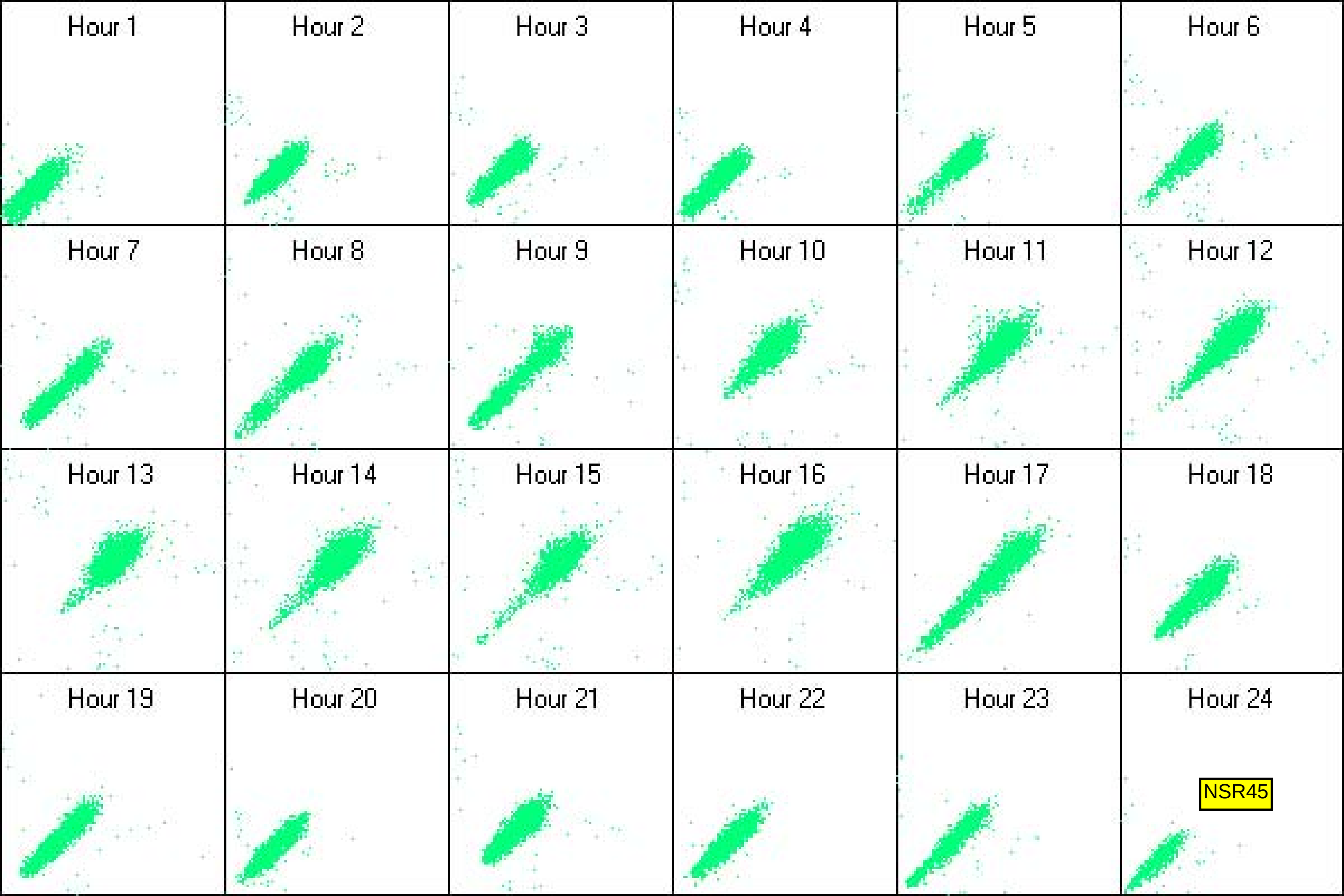
Hour 1
Hour 2
Hour 3
Hour 4
Hour 5
Hour 6
Hour 7
Hour 8
Hour 9
Hour 10
Hour 11
Hour 12
Hour 13
Hour 14
Hour 15
Hour 16
Hour 17
Hour 18
Hour 19
Hour 20
Hour 21
Hour 22
Hour 23
Hour 24
NSR45

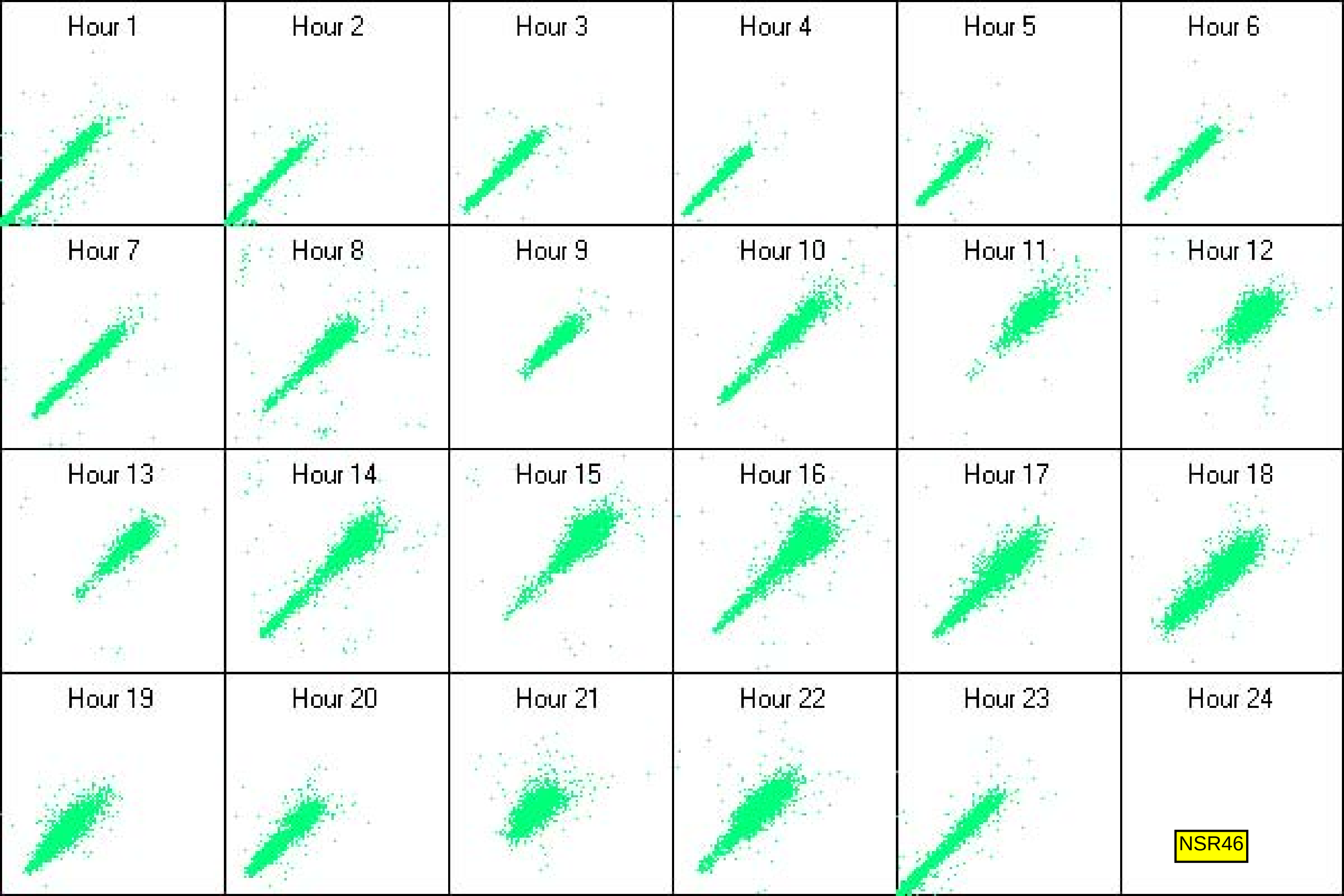
Hour 1
Hour 2
Hour 3
Hour 4
Hour 5
Hour 6
Hour 7
Hour 8
Hour 9
Hour 10
Hour 11
Hour 12
Hour 13
Hour 14
Hour 15
Hour 16
Hour 17
Hour 18
Hour 19
Hour 20
Hour 21
Hour 22
Hour 23
Hour 24
NSR46

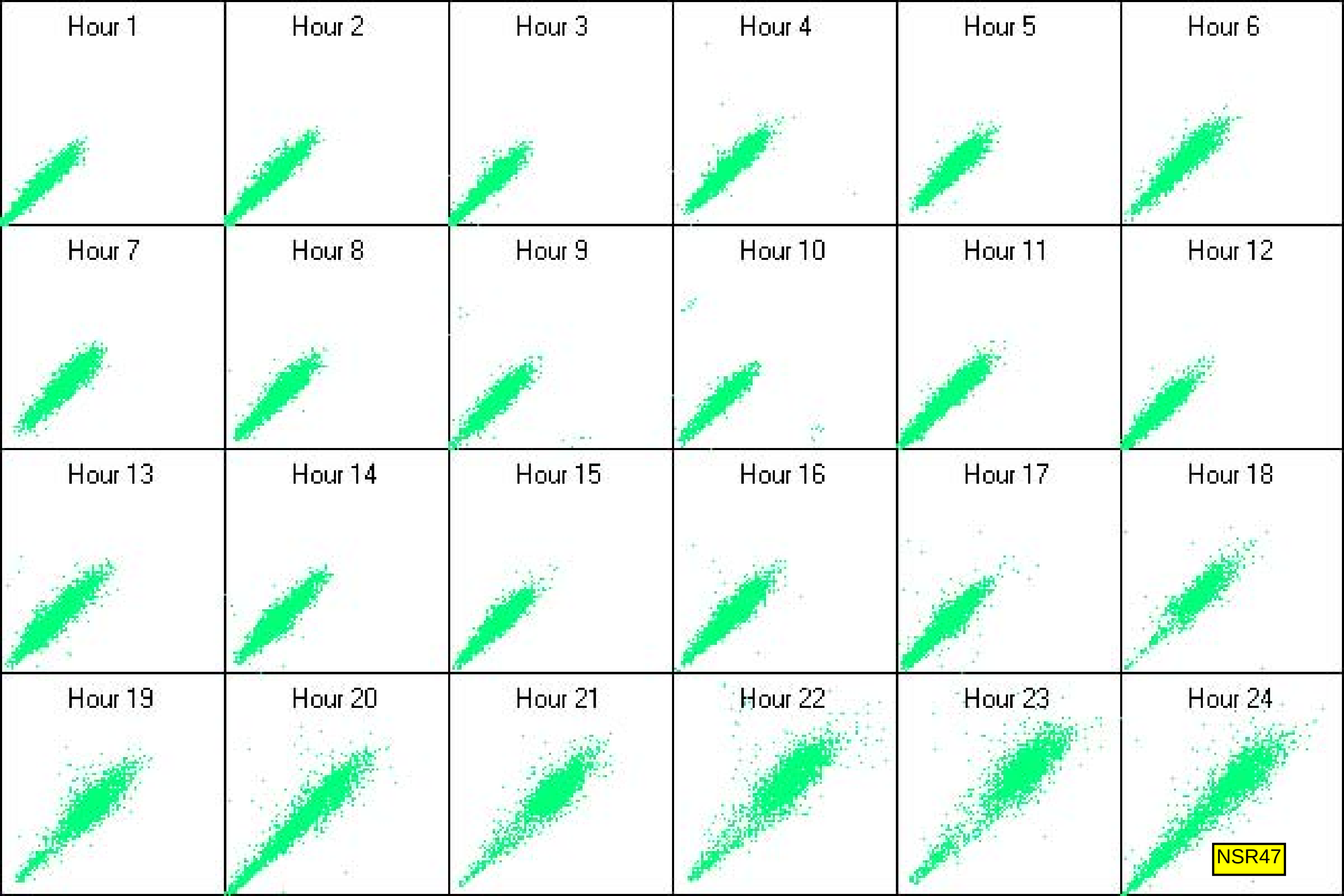
Hour 1
Hour 2
Hour 3
Hour 4
Hour 5
Hour 6
Hour 7
Hour 8
Hour 9
Hour 10
Hour 11
Hour 12
Hour 13
Hour 14
Hour 15
Hour 16
Hour 17
Hour 18
Hour 19
Hour 20
Hour 21
Hour 22
Hour 23
Hour 24
NSR47

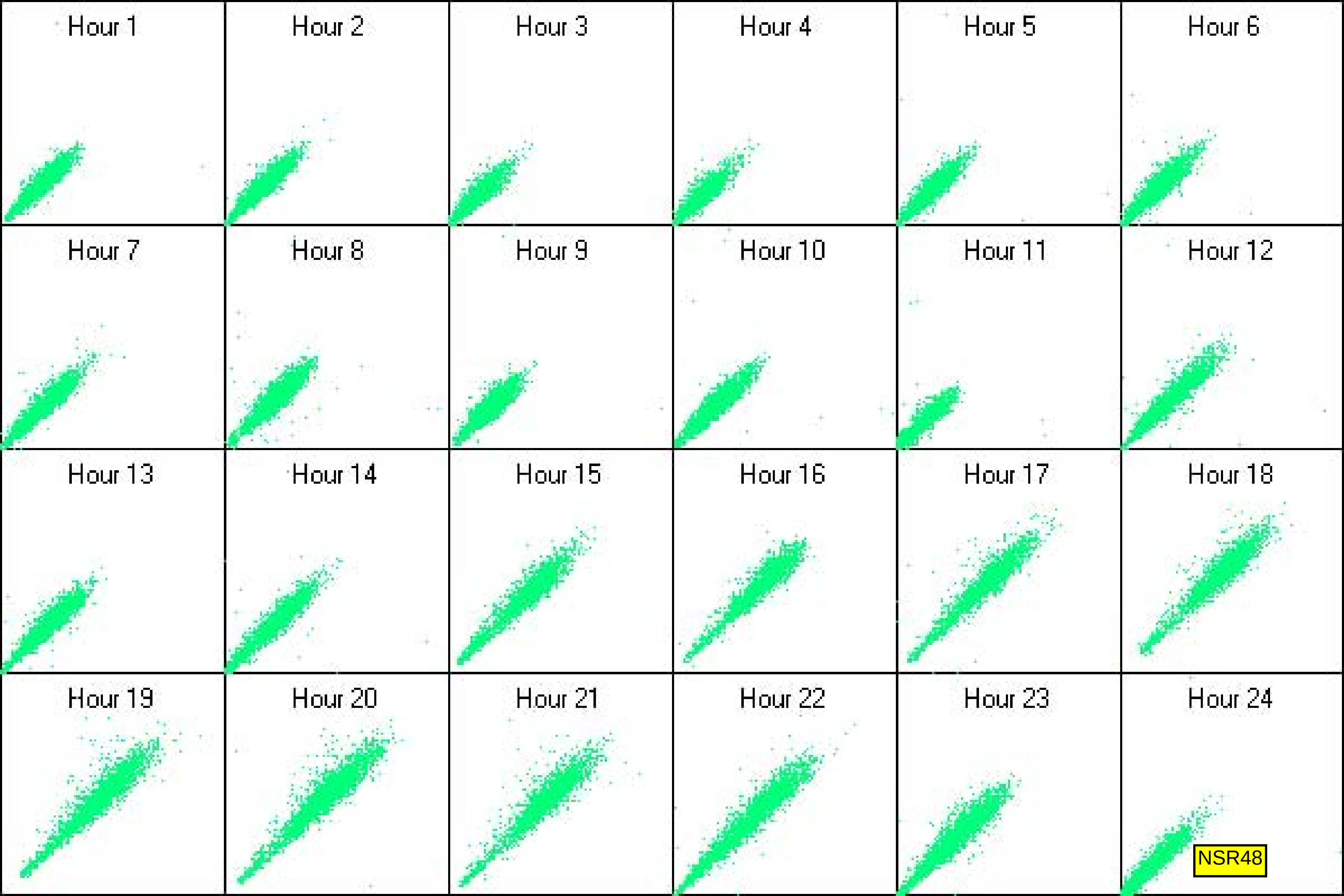
Hour 1
Hour 2
Hour 3
Hour 4
Hour 5
Hour 6
Hour 7
Hour 8
Hour 9
Hour 10
Hour 11
Hour 12
Hour 13
Hour 14
Hour 15
Hour 16
Hour 17
Hour 18
Hour 19
Hour 20
Hour 21
Hour 22
Hour 23
Hour 24
NSR48

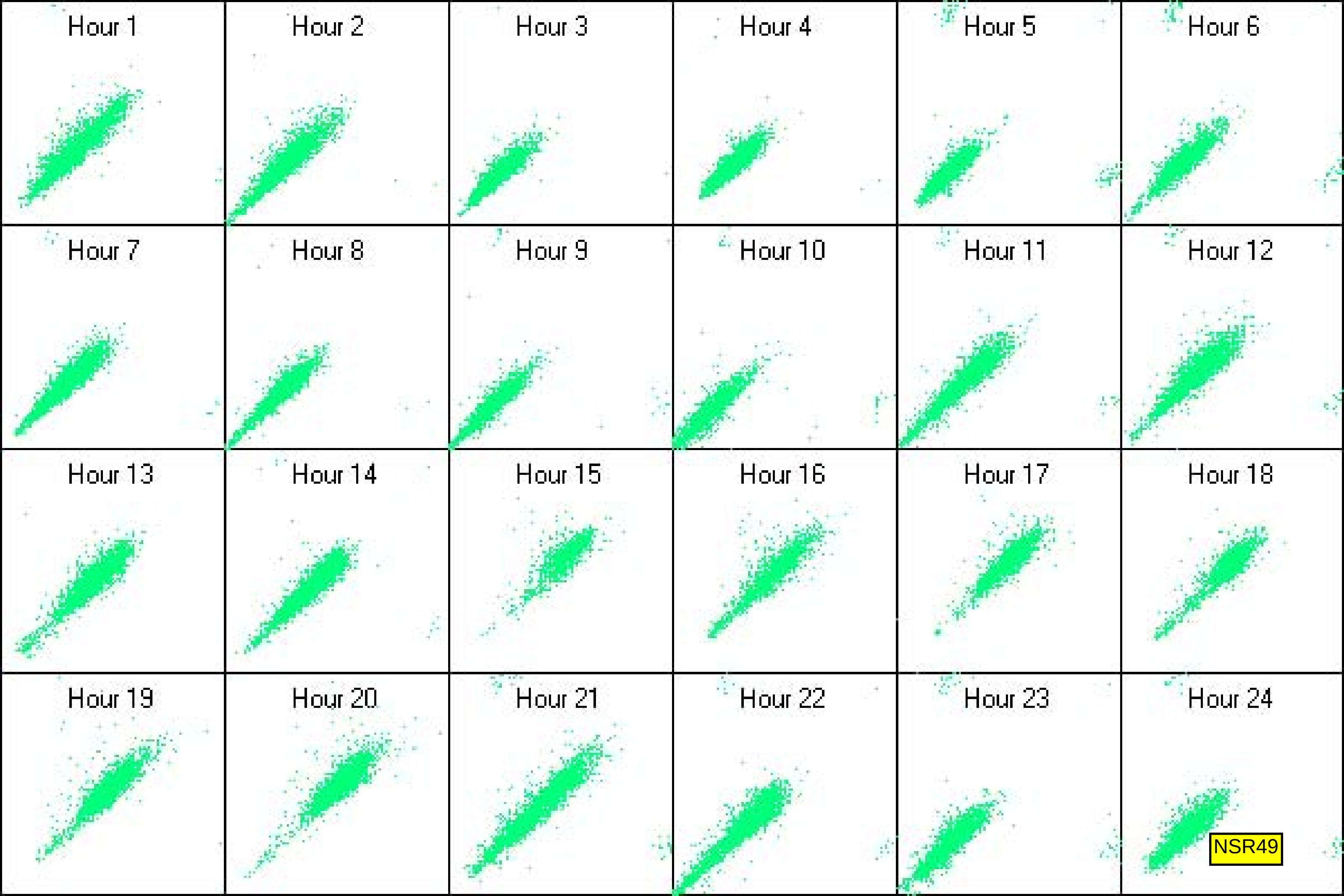

Hour 1
Hour 2
Hour 3
Hour 4
Hour 5
Hour 6
Hour 7
Hour 8
Hour 9
Hour 10
Hour 11
Hour 12
Hour 13
Hour 14
Hour 15
Hour 16
Hour 17
Hour 18
Hour 19
Hour 20
Hour 21
Hour 22
Hour 23
Hour 24
NSR49

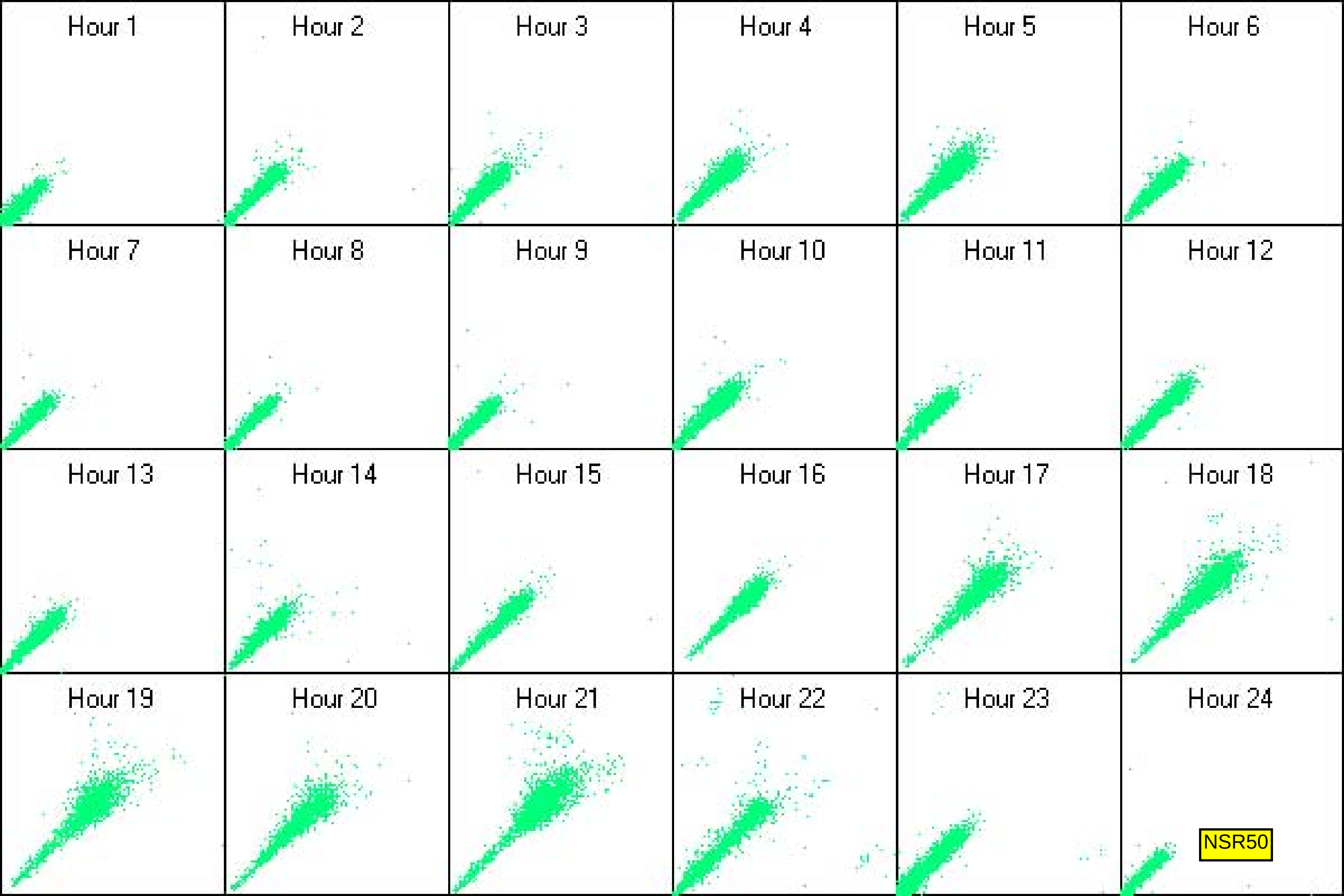
Hour 1
Hour 2
Hour 3
Hour 4
Hour 5
Hour 6
Hour 7
Hour 8
Hour 9
Hour 10
Hour 11
Hour 12
Hour 13
Hour 14
Hour 15
Hour 16
Hour 17
Hour 18
Hour 19
Hour 20
Hour 21
Hour 22
Hour 23
Hour 24
NSR50

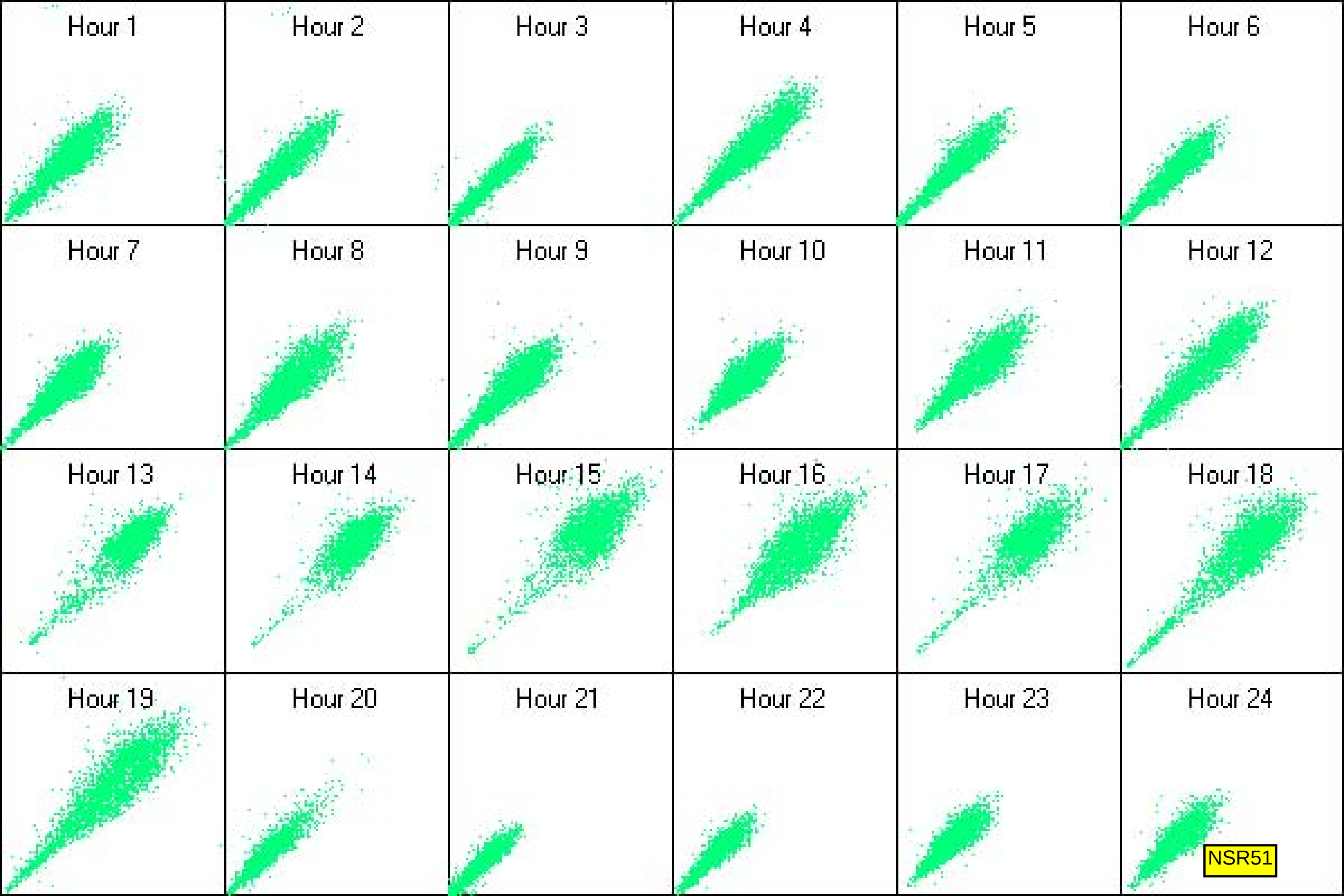
Hour 1
Hour 2
Hour 3
Hour 4
Hour 5
Hour 6
Hour 7
Hour 8
Hour 9
Hour 10
Hour 11
Hour 12
Hour 13
Hour 14
Hour 15
Hour 16
Hour 17
Hour 18
Hour 19
Hour 20
Hour 21
Hour 22
Hour 23
Hour 24
NSR51

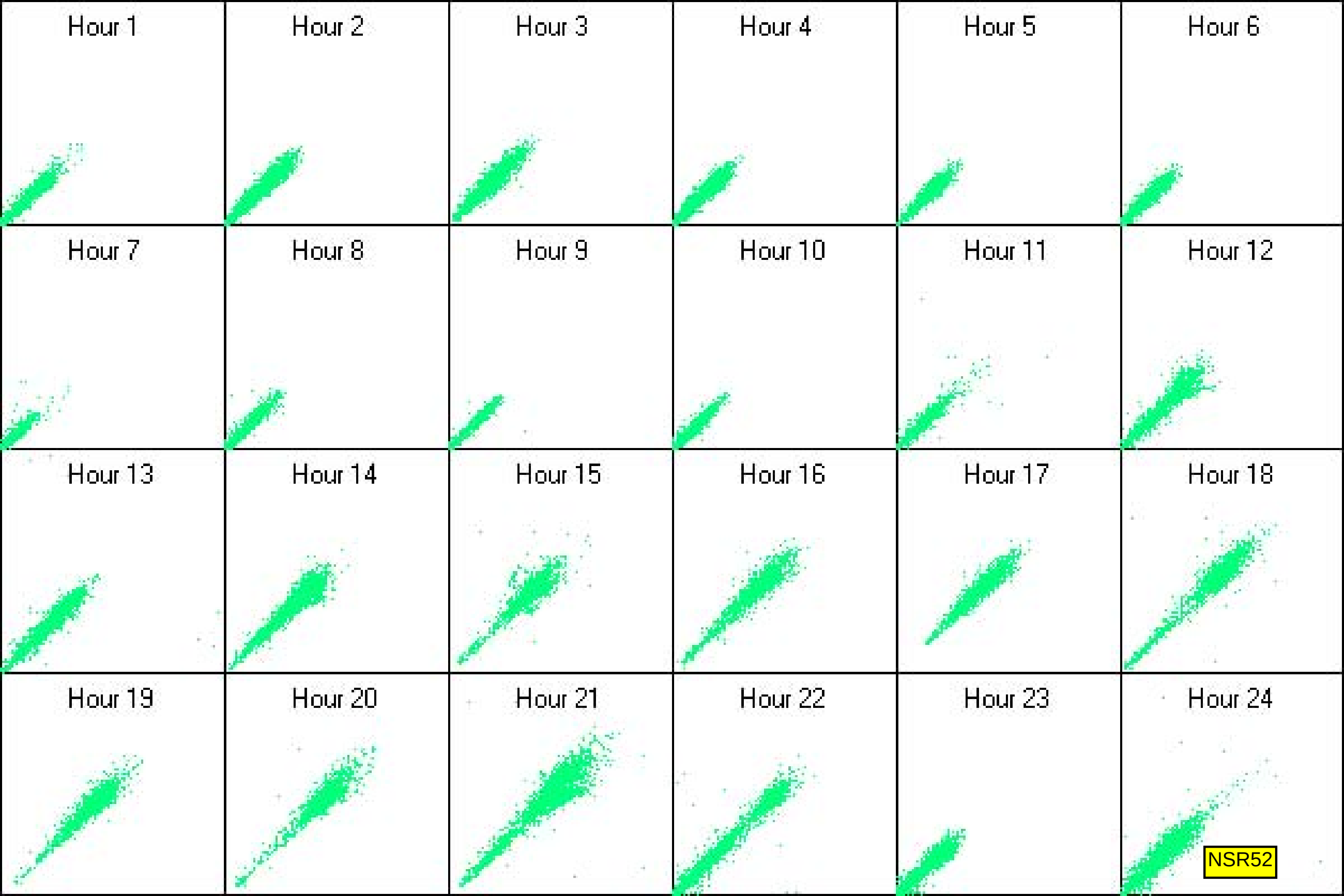
Hour 1
Hour 2
Hour 3
Hour 4
Hour 5
Hour 6
Hour 7
Hour 8
Hour 9
Hour 10
Hour 11
Hour 12
Hour 13
Hour 14
Hour 15
Hour 16
Hour 17
Hour 18
Hour 19
Hour 20
Hour 21
Hour 22
Hour 23
Hour 24
NSR52

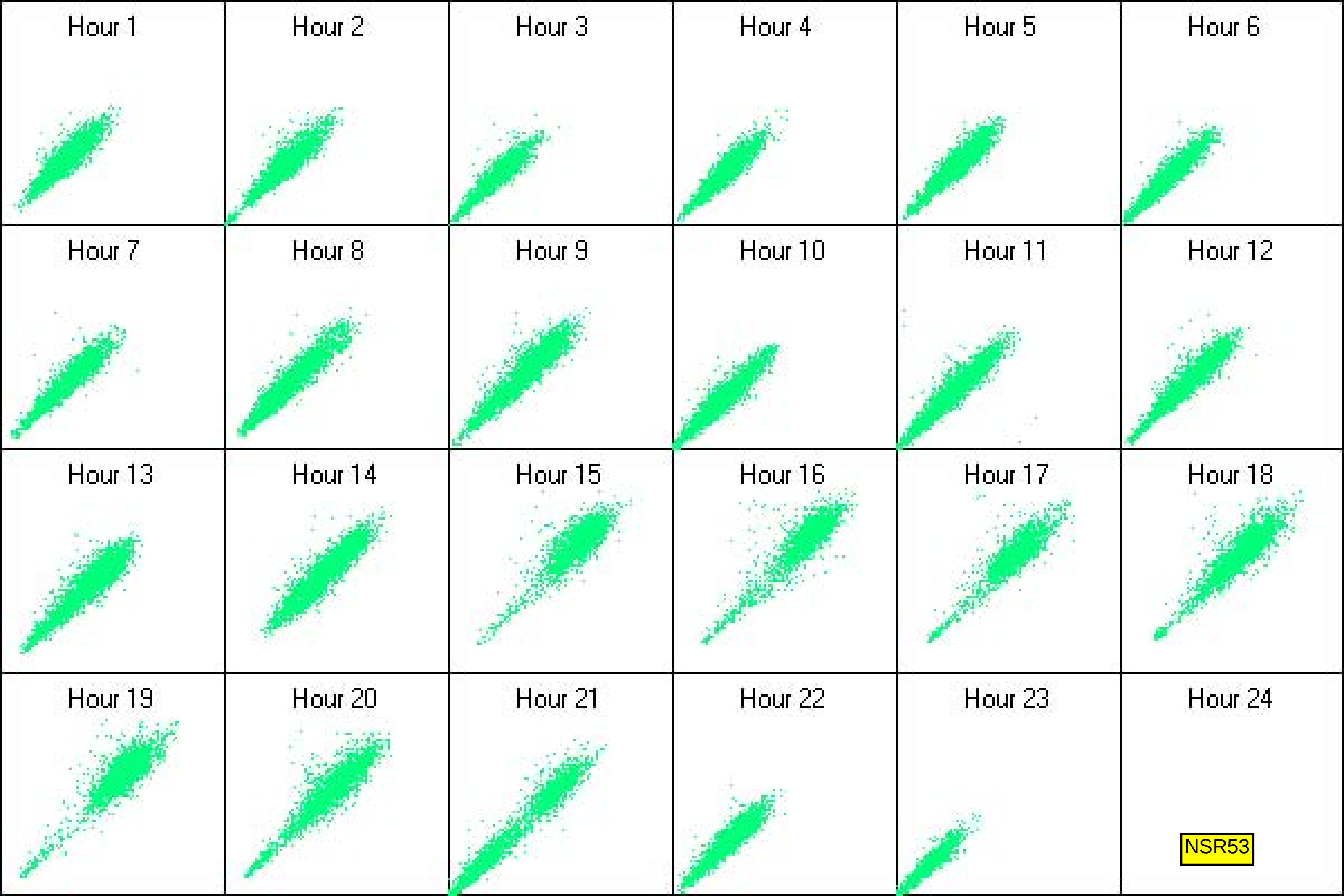
Hour 1
Hour 2
Hour 3
Hour 4
Hour 5
Hour 6
Hour 7
Hour 8
Hour 9
Hour 10
Hour 11
Hour 12
Hour 13
Hour 14
Hour 15
Hour 16
Hour 17
Hour 18
Hour 19
Hour 20
Hour 21
Hour 22
Hour 23
Hour 24
NSR53

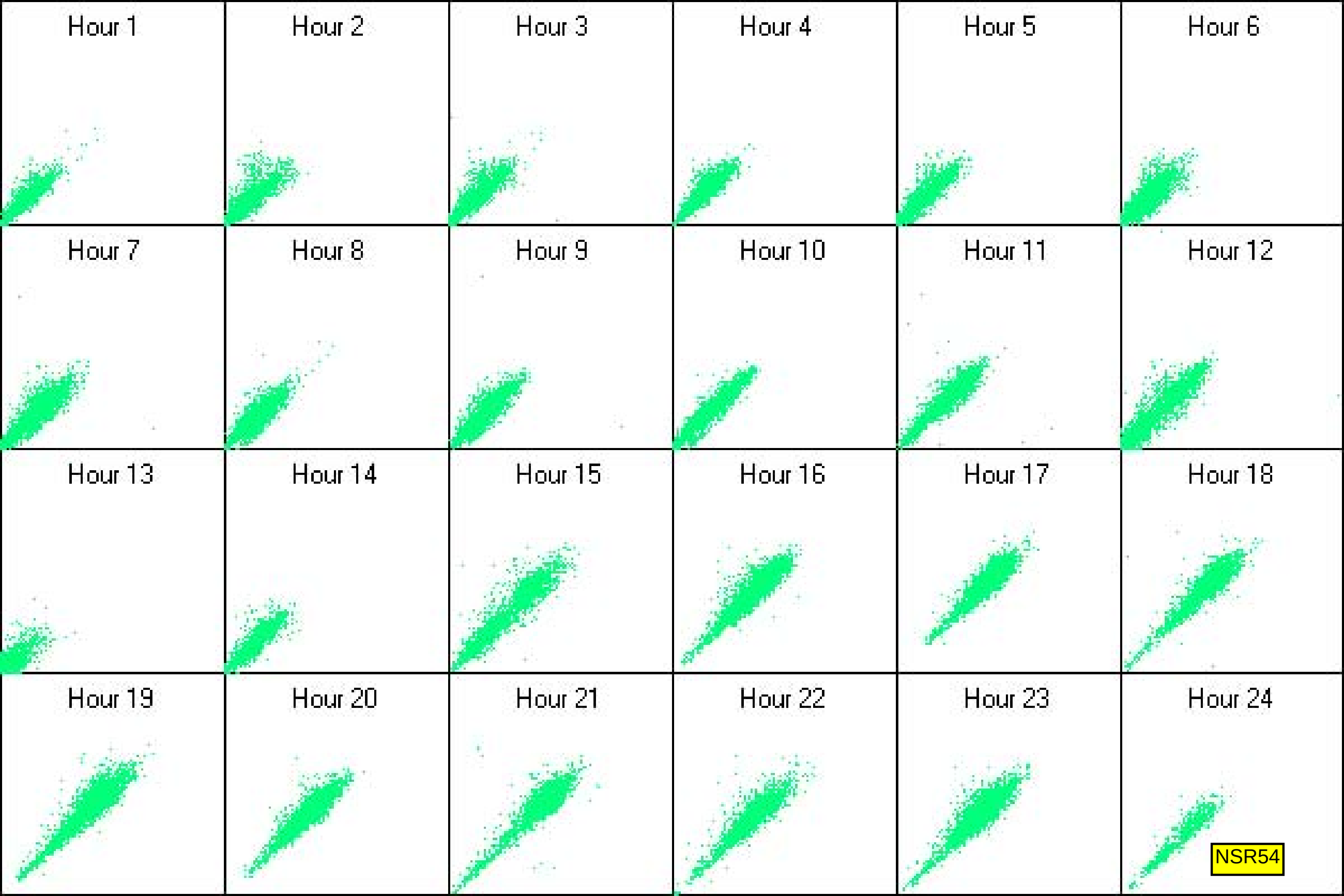
Hour 1
Hour 2
Hour 3
Hour 4
Hour 5
Hour 6
Hour 7
Hour 8
Hour 9
Hour 10
Hour 11
Hour 12
Hour 13
Hour 14
Hour 15
Hour 16
Hour 17
Hour 18
Hour 19
Hour 20
Hour 21
Hour 22
Hour 23
Hour 24
NSR54
